# Comparative Transcriptomic Analysis Reveals Conserved and Nutrient-Specific Responses to Nutrient Deficiencies in Quinoa

**DOI:** 10.3390/plants15182828

**Published:** 2026-09-15

**Authors:** Ashley K. Marcheschi, Bryan G. Hopkins, Shannon V. Nelson, Geneva Bell, Eric N. Jellen, David E. Jarvis, Jonathon T. Hill, Peter J. Maughan

**Affiliations:** 1 Department of Plant and Wildlife Sciences, Brigham Young University, Provo, UT 84602, USA; 2 Department of Cell Biology and Physiology, Brigham Young University, Provo, UT 84602, USA

**Keywords:** *Chenopodium quinoa*, nutrient deficiency, comparative transcriptomics, macronutrients, micronutrients, gene expression, abiotic stress

## Abstract

Nutrient deficiency is a major constraint on crop productivity, yet the molecular mechanisms underlying adaptation to different essential nutrient deficiencies remain poorly understood in quinoa (*Chenopodium quinoa* Willd.). We performed the first comparative transcriptomic analysis of quinoa responses to deficiencies of twelve essential macro- and micronutrients across leaf and root tissues, generating 104 RNA-sequencing libraries analyzed using differential expression, multivariate, co-expression network, and pathway enrichment approaches. Nutrient deficiencies differed substantially in the magnitude and character of their transcriptional responses, with the number of differentially expressed genes ranging from 225 (boron) to 4367 (magnesium) across treatments, and leaves generally exhibiting greater transcriptional plasticity than roots, particularly under nitrogen, potassium, magnesium, and zinc deficiencies. Despite these differences, a conserved transcriptional response centered on protein turnover, ion transport, and nutrient recycling was triggered across nearly all nutrient deficiencies in roots, while suppression of photosynthesis-related genes recurred independently across several deficiencies in leaves; individual deficiencies also elicited distinct regulatory signatures reflecting their physiological functions. Macronutrient deficiencies primarily affected central metabolism, whereas micronutrient deficiencies predominantly altered metal homeostasis, redox balance, and specialized cofactor-dependent pathways. Together, these findings demonstrate that quinoa integrates conserved, tissue-specific stress responses with nutrient-specific regulatory mechanisms to cope with nutrient limitation. This work provides a comparative transcriptomic framework and candidate genes for improving nutrient use efficiency in quinoa and its adaptation to nutrient-limited environments.

## 1. Introduction

Nutrient availability is one of the primary factors limiting agricultural productivity worldwide. Deficiencies of both macro- and micronutrients reduce plant growth, yield, and nutritional quality, and these constraints are expected to intensify as climate change, soil degradation, and increasing food demand place greater pressure on agricultural systems. Improving crop performance under nutrient-limited conditions is therefore an important objective for sustainable agriculture.

Quinoa (*Chenopodium quinoa* Willd.) is an attractive model for studying plant adaptation to nutrient stress because of its exceptional nutritional value and remarkable tolerance to environmental extremes. As a gluten-free pseudocereal with high protein content, a balanced amino acid profile, and elevated concentrations of essential minerals, quinoa has gained global attention as a key contributor to future food security [1,2]. Its ability to thrive under saline, drought-prone, and otherwise marginal growing conditions has expanded its cultivation well beyond its Andean center of origin [3,4]. Nevertheless, even quinoa experiences substantial reductions in growth and productivity under nutrient-limited conditions, highlighting the need to understand the molecular mechanisms underlying nutrient stress adaptation.

Previous studies have documented the physiological and morphological responses of quinoa to nutrient deficiencies under controlled conditions [5], and transcriptomic analyses have begun to characterize responses to individual nutrients. Nitrogen (N) deficiency has been associated with altered amino acid metabolism and flavonoid biosynthesis [6,7], phosphorus (P) deficiency with membrane lipid remodeling [8,9], potassium (K) and iron (Fe) deficiencies with changes in photosynthesis-related pathways [10,11], and zinc (Zn) deficiency with regulation of metal homeostasis [12]. In contrast, transcriptomic characterization of calcium (Ca), magnesium (Mg), sulfur (S), manganese (Mn), copper (Cu), boron (B), and molybdenum (Mo) deficiency remains largely absent from the literature, despite the essential and functionally distinct roles these nutrients play in plant metabolism. Moreover, the studies that do exist have relied on different experimental systems and conditions, making direct comparisons across nutrients difficult.

More importantly, no study has systematically compared transcriptomic responses across the complete spectrum of essential macro- and micronutrient deficiencies within a single experimental framework. Consequently, it remains unclear which transcriptional responses represent conserved adaptations to nutrient stress, and which reflect nutrient-specific regulatory mechanisms. This distinction is critical for identifying common stress-response pathways, understanding nutrient-specific signaling networks, and prioritizing candidate genes for improving nutrient use efficiency in quinoa.

A second, largely unexplored source of variation in nutrient stress responses is tissue identity. Because leaves and roots perform fundamentally different physiological functions, they are also expected to exhibit distinct responses to nutrient limitation. Roots are responsible for nutrient perception and acquisition, whereas leaves integrate nutrient availability with photosynthesis, metabolism, and growth. Previous studies in other plant species have demonstrated strong tissue-specific transcriptional responses to nutrient stress [13,14], but comparable analyses across multiple nutrient deficiencies have not been conducted in quinoa.

Here, we present the first comprehensive tissue-specific transcriptomic comparison of quinoa responses to twelve essential macro- and micronutrient deficiencies: nitrogen, phosphorus, potassium, sulfur, calcium, magnesium, zinc, iron, manganese, copper, boron, and molybdenum. Using RNA sequencing-based differential expression, functional enrichment, multivariate, and co-expression network analyses, we identify both conserved and nutrient-specific transcriptional responses across leaf and root tissues. We hypothesized that nutrient deficiencies would induce a core set of generalized stress responses shared across deficiencies while also activating distinct transcriptional programs that reflect the physiological functions of the limiting nutrient. By establishing a comparative transcriptomic framework spanning all essential mineral nutrients, this study provides new insight into the molecular basis of nutrient stress adaptation in quinoa and identifies candidate pathways and genes for future functional and crop improvement studies.

## 2. Results

### 2.1. Phenotypic Responses to Nutrient Deficiency

**Figure 1:**
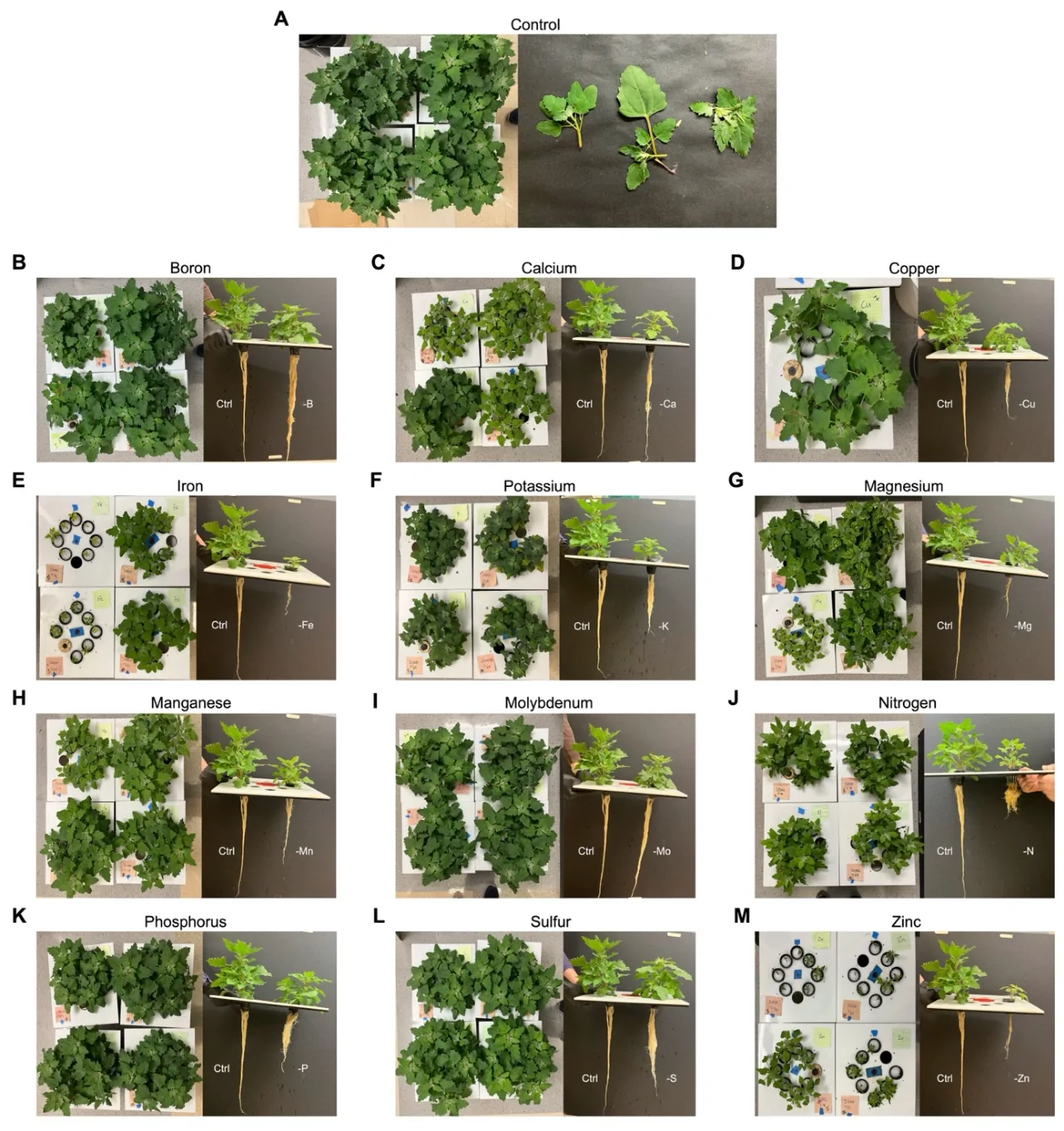
Physical effects of nutrient deficiency on quinoa plants at 30 days after planting. For each panel, all four replicate blocks are shown at left, while a representative single plant from the deficient and control treatments is shown on the right for direct comparison. (**A**) All four positive control blocks and control leaves. (**B**) All boron-deficient (B) blocks, with a representative boron-deficient plant compared to the control. (**C**) All calcium-deficient (Ca) blocks, with a representative calcium-deficient plant compared to the control. (**D**) Copper-deficient (Cu) block I, with a representative copper-deficient plant compared to the control. (**E**) All iron-deficient (Fe) blocks, with a representative iron-deficient plant compared to the control. (**F**) All potassium-deficient (K) blocks, with a representative potassium-deficient plant compared to the control. (**G**) All magnesium-deficient (Mg) blocks, with a representative magnesium-deficient plant compared to the control. (**H**) All manganese-deficient (Mn) blocks, with a representative manganese-deficient plant compared to the control. (**I**) All molybdenum-deficient (Mo) blocks, with a representative molybdenum-deficient plant compared to the control. (**J**) All nitrogen-deficient (N) blocks, with a representative nitrogen-deficient plant compared to the control. (**K**) All phosphorus-deficient (P) blocks, with a representative phosphorus-deficient plant compared to the control. (**L**) All sulfur-deficient (S) blocks, with a representative sulfur-deficient plant compared to the control. (**M**) All zinc-deficient (Zn) blocks, with a representative zinc-deficient plant compared to the control.

A subset of phenotypic measurements relevant to the transcriptomic analyses presented here is reported below; a comprehensive characterization of growth, yield, and tissue nutrient concentrations across all treatments will be presented in a separate, forthcoming study. Deficiencies of individual nutrients produced distinct phenotypic responses after 30 days of growth, ranging from minimal visual symptoms under phosphorus, boron, and molybdenum deficiency to severe chlorosis, reduced biomass, and root stunting under nitrogen, potassium, magnesium, zinc, and iron deficiency (Figure 1; Table 1). Among these five, zinc and iron deficiency produced the most extreme reductions in growth and NDVI, with plants an order of magnitude smaller by shoot dry mass than those under nitrogen, potassium, or magnesium deficiency. Intermediate responses were observed for sulfur, calcium, manganese, and copper deficiencies; notably, manganese-deficient plants showed comparatively low visual ratings despite shoot dry mass comparable to or exceeding that of the control, indicating that visible chlorosis severity did not consistently track with growth suppression for this nutrient. Reductions in shoot growth, root development, and normalized difference vegetation index (NDVI) were generally consistent with this categorization, with the largest reductions observed under zinc and iron deficiency, followed by nitrogen, potassium, and magnesium deficiency, and the smallest under boron and molybdenum deficiency. One notable exception was phosphorus deficiency, which produced visibly denser, more compact root systems despite minimal effects on shoot growth, NDVI, or root dry mass, indicating that structural root changes did not always correspond to measurable biomass reduction. Overall, the severity of visible symptoms generally corresponded to the magnitude of transcriptomic changes observed in subsequent analyses.

### 2.2. RNA Sequencing and Data Quality

RNA sequencing generated high-quality transcriptomic datasets for all 104 leaf and root samples. An average of 36.9 million paired-end reads (73.8 million raw reads; 11.1 Gb) was obtained per sample, with a mean Q30 score of 96.9% (Supplemental Tables S1 and S2). Following quality filtering and adapter trimming, approximately 94% of reads were retained for downstream analyses. Duplicate read levels were elevated across samples (mean 72.2%), likely reflecting sequencing depths exceeding those required for transcriptome quantification.

Trimmed reads were aligned to the *C. quinoa* QQ74 v2.0 reference genome, resulting in an average mapping rate of 92.8%, with 81.2% of reads aligning as properly paired (Supplemental Table S3). Taxonomic screening detected no substantial contamination, with the sum of read percentages mapping to bacterial, viral, or archaeal taxa together averaging 0.5% (Supplemental Table S4). All samples passed quality-control thresholds and were retained for subsequent analyses.

**Figure 2:**
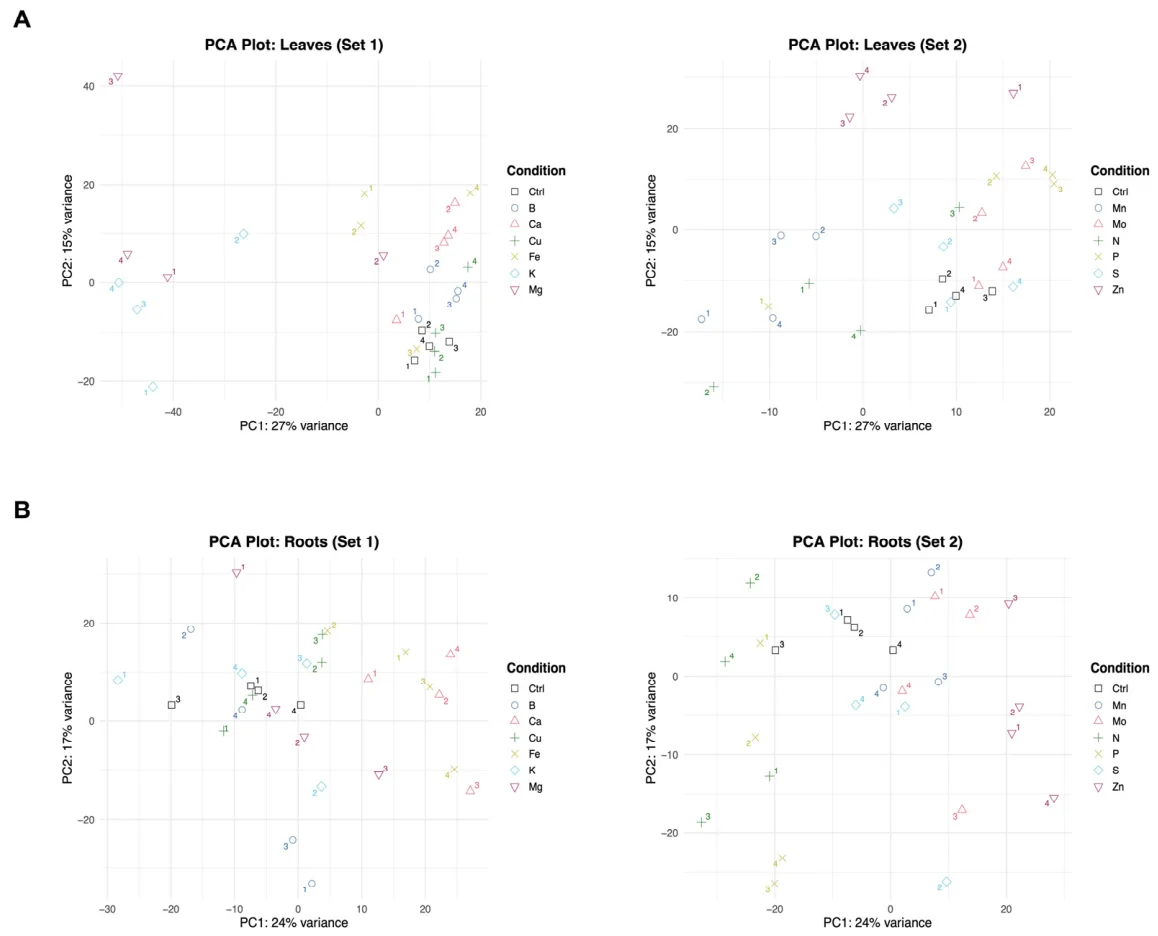
Principal component analysis (PCA) of VST-normalized transcriptomes for leaf and root samples across all nutrient deficiency treatments and the control. (**A**) Score plots for leaf samples, showing the first group of six nutrient deficiency treatments (boron, calcium, copper, control, iron, potassium, and magnesium) (**left**) and the second group of six treatments (manganese, molybdenum, nitrogen, phosphorus, sulfur, and zinc) (**right**), with each point representing an individual biological replicate. (**B**) Score plots for root samples, showing the first group of six nutrient deficiency treatments (boron, calcium, copper, control, iron, and magnesium) (**left**) and the second group of six treatments (manganese, molybdenum, nitrogen, phosphorus, potassium, sulfur, and zinc) (**right**), with each point representing an individual biological replicate.

Nutrient deficiency produced substantial transcriptomic variation in leaf and root tissues. Principal component analysis (PCA) revealed substantial variation across samples, with PC1 and PC2 together explaining 42% of variance in leaf tissue (27% and 15%, respectively) and 41% in root tissue (24% and 17%, respectively) (Figure 2A,B). Treatment and control replicates were broadly dispersed in both tissues, with nutrient-deficient treatments exhibiting considerable overlap and no distinct clustering. This dispersion prompted further examination of sample relationships using correlation analysis. Pearson correlation analysis of all leaf and root samples together showed clear separation by tissue type, with correlation coefficients as low as ~0.65 between leaf and root samples, reflecting fundamental differences in tissue-specific gene expression (Supplemental Figure S1). Within each tissue and treatment, pairwise correlation coefficients among biological replicates ranged from 0.90 to 1.00, with the lowest value (0.90) observed among magnesium-deficient leaf replicates (Supplemental Figures S2 and S3). Consistently high correlation values among biological replicates across all treatments suggest that the variation captured by PCA reflects coordinated differences in subsets of nutrient-responsive genes rather than widespread divergence in transcriptome profiles, technical artifacts, or experimental inconsistency.

Leave-one-out (LOO) analyses were performed for all twelve nutrient-deficiency comparisons in both leaf and root tissue to assess the robustness of differential expression estimates to individual biological replicates (Supplemental Figure S4). Across the majority of nutrient-tissue combinations, log_2_ fold change (log_2_FC) estimates were highly correlated between the full-replicate analysis and each leave-one-out iteration (mean Pearson *r* = 0.80–0.95 across all tested genes), and this correlation increased consistently when restricted to higher-confidence gene sets (adjusted *p*-value [*p*-adj] < 0.05, <0.01, and <0.001), frequently exceeding *r* = 0.95–1.00 among the most confidently identified differentially expressed genes. This pattern indicates that the core transcriptional response to nutrient deficiency was generally stable regardless of which biological replicate was excluded, with sensitivity concentrated among lower-confidence genes near the significance threshold, as expected given the reduced statistical power associated with a smaller replicate sample size.

Two nutrient-tissue comparisons showed comparatively lower or less consistent correlation across confidence tiers: boron-deficient leaves and molybdenum-deficient leaves. For boron-deficient leaves, correlation with the full-replicate analysis was lowest across all genes (*r* = 0.74), and this instability was concentrated in a single replicate: exclusion of B_L_2 produced the lowest correlation among the four leave-one-out iterations for this comparison, indicating that this replicate had a disproportionate influence on the model fit relative to the other three boron-deficient leaf replicates. Molybdenum-deficient leaves showed a comparatively unusual pattern, with correlation declining rather than improving at the strictest confidence tier (*p*-adj < 0.001; *r* = 0.79), likely reflecting the small number of genes retained at this threshold (*n* = 15 genes) rather than a broader instability in the underlying signal. Both boron and molybdenum deficiency comparisons yielded a comparatively small number of significant differentially expressed genes overall (*n* = 14 each at log_2_FC ≥ 1 and *p*-adj < 0.05), and results for these two treatments should therefore be interpreted with appropriate caution relative to the more robust nutrient responses observed elsewhere in the dataset.

### 2.3. Transcriptome-Wide Responses to Nutrient Deficiency

**Figure 3:**
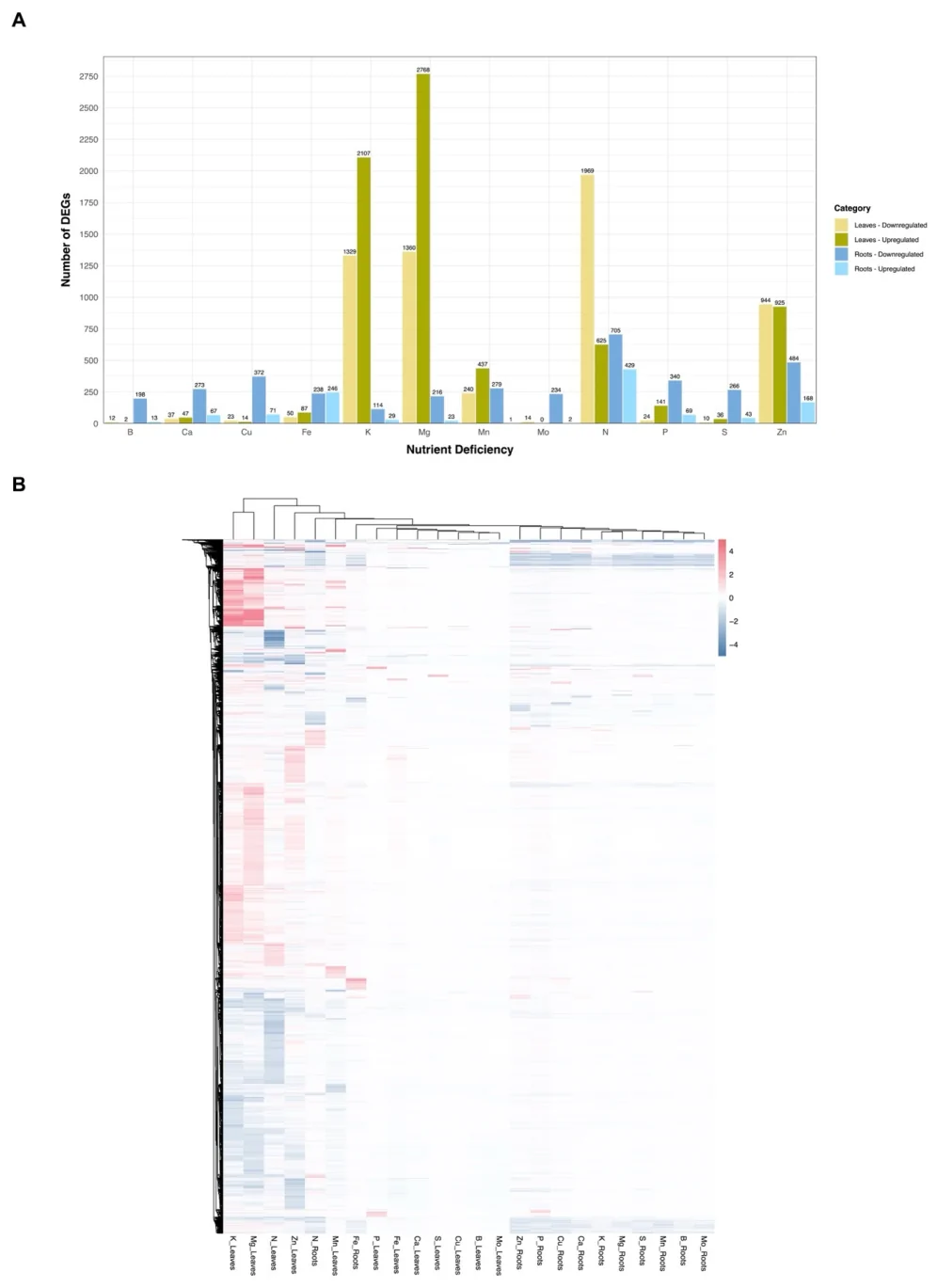
Differentially expressed genes (DEGs) were identified for each pairwise nutrient deficiency-versus-control comparison across both tissues. (**A**) Bar chart displaying DEGs grouped by tissue and direction of regulation, with leaves upregulated (green), leaves downregulated (yellow), roots upregulated (light blue), and roots downregulated (dark blue) for each treatment. (**B**) Heatmap showing log_2_FC values for all genes significant in at least one nutrient-versus-control comparison across twelve nutrient-deficiency treatments in leaf and root tissue. Red indicates upregulation and blue indicates downregulation relative to the respective tissue control; the color scale was capped at ±5 log_2_FC for visualization, with genes exceeding this threshold displayed at maximum color intensity. Genes (rows) and treatment comparisons (columns) were hierarchically clustered (Euclidean distance, average linkage) to group genes and conditions with similar expression patterns. DEGs were defined as genes with *p*-adj < 0.05 and absolute log_2_FC ≥ 1 from ashr-shrunken DESeq2 results.

The magnitude of transcriptional responses differed considerably among nutrient deficiencies. Volcano plots demonstrated that most differentially expressed genes exhibited moderate expression changes (generally between log_2_FC values of −10 and 10), with relatively few genes showing more extreme responses (Supplemental Figures S5 and S6). The abundance of significantly up- and downregulated genes varied substantially among nutrient deficiencies and tissues (Figure 3A), and these patterns were reflected in gene-level log_2_FC values across the combined set of significant differentially expressed genes (DEGs) (Figure 3B). Leaves exhibited substantially greater transcriptional plasticity than roots, particularly under nitrogen, potassium, magnesium, and zinc deficiency, whereas boron, copper, and molybdenum deficiencies produced comparatively few DEGs in leaf tissue. Root transcriptomes responded more uniformly across nutrient treatments, although nitrogen and zinc deficiency again elicited the largest responses. These findings demonstrate that nutrient limitation affected photosynthetically active tissues more strongly than nutrient-acquiring tissues, at least in terms of the overall number of genes affected.

**Figure 4:**
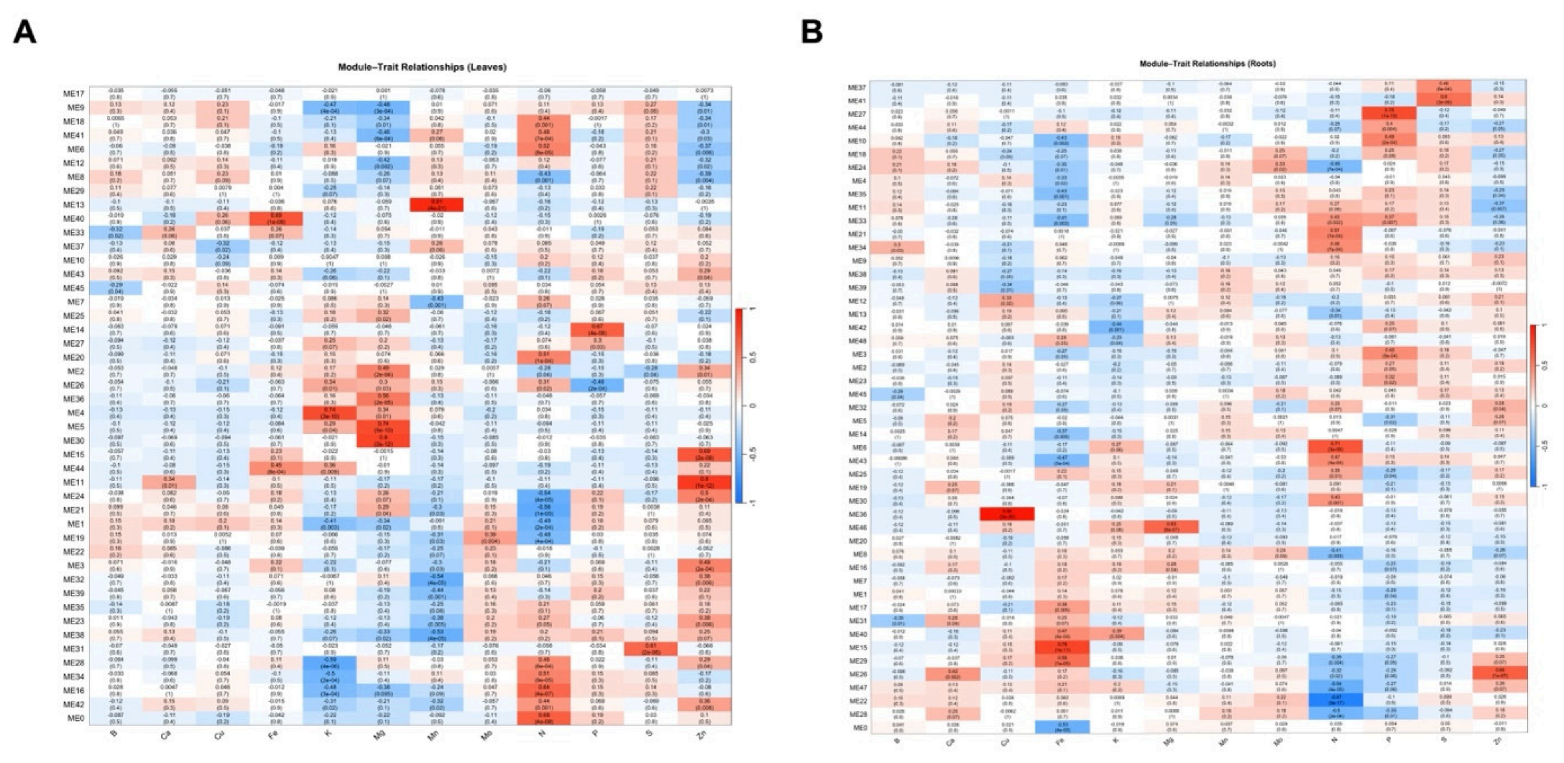
Signed module-trait relationship heatmap for (**A**) leaf and (**B**) root co-expression modules identified by WGCNA. Each cell displays the Pearson correlation coefficient between a module eigengene (ME, rows) and a nutrient deficiency trait (columns), with the associated *p*-value shown in parentheses below. The color scale ranges from blue (strong negative correlation, *r* = −1) to red (strong positive correlation, *r* = 1).

Co-expression network analysis identified 45 and 48 module eigengenes in leaf and root tissues, respectively (Figure 4A,B). Nutrient deficiencies producing the most extreme transcriptional responses generally exhibited the strongest positive module-trait associations (*r* ≥ 0.5, *p* < 0.05): nitrogen, phosphorus, sulfur, magnesium, iron, and zinc deficiency were each associated with multiple strongly correlated modules. Boron, calcium, and molybdenum deficiency exhibited comparatively weak transcriptional reprogramming across DEG and co-expression analyses. Copper deficiency was a notable exception to this overall pattern: despite producing relatively few DEGs, it was associated with the single strongest module-trait correlation observed in either tissue (root module eigengene 36 [ME36], *r* = 0.96), indicating a modest but highly coordinated transcriptional response. Significant negative module-trait correlations were also identified, most strongly between nitrogen deficiency and root ME22 (*r* = −0.87) and between potassium deficiency and leaf ME28 (*r* = −0.59), indicating that nitrogen and potassium deficiency each strongly suppressed distinct sets of co-expressed genes in addition to activating others.

These analyses demonstrate that nutrient deficiency responses differed substantially in both magnitude and tissue specificity. While several deficiencies produced extensive transcriptional reprogramming, others elicited relatively modest changes in gene number but nonetheless highly coordinated responses, as seen with copper deficiency, indicating that quinoa does not respond uniformly to nutrient limitation. Instead, transcriptomic responses varied with the physiological functions of the limiting nutrient and revealed potentially conserved stress programs shared across multiple nutrient deficiencies.

### 2.4. Shared Transcriptional Responses Across Nutrient Deficiencies

**Figure 5:**
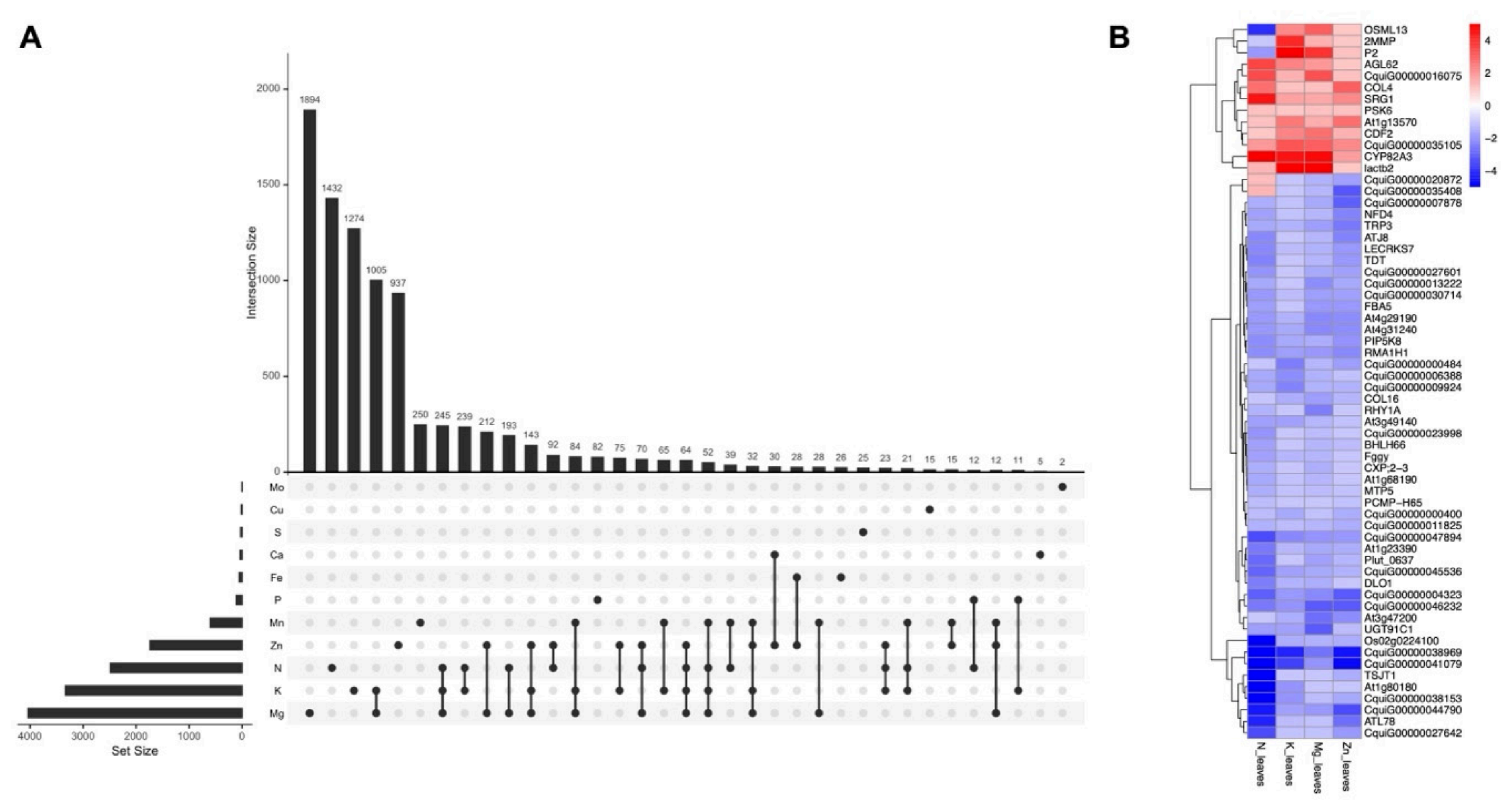
UpSet plot and heatmap of differentially expressed gene overlap across nutrient deficiency conditions in leaves. (**A**) UpSet plot showing the distribution and intersections of significant DEGs across all twelve nutrient deficiency treatments in leaves. Horizontal bars on the left indicate the total number of DEGs per treatment (set size), and vertical bars indicate the size of each intersection. Intersections with fewer than ten genes were excluded. Intersections are ordered by decreasing frequency. (**B**) Heatmap of log_2_FC values for DEGs present in the intersection common to nitrogen, potassium, magnesium, and zinc deficiency conditions in leaves. Rows represent individual genes and are hierarchically clustered, while columns represent treatments and are fixed in order. The color scale ranges from blue (log_2_FC = −4) to red (log_2_FC = 4), with white indicating no change. Gene names are shown on the right.

**Figure 6:**
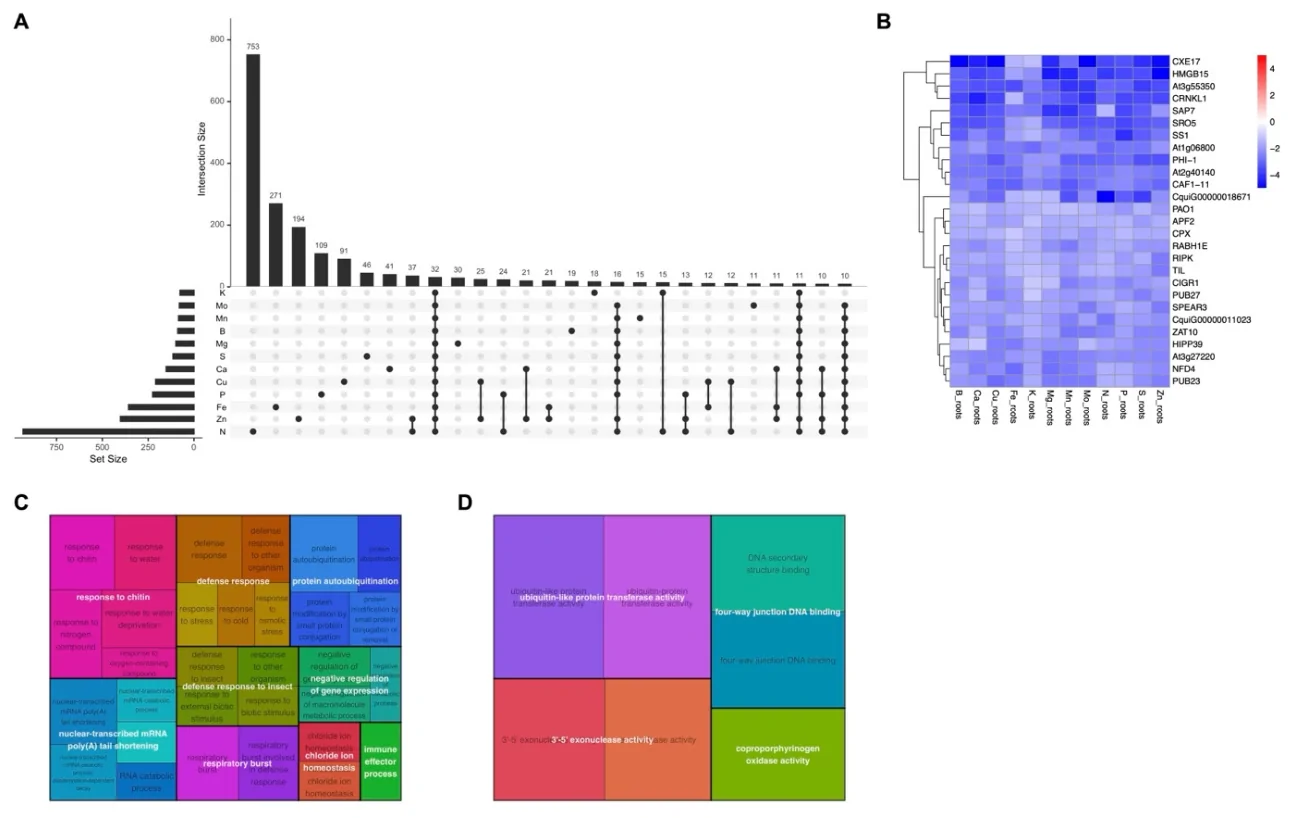
UpSet plot, heatmap, and functional enrichment of differentially expressed gene overlap across all nutrient deficiency conditions in roots. (**A**) UpSet plot showing the distribution and intersections of significant DEGs across all twelve nutrient deficiency treatments in roots. Horizontal bars on the left indicate the total number of DEGs per treatment (set size), and vertical bars indicate the size of each intersection. Intersections with fewer than ten genes were excluded. Intersections are ordered by decreasing frequency. (**B**) Heatmap of log_2_FC values for DEGs present in the intersection common to all twelve nutrient deficiency conditions in roots. Rows represent individual genes and are hierarchically clustered, while columns represent treatments. The color scale ranges from blue (log_2_FC = −4) to red (log_2_FC = 4), with white indicating no change. Gene names are shown on the right. (**C**) Treemap of enriched and semantically clustered Gene Ontology (GO) biological process (BP) terms for the shared root DEGs. (**D**) Treemap of enriched and semantically clustered molecular function (MF) Gene Ontology terms for the shared root DEGs. GO term enrichment was performed using TRAPID, and semantic clustering was performed using rrvgo.

UpSet analysis revealed substantial overlap among DEGs induced by different nutrient deficiencies (Figure 5 and Figure 6). Although each nutrient deficiency produced a distinct transcriptional signature, many responses were conserved across treatments, particularly among deficiencies in roots.

In leaves, nitrogen, potassium, magnesium, and zinc deficiency shared 64 differentially expressed genes (Figure 5A), which clustered into two broad expression patterns when visualized by log_2_FC: a consistently upregulated set and a larger consistently downregulated set (Figure 5B). Annotated genes among these shared DEGs included transcription factors involved in flowering and photoperiod regulation (e.g., *AGL62*, *COL4*, *COL16*, *CDF2*), stress- and hormone-signaling genes (e.g., *OSML13*, *PSK6*), and genes involved in protein turnover and secondary metabolism (e.g., *ATL78*, *DLO1*, *UGT91C1*). Functional enrichment of this shared gene set did not identify significantly enriched Gene Ontology (GO) terms, suggesting that although a core set of co-regulated genes is shared among these four deficiencies, their collective functions are too diverse to resolve as a coherent enriched pathway at this level of analysis. This may be compounded by limited functional annotation, as 23 of the 64 shared DEGs corresponded to hypothetical proteins lacking an assigned gene name or function.

In contrast, root transcriptomes exhibited a broadly conserved response shared across nearly all nutrient deficiencies (Figure 6A), with the 32 DEGs common to all twelve treatments consistently downregulated (Figure 6B). Annotated genes among these shared DEGs included *ZAT10*, *PUB23*, *PUB27*, and *HIPP39*, among others. Unlike the leaf-shared gene set, functional enrichment of these shared root DEGs identified numerous biological process terms, including response to chitin, defense response, response to water deprivation, and RNA catabolic process, as well as molecular function terms related to ubiquitin-like protein transferase activity and exonuclease activity (Figure 6C,D). This convergence of ubiquitin-mediated protein modification, mRNA turnover, and defense-response terms across genes shared by all twelve deficiencies points to a conserved root stress response that is largely independent of the specific nutrient being limited, suggesting that protein quality control, mRNA turnover, and immune priming represent core features of a generalized nutrient deficiency response in quinoa root tissue.

Compared with leaves, root responses were characterized by fewer nutrient-specific transcriptional changes and greater conservation across treatments, consistent with their primary role in nutrient acquisition and environmental sensing. Despite these conserved responses, each nutrient deficiency also activated distinct transcriptional programs consistent with the physiological functions of the limiting nutrient. Together, these findings demonstrate that nutrient stress in quinoa is characterized by two complementary regulatory strategies: a conserved transcriptional program that enables general adaptation to nutrient limitation, and nutrient-specific responses that attempt to maintain homeostasis under individual deficiencies. The following sections examine these nutrient-specific responses in greater detail.

### 2.5. Macronutrient Responses (N, P, K, S, Ca, and Mg)

The macronutrient deficiencies examined here varied substantially in the magnitude, tissue distribution, and regulatory coherence of their transcriptional responses. Nitrogen, potassium, and magnesium deficiencies produced the largest and most leaf-dominated transcriptomic responses, whereas phosphorus and sulfur deficiencies elicited more modest but tightly focused changes, and calcium deficiency produced the weakest and most diffuse signature of any macronutrient examined. Several deficiencies also differed in the extent to which their transcriptional responses were shared across tissues, ranging from strongly leaf-restricted programs to more broadly conserved responses involving overlapping regulatory networks in both leaves and roots. The following sections examine each macronutrient deficiency in turn, before proceeding to the micronutrient deficiencies in Section 2.6.

#### 2.5.1. Nitrogen Deficiency

##### Transcriptomic Response

Nitrogen deficiency elicited one of the strongest transcriptomic responses observed across all nutrient treatments, particularly in leaves, where 2594 differentially expressed genes were identified, including 1432 genes unique to this treatment (Supplemental Figure S7A). Root tissues exhibited a more moderate response, with 1134 DEGs, of which 753 were nitrogen-specific. GO term enrichment of the unique DEG sets revealed marked tissue-specific responses to nitrogen limitation: in leaves, condition-specific DEGs were enriched for photosynthesis, chloroplast organization, tetrapyrrole metabolism, amino acid metabolism, protein folding, and energy production (Supplemental Figure S7B,C), whereas in roots, condition-specific DEGs were enriched for nutrient transport, ion homeostasis, nitrogen recycling, and secondary metabolism (Supplemental Figure S7D,E). These results indicate that nitrogen limitation strongly suppresses photosynthetic metabolism while simultaneously promoting nutrient acquisition and recycling in roots.

**Figure 7:**
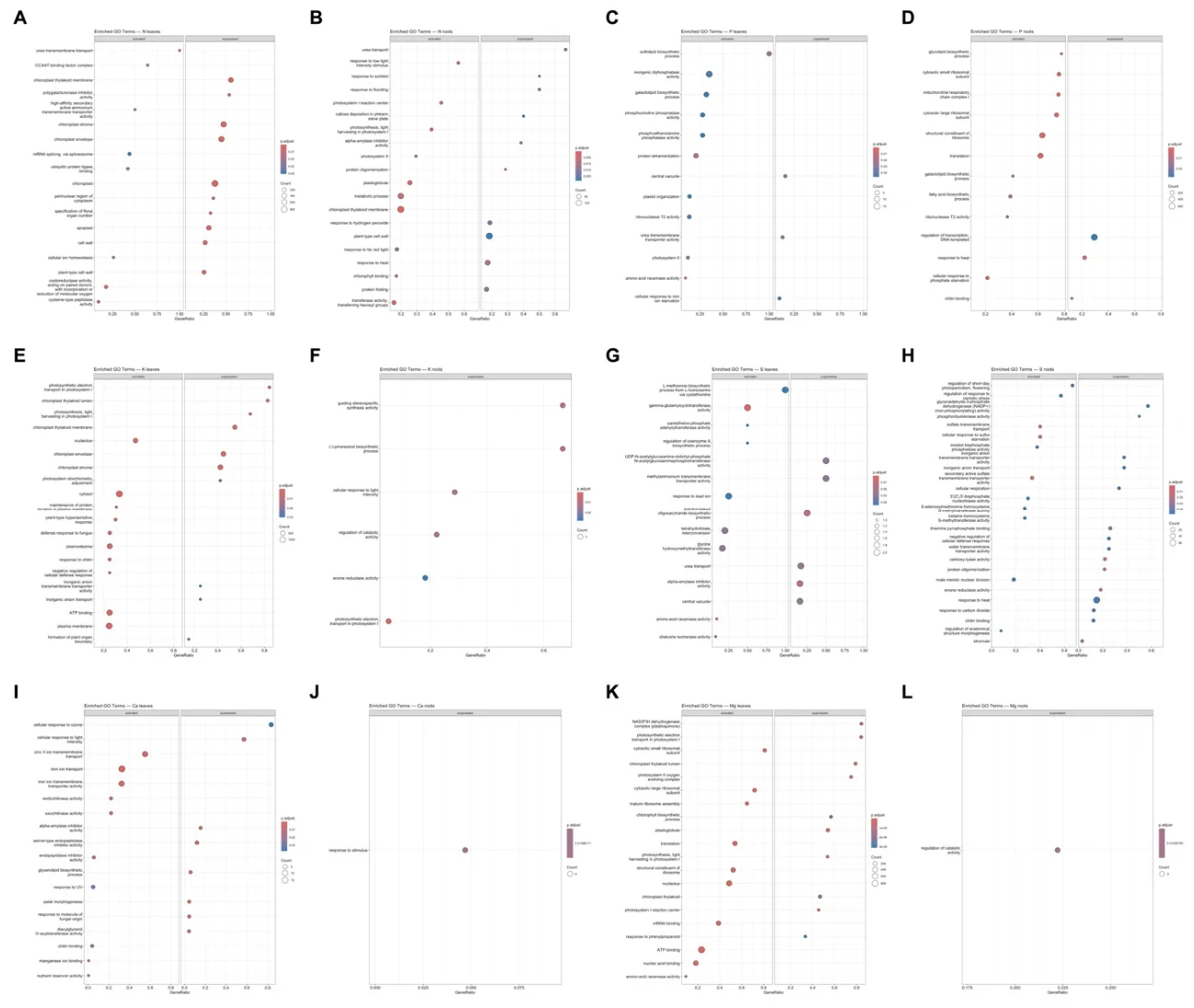
Gene set enrichment analysis (GSEA) of Gene Ontology (GO) terms for macronutrient deficiencies in leaf and root tissue. Dot plots of enriched GO terms for nitrogen ((**A**), leaf; (**B**), root), phosphorus ((**C**), leaf; (**D**), root), potassium ((**E**), leaf; (**F**), root), sulfur ((**G**), leaf; (**H**), root), calcium ((**I**), leaf; (**J**), root), and magnesium ((**K**), leaf; (**L**), root) deficiency. Each panel is faceted by direction of enrichment (activated and suppressed), with dot size proportional to the number of genes in each gene set and dot color indicating adjusted *p*-value. Up to the top 15 terms per direction are shown. GSEA was performed using the GSEA function in clusterProfiler with Benjamini–Hochberg correction and a *p*-value cutoff of 0.05.

Gene set enrichment analysis (GSEA) of the full ranked transcriptome reinforced and extended these tissue-specific patterns. In leaves, GO terms enriched among suppressed genes were associated with photosynthesis, chloroplast organization, allantoin transport, carbohydrate metabolism, and ion homeostasis, while enriched terms among activated genes included mRNA splicing, stomatal development, photoperiod regulation, and urea transport (Figure 7A; Supplemental Figure S8A,B). Consistent with this, enrichment of Kyoto Encyclopedia of Genes and Genomes (KEGG) pathways identified widespread suppression of photosynthesis, photosynthesis antenna proteins, nitrogen metabolism, ribosome function, and amino acid biosynthesis, alongside significant activation of autophagy, RNA polymerase, and spliceosome pathways (Supplemental Figure S8C,D) In roots, GSEA identified enrichment of terms associated with far-red light and heat responses, suberin biosynthesis, cuticle development, and phosphate homeostasis (Figure 7B; Supplemental Figure S8E,F), while KEGG pathway enrichment showed significant activation of photosynthesis and photosynthesis antenna protein pathways along with general metabolic pathways, with protein processing in the endoplasmic reticulum as the only significantly suppressed pathway (Supplemental Figure S8G,H). The activation of photosynthesis-associated genes in roots contrasts with their suppression in leaves, potentially reflecting a compensatory or tissue-specific transcriptional response to nitrogen limitation.

##### Candidate Genes and Regulatory Networks

Multivariate and co-expression network analyses identified several genes and regulatory modules strongly associated with nitrogen deficiency. In leaves, partial least squares discriminant analysis (PLS-DA) (component 1: 33% variance explained) highlighted genes involved in chloroplast function, protein turnover, and stress adaptation among the top variance in projection (VIP) -scoring transcripts, including *RPL2* (VIP = 2.06), *VHA-B2* (2.06), *TSB* (2.05), *PHYB* (2.05), and *GRXS9* (2.05) (Supplemental Figure S9A,B). GO term enrichment of transcripts with VIP scores ≥ 1.5 identified biological process terms associated with photosynthesis, plastid organization, protein targeting to chloroplasts, tetrapyrrole metabolism, and nitrogen compound transport, closely mirroring the patterns identified from the unique DEG analysis (Supplemental Figure S9C,D). Four module eigengenes were significantly correlated with nitrogen deficiency in leaves (ME16, *r* = 0.64; ME34, *r* = 0.51; ME20, *r* = 0.51; ME6, *r* = 0.52), of which only ME20 showed significant functional enrichment and was examined further (Figure 4A). Co-expression analysis identified module ME20 (*r* = 0.51, *p* = 1 × 10^−4^) as enriched for circadian regulation, glucan metabolism, and responses to abiotic stress (Supplemental Figure S10A,B). Hub genes within this module included *GIGANTEA* (*GI*), *TGA7*, *DNAJB6*, *SRK2I*, and *CYP89A9*, suggesting coordination between carbon metabolism, circadian regulation, and stress signaling during nitrogen limitation (Supplemental Figure S10C).

In roots, PLS-DA (component 1: 27% variance explained) identified several well-characterized regulators of nitrogen acquisition and utilization among the top VIP-scoring transcripts, including *NPF6.3* (VIP = 2.41), *NLP3* (2.38), and *DUR3* (2.38), along with genes involved in secondary metabolism and defense responses (Supplemental Figure S9E,F). GO term enrichment of root VIP transcripts identified biological process terms related to nitrogen compound responses, nutrient sensing, organic acid and anion transport, and allantoin and trehalose metabolism, in contrast to the chloroplast- and photosynthesis-dominated leaf response (Supplemental Figure S9G,H). WGCNA identified module ME6 (*r* = 0.71, *p* = 3 × 10^−9^) as highly correlated with nitrogen deficiency and enriched for suberin biosynthesis, fatty acid metabolism, phenylpropanoid metabolism, and cuticle development (Figure 4B; Supplemental Figure S10D,E). The identified hub genes, including *KCS2*, *KCS4*, *MYB93*, *CYP86A1*, and *RD22* suggest that nitrogen deficiency promotes structural remodeling of root barrier tissues and enhances nutrient-scavenging capacity (Supplemental Figure S10F).

##### Biological Interpretation

Across multiple analytical approaches, nitrogen deficiency consistently disrupted photosynthetic metabolism while activating pathways associated with nutrient recycling, transport, and stress adaptation. The coordinated downregulation of photosynthetic genes and the enrichment of autophagy and protein turnover pathways indicates that leaves reallocate resources away from growth and carbon fixation toward maintenance and nutrient conservation under nitrogen limitation, a well-documented response in plants [15].

In contrast, root transcriptomes emphasized altered nitrogen acquisition, remobilization, and structural adaptation. Repeated enrichment of nutrient transport and allantoin- and trehalose-related metabolism, along with the identification of *NPF6.3*, *NLP3*, and *DUR3*, suggests enhanced nitrogen scavenging and recycling under limiting conditions, consistent with a role for purine catabolism in nitrogen remobilization and recovery [16,17]. The identification of *NLP3* is particularly notable given the established roles of NLP-family transcription factors in regulating nitrogen use efficiency and nitrogen metabolism in both rice and quinoa [18,19]. The strong association of suberin biosynthesis with nitrogen-responsive co-expression modules further indicates that root barrier remodeling represents an important adaptive strategy for regulating nutrient and water uptake under nitrogen scarcity [20]. Notably, root transcriptomes showed activation, rather than suppression, of photosynthesis-related genes, in direct contrast to the pattern observed in leaves. This divergence may be consistent with the preferential allocation of resources toward root growth under nitrogen limitation, a pattern documented physiologically in other plant species as a shift in root-to-shoot biomass ratio [21], though the specific transcriptional basis for this pattern in quinoa roots warrants further investigation.

#### 2.5.2. Phosphorus Deficiency

##### Transcriptomic Response

Phosphorus deficiency produced a comparatively moderate transcriptional response in leaves and roots, with 165 and 409 differentially expressed genes (DEGs), respectively, including 82 and 109 genes unique to phosphorus deficiency (Supplemental Figure S11A). GO term enrichment of the unique DEG sets identified membrane lipid remodeling, sulfolipid biosynthesis, phosphatase activity, and cellular responses to phosphate starvation as dominant leaf-specific responses (Supplemental Figure S11B,C), while root-specific DEGs were enriched for phosphate transport and homeostasis, RNA degradation, nuclease activity, and lipid storage pathways (Supplemental Figure S11D,E), consistent with enhanced phosphate scavenging and internal remobilization under phosphorus limitation.

Gene set enrichment analysis of the full ranked transcriptome revealed additional, largely distinct patterns in each tissue. In leaves, GSEA identified relatively few enriched GO terms, associated with lipid organization, galactolipid biosynthesis, protein tetramerization, and cellular responses to iron starvation, along with suppression of the central vacuole term, potentially reflecting altered vacuolar phosphate storage and remobilization (Figure 7C; Supplemental Figure S12A,B). KEGG pathway enrichment identified activation of sulfoquinovose metabolism, vitamin B6 metabolism, and general metabolic pathways as significant in leaves (Supplemental Figure S12C,D). In roots, GSEA identified a substantially broader response, with enrichment of phosphate starvation responses, phospholipid catabolism, translation, and Golgi organization (Figure 7D; Supplemental Figure S12E,F). KEGG pathway enrichment showed activation of translation, DNA replication and repair, proteasome activity, oxidative phosphorylation, central carbon metabolism, and fatty acid biosynthesis, alongside suppression of spliceosome and endoplasmic reticulum protein processing pathways (Supplemental Figure S12G,H). Together, these findings indicate that phosphorus deficiency primarily promotes phosphate conservation and membrane remodeling in leaves, while roots undergo a broader transcriptional response spanning phosphate acquisition, translation, and energy metabolism.

##### Candidate Genes and Regulatory Networks

Multivariate and co-expression analyses identified several canonical regulators of phosphate starvation. In leaves, PLS-DA (component 1: 16% variance explained) identified *NLP7* (VIP = 2.86), *CML22* (2.82), *MKK3* (2.78), *WNK5* (2.79), and *PDIL5-2* (2.80) among the top VIP-scoring transcripts, reflecting altered regulation of nutrient signaling and cellular stress responses (Supplemental Figure S13A,B). GO term enrichment of transcripts with VIP scores ≥ 1.5 identified biological process terms associated with responses to oxygen-containing compounds and abiotic stimuli, energy metabolism, lipid metabolism, and ion homeostasis, indicating that phosphorus deficiency in leaves is characterized by metabolic reorganization and signaling responses associated with oxidative and nutrient stress (Supplemental Figure S13C,D). WGCNA identified module ME14 (*r* = 0.67, *p* = 4 × 10^−8^) as strongly associated with phosphorus deficiency and enriched for phosphate starvation responses, membrane lipid remodeling, and phosphate recycling (Figure 4A; Supplemental Figure S14A,B). Hub genes within this module included *SPX3*, *SQD2*, *MGD2*, *PAP2*, *PAP15*, *RNS2*, and *PLDZETA1*, all of which represent well-established components of phosphate starvation signaling (Supplemental Figure S14C).

In roots, PLS-DA (component 1: 33% variance explained) identified *MPT3* (VIP = 2.26), *PATL2* (2.26), *CSLA9* (2.26), *SEC1A* (2.26), and *BHLH86* (2.25) among the strongest discriminating genes (Supplemental Figure S13E,F). Notably, *SMO1-1* and an unannotated transcript, *CquiG00000030863*, ranked among the top VIP genes in both leaves and roots, suggesting these genes play a broader, tissue-independent role in the phosphorus deficiency response. GO term enrichment of root VIP transcripts identified biological process terms associated with root hair elongation, phosphate starvation responses, and arsenate transport, in contrast to the metabolic and oxidative stress responses that predominated in leaves, indicating that root transcriptomes were characterized by processes directly associated with phosphorus sensing and acquisition (Supplemental Figure S13G,H). WGCNA identified module ME27 (*r* = 0.75, *p* = 1 × 10^−10^), which shared many hub genes with the leaf network, including *SPX1*, *SQD2*, *MGD2*, *PAP2*, *PAP15*, *GDPD1*, and *RNS1* (Figure 4B; Supplemental Figure S14D–F). The extensive overlap between leaf and root co-expression networks along with the shared VIP genes identified by PLS-DA, suggests that phosphate starvation activates a highly conserved regulatory program across tissues.

##### Biological Interpretation

Phosphorus deficiency elicited one of the clearest examples of coordinated nutrient-specific regulation in quinoa. Across all analytical approaches, phosphate starvation consistently induced membrane lipid remodeling through replacement of phospholipids with galacto- and sulfolipids, increased phosphatase activity, and activation of phosphate recycling pathways. These responses are hallmarks of the canonical plant phosphate starvation response and reflect strategies that conserve intracellular phosphate while maintaining membrane integrity [22].

Although leaves and roots shared many core responses, roots exhibited substantially broader transcriptional reprogramming, including enhanced translation, oxidative phosphorylation, and phosphate acquisition pathways, consistent with the central role of roots in phosphate sensing, uptake, and nutrient foraging [23]. The conservation of major hub genes and discriminating VIP genes across both tissues, including *SPX1*, *SPX3*, *SQD2*, *MGD2*, *PAP2*, and *PAP15*, suggests that phosphate starvation is governed by a common regulatory network that coordinates phosphate sensing, remobilization, and homeostasis throughout the plant [22,24,25]. The enrichment of sulfoquinovose metabolism in both tissues further underscores the importance of sulfolipid biosynthesis as a phosphate-sparing mechanism under phosphorus deficiency [22,26]. Collectively, these findings indicate that quinoa responds to phosphorus limitation primarily through membrane lipid remodeling, phosphate conservation, and internal recycling, rather than extensive suppression of primary metabolism.

#### 2.5.3. Potassium Deficiency

##### Transcriptomic Response

Potassium deficiency elicited one of the largest transcriptional responses observed in leaf tissue, with 3436 differentially expressed genes (DEGs), including 1274 (37.1%) unique to this treatment (Supplemental Figure S15A). In contrast, roots exhibited comparatively few unique DEGs (18 of 143 total; 12.6%), indicating that most transcriptional responses were shared with other nutrient deficiencies (Supplemental Figure S15A). GO term enrichment of the unique DEG sets identified abiotic stress responses, osmotic regulation, lipid metabolism, signal transduction, and stomatal movement as the dominant biological processes in leaves (Supplemental Figure S15B,C); in roots, the small number of unique DEGs limited enrichment to only two significant terms, response to cycloheximide and response to ketone (Supplemental Figure S15D), indicating minimal root-specific functional signal.

Gene set enrichment analysis of the full ranked transcriptome revealed a substantially broader response in leaves than the unique DEG analysis alone suggested. GSEA-derived GO terms in leaves were enriched for systemic acquired resistance, plant-type hypersensitive response, photosystem I electron transport, oxidative stress response, and salicylic and jasmonic acid signaling (Figure 7E; Supplemental Figure S16A,B), while KEGG pathway enrichment identified broad suppression of photosynthesis and photosynthetic antenna proteins alongside activation of proteasome activity, ribosome biogenesis, MAPK signaling, glutathione metabolism, plant–pathogen interaction, and alpha-linolenic acid metabolism (Supplemental Figure S16C,D). In roots, GSEA identified a comparatively limited set of GO terms, all falling in the suppressed category and centered on photosystem I electron transport, light intensity response, and pinoresinol biosynthesis (Figure 7F; Supplemental Figure S16E,F), while betalain biosynthesis was the sole significantly enriched KEGG pathway, activated under potassium deficiency (Supplemental Figure S16G,H). Together, these results indicate that potassium deficiency primarily induces stress adaptation and defense responses while reducing photosynthetic activity in leaves, with roots showing a comparatively muted response.

##### Candidate Genes and Regulatory Networks

Multivariate and co-expression analyses identified numerous regulators associated with stress signaling, defense responses, and hormone metabolism. In leaves, PLS-DA (component 1: 36% variance explained) identified *LRK10L-1.2* (VIP = 1.94), *LRK10L-2.7* (1.93), *CKX3* (1.94), *WRKY40* (1.92), *PHT1-7* (1.93), *PLD1* (1.92), and *CDR1* (1.93) among the strongest discriminating genes (Supplemental Figure S17A,B). GO term enrichment of transcripts with VIP scores ≥ 1.5 identified biological process terms clustered around abiotic stimulus response, protein catabolic process, plant organ senescence, and defense and immune response, consistent with activation of stress perception, senescence, and cell death pathways under potassium limitation (Supplemental Figure S17C,D). Co-expression network analysis identified module ME4 (*r* = 0.74, *p* = 3 × 10^−10^), which was strongly associated with potassium deficiency and enriched for defense responses, programmed cell death, osmotic stress, protein phosphorylation, and sulfur amino acid metabolism (Figure 4A; Supplemental Figure S18A,B). Hub genes included *HSF24*, *GAD*, *CML41*, *GST*, *SNAP33*, *PDR1*, and *RD19B*, suggesting coordinated regulation of stress signaling, redox homeostasis, and vesicle trafficking (Supplemental Figure S18C).

Root transcriptomes exhibited comparatively weaker nutrient-specific network organization. PLS-DA (component 1: 36% variance explained) identified *TPP7* (VIP = 2.86), *ANNAT8* (2.77), *LBD1* (2.77), *GSTZ5* (2.74), and *IQD31* (2.71) among the strongest discriminating genes (Supplemental Figure S17E,F). GO term enrichment of root VIP transcripts identified biological process terms associated with defense response, fatty acid oxidation, osmotic stress, and secondary metabolite biosynthesis, contrasting with the broader abiotic stress and senescence signatures observed in leaves and suggesting a shift toward defense priming and oxidative stress management in roots (Supplemental Figure S17G,H). No co-expression module strongly correlated with potassium deficiency was identified in roots, consistent with the comparatively low number of root DEGs identified for this treatment.

##### Biological Interpretation

Potassium deficiency produced a transcriptional response distinct from those observed under nitrogen and phosphorus limitation. Rather than emphasizing nutrient recycling or phosphate conservation, potassium deficiency primarily activated pathways associated with osmotic stress, defense signaling, and oxidative stress while suppressing photosynthetic function, consistent with the well-established role of potassium in osmotic regulation, stomatal operation, and membrane integrity [27,28]. The strong enrichment of MAPK signaling, glutathione metabolism, and plant–pathogen interaction pathways suggests that potassium limitation is perceived as a broad cellular stress that triggers defense-related transcriptional programs. The suppression of photosynthesis and photosynthetic antenna proteins in leaves mirrors patterns observed under nitrogen deficiency, reflecting a general depression of photosynthetic function under macronutrient stress [29].

The limited root-specific response contrasts sharply with the extensive reprogramming observed in leaves and likely reflects the central physiological role of potassium in stomatal regulation, enzyme activation, and photosynthetic function, consistent with similar studies in quinoa [10,27]. The activation of betalain biosynthesis as the sole enriched KEGG pathway in roots suggests a modest reorientation of secondary metabolism rather than a substantial adaptive response to potassium limitation [30]. Together, these findings indicate that, unlike nitrogen and phosphorus deficiency, potassium limitation is met primarily with a leaf-centered stress and defense response rather than a nutrient acquisition or conservation strategy.

#### 2.5.4. Sulfur Deficiency

##### Transcriptomic Response

Sulfur deficiency elicited a comparatively modest transcriptomic response relative to nitrogen, phosphorus, and potassium deficiency, with 25 of 46 total leaf DEGs (54.3%) and 46 of 309 total root DEGs (14.9%) unique to this treatment (Supplemental Figure S19A). Despite the smaller overall number of DEGs, GO term enrichment of the unique DEG sets consistently highlighted sulfur assimilation, sulfur amino acid metabolism, one-carbon metabolism, sulfate transport, and methionine biosynthesis as the dominant biological processes affected by sulfur limitation in leaves (Supplemental Figure S19B,C). In roots, the unique DEG set showed broader enrichment of sulfur transport and sulfur starvation pathways, and tetrahydrofolate interconversion and S-adenosylmethionine metabolism, than in leaves (Supplemental Figure S19D,E).

Gene set enrichment analysis of the full ranked transcriptome reinforced and extended these findings. Leaf transcriptomes were characterized by activation of methionine biosynthesis, tetrahydrofolate interconversion, coenzyme A biosynthesis, and sulfur amino acid metabolism (Figure 7G; Supplemental Figure S20A,B), whereas root transcriptomes exhibited additional enrichment of sulfate transport, sulfur starvation responses, methyl-group metabolism, and osmotic stress regulation, alongside suppression of plastid carbon metabolism and respiration-associated genes (Figure 7H). No KEGG pathways reached significance in either tissue, consistent with a relatively targeted transcriptional response focused specifically on sulfur metabolism rather than broader metabolic reprogramming.

##### Candidate Genes and Regulatory Networks

Multivariate and co-expression analyses identified several well-characterized regulators of sulfur metabolism and sulfur starvation. In leaves, PLS-DA (component 1: 15% variance explained) identified *PED1* (VIP = 3.24), *ECT2* (3.16), *BGLU41* (3.14), *CYP59* (3.05), and *WEE1* (1.04) among the top VIP-scoring transcripts (Supplemental Figure S21A,B). GO term enrichment of transcripts with VIP scores ≥ 1.5 identified biological process terms associated with protein catabolic processes, plant organ senescence, and sulfur compound catabolism, suggesting that sulfur deficiency drives proteolytic recycling and cellular remodeling to mobilize sulfur-containing amino acids from existing proteins (Supplemental Figure S21C,D). Co-expression analysis identified module ME31 (*r* = 0.61, *p* = 2 × 10^−6^), enriched for sulfur compound metabolism, sulfate transport, glutathione catabolism, and one-carbon metabolism (Figure 4A; Supplemental Figure S22A,B). Hub genes within this module included *HMT1*, *SULTR4;2*, *MYB35*, *ST2*, and *SCPL21*, suggesting coordinated regulation of sulfur assimilation and recycling (Supplemental Figure S22C).

In roots, PLS-DA (component 1: 28% variance explained) identified *CNGC8* (VIP = 2.78), *DPMS1* (2.78), *TIF3F1* (2.77), *PXC2* (2.77), and *ALATS* (2.77) among the top VIP-scoring transcripts (Supplemental Figure S21E,F). Notably, *PED1* ranked among the top VIP genes in both leaves and roots, suggesting a conserved role for peroxisomal fatty acid metabolism in the sulfur deficiency response across tissues. GO term enrichment of root VIP transcripts identified biological process terms associated with cellular remodeling, sulfur and amino acid metabolism, and defense signaling, suggesting that sulfur-deficient roots undergo more extensive cellular remodeling than leaves (Supplemental Figure S21G,H). Module ME41 (*r* = 0.60, *p* = 3 × 10^−6^) represented a canonical sulfur starvation response characterized by strong enrichment of sulfate transport, sulfur assimilation, and methionine metabolism (Figure 4B; Supplemental Figure S22D,E). Hub genes, including *LSU2*, *SDI1*, *SULTR4;2*, *SHM6*, *LIP2*, and *BETV6*, are well-established markers of sulfur deficiency and indicate alteration of sulfur acquisition, transport, and remobilization pathways (Supplemental Figure S22F). The overlap of several hub genes between leaf and root networks, along with the shared VIP gene *PED1*, suggests that sulfur deficiency is regulated by a largely conserved transcriptional program across tissues.

##### Biological Interpretation

Sulfur deficiency produced one of the most metabolically focused transcriptomic responses observed in this study. Across all analytical approaches, sulfur limitation consistently activated pathways associated with sulfate transport, sulfur assimilation, methionine biosynthesis, and one-carbon metabolism, reflecting the central role of sulfur in amino acid biosynthesis and methyl-group transfer [31]. In contrast to nitrogen and potassium deficiencies, sulfur starvation did not induce widespread suppression of photosynthetic metabolism but instead promoted targeted reprogramming of sulfur acquisition and recycling pathways, consistent with the absence of any significant KEGG pathway enrichment in either tissue.

The repeated identification of *SULTR4;2*, *LSU2*, *SDI1*, and genes involved in methionine and glutathione metabolism indicates that sulfur homeostasis in quinoa is maintained through regulation of sulfur transport, internal remobilization, and one-carbon metabolism [32,33,34]. The presence of well-established sulfur deficiency marker genes such as *LSU2* and *SDI1* specifically in the root network, alongside the overlapping hub genes between leaf and root modules, suggests that while a core transcriptional program is shared across tissues, roots mount a more pronounced and specific sulfur starvation signaling response, consistent with their central role in sulfate acquisition and homeostasis under sulfur-limited conditions [33].

#### 2.5.5. Calcium Deficiency

##### Transcriptomic Response

Calcium deficiency elicited a comparatively limited transcriptional response relative to the other macronutrient deficiencies, with 84 and 309 total DEGs identified in leaves and roots, respectively, of which only 5 (6.0%) and 41 (13.3%) were unique to calcium deficiency (Supplemental Figure S23). This pattern indicates that the transcriptomic response to calcium deficiency was dominated by genes shared with other nutrient deficiencies rather than by a distinct calcium-specific response, and the small number of condition-specific DEGs in both tissues precluded meaningful GO term enrichment analysis of these unique gene sets.

Despite the absence of enrichment among condition-specific DEGs, GSEA of the full ranked leaf transcriptome identified GO terms enriched for ion transport, including iron and zinc transmembrane transport, UV response, and glycerolipid biosynthesis, alongside suppression of terms including petal morphogenesis and fungal response (Figure 7I; Supplemental Figure S24A,B). KEGG pathway enrichment identified phenylpropanoid biosynthesis as the sole significantly enriched pathway, which was suppressed (Supplemental Figure S24C,D). In roots, GSEA identified only a single significantly enriched GO term, response to stimulus, which was suppressed, and no KEGG pathways reached significance (Figure 7J). These findings indicate that calcium deficiency produced comparatively modest and diffuse transcriptomic reprogramming under the conditions examined, with oxidative stress emerging as a consistent theme across tissues despite the limited number of condition-specific genes.

##### Candidate Genes and Regulatory Networks

Multivariate analyses identified several genes associated with calcium signaling, ion transport, and oxidative stress. In leaves, PLS-DA (component 1: 20% variance explained) identified *OGDH* (VIP = 2.70), *LOX2.1* (2.70), *HMA2* (2.67), *FLOT1* (2.67), and *PSMD10* (2.70) among the top-discriminating genes (Supplemental Figure S25A,B). GO term enrichment of transcripts with VIP scores ≥ 1.5 identified biological process terms associated with metabolism, cellular transport, protein synthesis, stress responses, and chloroplast organization, including enrichment of iron ion transport and cellular response to superoxide, which may reflect secondary ionic imbalances and oxidative stress arising from impaired calcium signaling (Supplemental Figure S25C,D).

In roots, PLS-DA (component 1: 23% variance explained) highlighted *CML36* (VIP = 2.77), *NPF5.5* (2.82), *ARF8* (2.74), *MOT2* (2.73), and *UBP24* (2.78) among the top discriminating genes (Supplemental Figure S25E,F); notably, the presence of *CML36*, a calmodulin-like calcium sensor, is consistent with disruption of calcium-mediated signaling under calcium deficiency. GO term enrichment of root VIP transcripts identified biological process terms related to stress and defense responses, ion transport, oxidative stress, and protein trafficking, including response to reactive oxygen species (Supplemental Figure S25G,H). The recurrence of superoxide- and reactive-oxygen-species-related terms among the top discriminating genes in both leaves and roots suggests that oxidative stress is a consistent feature of the calcium deficiency response across tissues, potentially linked to the role of calcium in activating antioxidant defense pathways.

Unlike several other nutrient deficiencies, calcium deficiency was not associated with strongly correlated co-expression modules in either tissue, indicating that transcriptional responses were dispersed rather than organized into discrete regulatory networks.

##### Biological Interpretation

Calcium deficiency produced one of the weakest nutrient-specific transcriptional responses observed in this study. Rather than inducing extensive metabolic reprogramming, calcium limitation primarily altered genes associated with oxidative stress, ion transport, and cellular signaling [35]. The absence of strongly coordinated co-expression networks and the limited number of uniquely enriched pathways suggest that calcium deficiency largely activated components of a generalized nutrient stress response rather than a distinct calcium-specific transcriptional program.

The suppression of phenylpropanoid biosynthesis, the sole significantly enriched KEGG pathway identified in either tissue, is notable given the established role of calcium in cell wall structure and integrity and may reflect reduced investment in lignin biosynthesis and secondary cell wall reinforcement under calcium limitation [36]. The repeated enrichment of iron transport-related terms in both the top discriminating leaf genes and the leaf GSEA results further suggests a potential interaction between calcium and iron homeostasis, a relationship previously reported in Arabidopsis that warrants further investigation in quinoa [37]. These findings likely reflect the multiple structural and signaling roles of calcium in plant cells. Because calcium functions extensively as a second messenger, many physiological effects may be regulated through post-transcriptional signaling mechanisms that are not fully captured by steady-state transcript abundance [38], consistent with the comparatively modest transcriptional changes observed here despite the visible phenotypic symptoms in deficient plants.

#### 2.5.6. Magnesium Deficiency

##### Transcriptomic Response

Magnesium deficiency produced one of the strongest transcriptional responses observed in this study, particularly in leaves, where 4128 differentially expressed genes (DEGs) were identified, including 1894 (45.9%) unique to this treatment (Supplemental Figure S26A). GO term enrichment of the unique leaf DEG set consistently identified ribosome biogenesis, rRNA processing, translation, protein-RNA complex assembly, and RNA metabolism as the dominant biological processes altered by magnesium limitation (Supplemental Figure S26B,C). In contrast, root transcriptomes exhibited far fewer DEGs overall (239 total) and comparatively few nutrient-specific genes (30; 12.6%); the small number of unique root DEGs yielded only two enriched terms, both related to floral and inflorescence meristem identity, whose biological relevance to root-specific magnesium deficiency remains uncertain given the limited number of contributing genes (Supplemental Figure S26A,D).

Gene set enrichment analysis of the full ranked transcriptome reinforced and extended the leaf-specific pattern. GSEA-derived GO terms in leaves were enriched for ribosome biogenesis and translation, stress and defense signaling, ion transport, and developmental processes, while suppressed terms were dominated by photosynthesis, chlorophyll biosynthesis, and nonphotochemical quenching (Figure 7K; Supplemental Figure S27A,B). KEGG pathway enrichment similarly identified activation of ribosome, ribosome biogenesis, spliceosome, plant-pathogen interaction, and zeatin biosynthesis pathways, alongside suppression of photosynthesis and photosynthesis antenna proteins; suppression of the photosynthesis antenna protein pathway closely mirrored patterns observed under nitrogen, potassium, and manganese deficiency (Supplemental Figure S27C,D). In roots, GSEA identified only a single significantly enriched GO term, regulation of catalytic activity, which was suppressed, and no KEGG pathways reached significance (Figure 7L), consistent with the limited number of DEGs and indicating little evidence for a distinct magnesium-specific transcriptional program in roots. Collectively, these results indicate that magnesium deficiency primarily disrupts translational capacity while simultaneously suppressing photosynthetic function, with effects concentrated almost entirely in leaf tissue.

##### Candidate Genes and Regulatory Networks

Multivariate and co-expression analyses identified numerous regulators associated with ribosome assembly, RNA metabolism, and protein synthesis. In leaves, PLS-DA (component 1: 37% variance explained) identified *NMD3* (VIP = 2.03), *GLUD1* (2.03), *EXOSC1* (2.04), *WDR44* (2.05), *ABCC3* (2.04), *LACTB2* (2.04), and *SRO3* (2.03) among the top-discriminating genes (Supplemental Figure S28A,B). GO term enrichment of transcripts with VIP scores ≥ 1.5 identified biological process terms associated with ribosome biogenesis, RNA metabolism, protein synthesis, and cellular energy production, reflecting coordinated regulation of ribosome maturation, RNA processing, and cellular stress responses (Supplemental Figure S28C,D). Three module eigengenes were significantly correlated with magnesium deficiency in leaves (ME30, *r* = 0.80; ME5, *r* = 0.76; ME36, *r* = 0.56), of which ME30 showed the clearest functional coherence. Co-expression analysis identified ME30 (*r* = 0.80, *p* = 2 × 10^−12^) as enriched for mitochondrial function, transcriptional regulation, and anthocyanin biosynthesis (Figure 4A; Supplemental Figure S29A,B). Hub genes within ME30, including *CYP87A3*, *TIM23-3*, *IDM1*, *ASK11*, *TCTP*, and *AGL80*, suggest coordination among mitochondrial activity, transcriptional regulation, and stress adaptation during magnesium limitation (Supplemental Figure S29C).

In roots, PLS-DA (component 1: 16% variance explained) identified *MAIL3* (VIP = 3.08), *MLP31* (3.03), *PUB26* (3.01), *CHS* (3.00), and *PNSB5* (2.99) among the strongest discriminating genes (Supplemental Figure S28E,F). GO term enrichment of root VIP transcripts identified biological process terms associated with defense signaling, cell wall remodeling, and carbohydrate metabolism, in marked contrast to the translational and ribosomal signatures that dominated the leaf response, suggesting that leaves and roots prioritize fundamentally different adaptive strategies under magnesium limitation (Supplemental Figure S28G,H). Root transcriptomes exhibited weaker co-expression network organization overall, but identified module ME46 (*r* = 0.63, *p* = 5 × 10^−7^), which was enriched for glucosinolate biosynthesis, secondary metabolism, programmed cell death, and chlorophyll catabolite transport (Figure 4B; Supplemental Figure S29D,E). Hub genes including *SRO5*, *ABCC3*, and *UBICEP52-7* indicate activation of oxidative stress responses along with nutrient remobilization and senescence-associated pathways (Supplemental Figure S29F).

##### Biological Interpretation

Magnesium deficiency elicited a transcriptional response distinct from those of all other macronutrient deficiencies. Rather than primarily activating nutrient acquisition or conservation pathways, magnesium limitation strongly disrupted ribosome biogenesis, RNA processing, and protein synthesis while simultaneously suppressing photosynthesis and chlorophyll metabolism. These findings are consistent with the central structural role of magnesium in ribosome stability, RNA polymerase activity, and chlorophyll molecules [39,40,41].

The repeated enrichment of translational machinery across differential expression, pathway enrichment, and co-expression analyses suggests that impaired protein synthesis represents one of the earliest and most prominent consequences of magnesium deficiency in quinoa. The accompanying suppression of photosynthetic pathways further indicates that reduced translational capacity contributes directly to declining photosynthetic performance under magnesium limitation. The leaf co-expression module ME30 additionally highlighted enrichment of mitochondrial protein import and anthocyanin biosynthesis, both of which have established links to magnesium availability: magnesium is required for mitochondrial function and ATP synthesis, and anthocyanin accumulation is itself a well-documented visual symptom of magnesium deficiency in plants [42,43], suggesting that ME30 captures coordinated metabolic and regulatory adjustments to magnesium limitation beyond translation alone.

Together, these findings indicate that disruption of ribosome biogenesis and translational capacity represents a central and early consequence of magnesium deficiency in quinoa, with cascading effects on photosynthetic performance and broader mitochondrial and pigment-related metabolism.

### 2.6. Micronutrient Responses (Fe, Zn, Mn, B, Cu, Mo)

Micronutrient deficiencies varied widely in the magnitude and tissue distribution of their transcriptional responses. In contrast to the macronutrients, which primarily affected central metabolic processes such as photosynthesis, translation, and nutrient assimilation, micronutrient deficiencies more often altered pathways associated with metal homeostasis, oxidative stress, and specialized metabolic or structural functions reflecting the specific biochemical roles of each element. As with the macronutrients, the number of differentially expressed genes did not always correspond directly to the degree of transcriptional coordination observed. Although several micronutrients shared common stress responses, each deficiency also activated distinct transcriptional programs reflecting the biochemical roles of the limiting element. The following sections describe these micronutrient deficiency-specific responses.

#### 2.6.1. Zinc Deficiency

##### Transcriptomic Response

Zinc deficiency elicited one of the strongest transcriptomic responses among the micronutrients, with 1869 and 652 differentially expressed genes (DEGs) identified in leaves and roots, respectively, including 937 (50.1%) and 194 (29.8%) unique to this treatment (Supplemental Figure S30A). GO term enrichment of the unique DEG sets identified extensive regulation of cell cycle progression, DNA replication, chromosome organization, and stress responses in leaves (Supplemental Figure S30B,C), whereas roots exhibited enrichment of metal ion homeostasis, redox metabolism, and transition metal transport (Supplemental Figure S30D,E). These findings indicate that zinc deficiency affects both genome maintenance and metal homeostasis while producing stronger and more extensive transcriptional responses in leaves than in roots.

**Figure 8:**
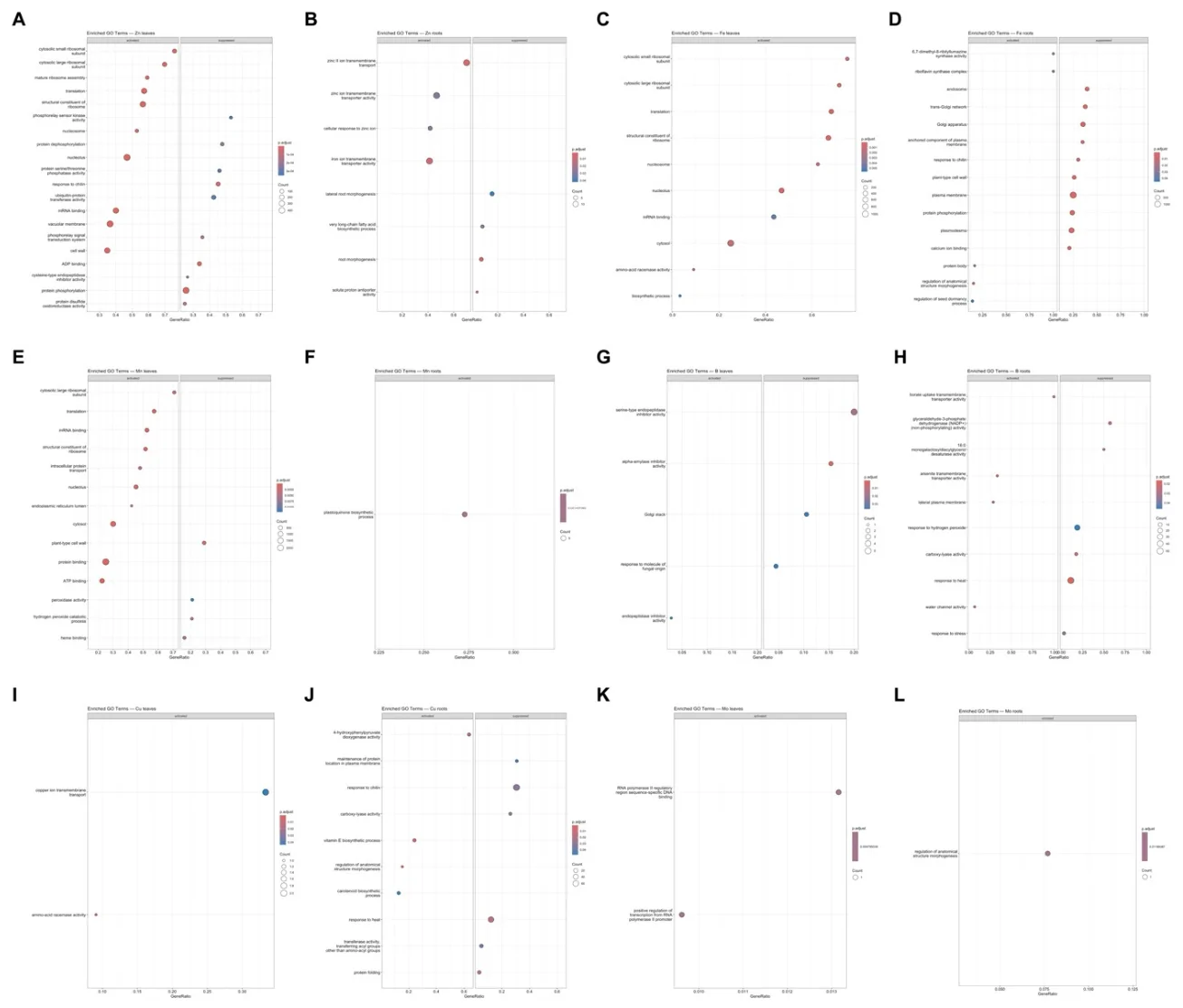
Gene set enrichment analysis (GSEA) of Gene Ontology (GO) terms for micronutrient deficiencies in leaf and root tissue. Dot plots of enriched GO terms for zinc ((**A**), leaf; (**B**), root), iron ((**C**), leaf; (**D**), root), manganese ((**E**), leaf; (**F**), root), boron ((**G**), leaf; (**H**), root), copper ((**I**), leaf; (**J**), root), and molybdenum ((**K**), leaf; (**L**), root) deficiency. Each panel is faceted by direction of enrichment (activated and suppressed), with dot size proportional to the number of genes in each gene set and dot color indicating adjusted *p*-value. Up to the top 15 terms per direction are shown. GSEA was performed using the GSEA function in clusterProfiler with Benjamini–Hochberg correction and a *p*-value cutoff of 0.05.

Gene set enrichment analysis of the full ranked transcriptome reinforced and extended these tissue-specific patterns. In leaves, GSEA-derived GO terms were enriched for ribosome biogenesis and translation, light and UV-related stress responses, miRNA-mediated gene silencing, iron ion transport, and cell wall organization (Figure 8A; Supplemental Figure S31A,B), while KEGG pathway enrichment identified activation of DNA replication, mismatch repair, homologous recombination, base excision repair, ribosome biogenesis, and oxidative phosphorylation, along with suppression of photosynthesis and autophagy (Supplemental Figure S31C,D). In roots, GSEA identified enrichment of zinc ion transmembrane transport and root and lateral root morphogenesis (Figure 8B; Supplemental Figure S31E,F), while KEGG pathway enrichment identified activation of DNA replication and motor protein pathways as the only significantly enriched pathways (Supplemental Figure S31G,H). Collectively, these results indicate that zinc deficiency activates extensive transcriptional programs associated with genome stability and cellular maintenance, with roots showing a more focused response centered on zinc acquisition and root developmental processes.

##### Candidate Genes and Regulatory Networks

In leaves, PLS-DA (component 1: 34% variance explained) identified *ZIP1* (VIP = 1.96), *ARP8* (1.97), *ADF4* (1.96), *DRM1* (1.96), *APX1* (1.96), *MSH1* (1.96), *GLIP7* (1.96), and *UBA2A* (1.96) among the strongest discriminating genes (Supplemental Figure S32A,B). GO term enrichment of transcripts with VIP scores ≥ 1.5 identified biological process terms associated with ribosome biogenesis, translation, cell cycle progression, and DNA replication, paralleling the cell cycle and DNA replication signatures identified in the unique DEG analysis and reflecting coordinated regulation of DNA maintenance, chromatin remodeling, and oxidative stress (Supplemental Figure S32C,D).

In roots, PLS-DA (component 1: 26% variance explained) identified *ZIP1* (VIP = 2.43), along with *RMA1* (2.46), *FSD2* (2.43), and three additional superoxide dismutase isoforms (*SODCC* (2.47), *SODCC.2* (2.45), *SODCP* (2.44)), among the top discriminating genes (Supplemental Figure S32E,F), a pattern consistent with zinc’s role as a cofactor for Cu/Zn superoxide dismutase and suggesting direct disruption of antioxidant capacity under zinc limitation. GO term enrichment of root VIP transcripts identified biological process terms associated with metal ion homeostasis, oxidative stress responses, cell cycle regulation, and DNA replication, indicating that zinc deficiency disrupts cell proliferation in roots as well as leaves (Supplemental Figure S32G,H). *ZIP1* ranked among the top VIP genes in both tissues, suggesting a conserved role for zinc transport across leaf and root responses.

Co-expression analyses further supported these responses. Two module eigengenes were significantly correlated with zinc deficiency in leaves (ME11, *r* = 0.80, *p* = 1 × 10^−12^; ME15, *r* = 0.69, *p* = 2 × 10^−8^), of which ME11 showed the clearer functional signature (Figure 4A; Supplemental Figure S33A,B). Leaf module ME11 and root module ME26 (*r* = 0.66, *p* = 1 × 10^−7^) exhibited substantial overlap in both hub genes and enriched biological processes, including zinc transport, transition metal homeostasis, reactive oxygen species detoxification, and oxidative stress responses (Figure 4A,B; Supplemental Figure S33D,E). Shared hub genes, including *ZIP1*, *ZIP5*, *HMA1*, and *CYP86B1*, indicate that zinc deficiency activates a highly conserved regulatory network responsible for maintaining metal homeostasis across tissues (Supplemental Figure S33C,F).

##### Biological Interpretation

Among all micronutrient deficiencies examined, zinc deficiency produced one of the most comprehensive transcriptional responses. Zinc limitation strongly affected DNA replication, chromosome maintenance, and cell cycle regulation, consistent with the essential structural role of zinc in numerous DNA-binding proteins, zinc-finger transcription factors, and DNA repair enzymes [44,45].

The repeated enrichment of zinc transporters, DNA repair pathways, and oxidative stress responses across multiple analytical approaches suggests that zinc deficiency simultaneously compromises genome stability and cellular redox homeostasis. The presence of multiple superoxide dismutase isoforms among the top root discriminating genes is particularly notable, as zinc serves as a cofactor for Cu/Zn-superoxide dismutase, and their differential expression likely reflects direct disruption of antioxidant capacity under zinc limitation [46]. The extensive overlap between leaf and root regulatory networks, anchored by shared hub genes including *ZIP1*, *ZIP5*, *HMA1*, and *CYP86B1*, further indicates that maintenance of intracellular zinc homeostasis is coordinated by a conserved transcriptional program operating across multiple tissues, centered on metal-ion acquisition and management of associated oxidative stress [47,48].

#### 2.6.2. Iron Deficiency

##### Transcriptomic Response

Iron deficiency generated one of the most distinctive micronutrient-specific transcriptomic signatures among the micronutrients, with 137 and 484 total DEGs identified in leaves and roots, respectively, of which 26 (19.0%) and 271 (56.0%) were unique to this treatment (Supplemental Figure S34A). Notably, iron deficiency exhibited the opposite tissue pattern from most other nutrients examined: rather than leaves contributing the larger share of condition-specific DEGs, roots accounted for the majority of iron-specific transcriptional changes, consistent with their central role in iron acquisition from the rhizosphere. GO term enrichment of the unique DEG sets showed that leaf transcriptomes primarily regulated iron transport, transition metal homeostasis, vascular redistribution, and responses to iron availability (Supplemental Figure S34B,C), whereas root transcriptomes exhibited broader activation of iron acquisition, flavin biosynthesis, amino acid metabolism, hormone signaling, and stress-response pathways (Supplemental Figure S34D,E).

Gene set enrichment analysis of the full ranked transcriptome reinforced these tissue-specific patterns while also revealing a cross-nutrient parallel. In leaves, GSEA-derived GO terms were enriched for translation and cellular responses to iron starvation (Figure 8C; Supplemental Figure S35A,B), while KEGG pathway enrichment identified activation of multiple DNA repair pathways, including mismatch repair, base excision repair, and homologous recombination, alongside ribosome biogenesis and oxidative phosphorylation, closely paralleling the DNA repair signatures previously identified under zinc deficiency (Supplemental Figure S35C,D). In roots, GSEA-derived GO terms were enriched for cell wall remodeling, actin cytoskeleton organization, arsenate transport, and responses to chitin and heat (Figure 8D; Supplemental Figure S35E,F), while KEGG pathway enrichment identified riboflavin metabolism as the sole significantly activated pathway, with broad upregulation across the riboflavin biosynthetic route, alongside suppression of plant hormone signal transduction and plant–pathogen interaction pathways (Supplemental Figure S35G,H), consistent with structural adaptations associated with the Strategy I iron deficiency response.

##### Candidate Genes and Regulatory Networks

In leaves, PLS-DA (component 1: 26% variance explained) identified genes involved in ribosome biogenesis, DNA replication, and protein turnover among the strongest discriminators of iron deficiency, including *RFA1* (VIP = 2.44), *CHX15* (2.51), *BPC6* (2.47), and *ARG7* (2.40) (Supplemental Figure S36A,B). GO term enrichment of transcripts with VIP scores ≥ 1.5 identified biological process terms associated with ribosome biogenesis, translation, DNA replication, protein turnover, and cell cycle regulation, sharing features with the responses observed under magnesium and zinc deficiency and potentially reflecting a broader disruption of iron-dependent processes supporting genome maintenance and protein synthesis (Supplemental Figure S36C,D). Co-expression analysis identified module ME40 (*r* = 0.69, *p* = 1 × 10^−8^) as strongly associated with iron deficiency in leaves, with hub genes including *BTS* (*BRUTUS*), *BHLH47*, *OPT3*, and *NRAMP3*, forming a core iron-sensing and signaling network consistent with systemic iron homeostasis regulation described in other plant species (Figure 4A; Supplemental Figure S37A–C).

In roots, PLS-DA (component 1: 18% variance explained) identified *NRAMP5* (VIP = 2.77), *NAAT1* (2.79), *RIBA1* (2.81), *NPF5.8* (2.77), and *LBD1* (2.88) as prominent discriminating genes associated with iron transport, nicotianamine metabolism, and flavin biosynthesis (Supplemental Figure S36E,F). GO term enrichment of root VIP transcripts identified biological process terms associated with metal ion homeostasis, cell wall remodeling, and trichoblast differentiation, consistent with the well-documented Strategy I iron deficiency response involving root hair proliferation and upregulation of iron acquisition machinery (Supplemental Figure S36G,H). Two module eigengenes were significantly correlated with iron deficiency in roots (ME15, *r* = 0.78; ME29, *r* = 0.56), of which ME15 showed the clearer functional signature, with enrichment for flavin and riboflavin biosynthesis, hormone signaling, and iron acquisition processes (Figure 4B; Supplemental Figure S37D,E). Hub genes within ME15, including *NAAT1* and *RIBA1*, reinforce the repeated identification of riboflavin metabolism as a core component of the root iron acquisition response across independent analyses (Supplemental Figure S37F).

##### Biological Interpretation

Iron deficiency produced one of the most specialized transcriptomic responses observed in this study, with clearly complementary strategies in leaves and roots. In leaves, the coordinated activation of ribosome biogenesis alongside multiple DNA repair pathways parallels patterns observed under zinc and magnesium deficiencies, suggesting that disruption of iron-dependent metalloprotein function may impose a genome-maintenance burden shared across several micronutrient deficiencies. The leaf-specific co-expression module ME40, anchored by the iron-sensing regulators *BTS*, *BHLH47*, *OPT3*, and *NRAMP3*, further indicates that leaves participate directly in systemic iron sensing and redistribution rather than serving solely as a site of deficiency symptoms.

In roots, the repeated enrichment of riboflavin biosynthesis across the DEG, PLS-DA, WGCNA, and GSEA/KEGG analyses was unique among all nutrient deficiencies examined. Riboflavin and its derivatives are known to accumulate and be secreted by iron-deficient roots, facilitating ferric iron reduction in the rhizosphere prior to uptake during Strategy I iron acquisition [49,50]. The consistency of this signal across four independent analytical approaches provides strong evidence that flavin metabolism represents a central, specific component of the quinoa iron acquisition response, distinct from the broader stress and metabolic reprogramming observed under other nutrient deficiencies.

Together, these findings indicate that iron deficiency elicits complementary tissue-specific responses in quinoa: leaves engage in iron sensing, genome maintenance, and systemic redistribution, while roots activate a specialized riboflavin-centered acquisition strategy consistent with Strategy I iron uptake mechanisms.

#### 2.6.3. Manganese Deficiency

##### Transcriptomic Response

Manganese deficiency produced 677 and 280 total DEGs in leaves and roots, respectively, of which 250 (36.9%) and 15 (5.4%) were unique to this treatment (Supplemental Figure S38A), indicating a substantially stronger and more distinctive transcriptional response in leaves. GO term enrichment of the unique leaf DEG set identified endoplasmic reticulum (ER) stress, the unfolded protein response, protein folding and quality control, and cell wall metabolism as dominant biological processes (Supplemental Figure S38B,C), consistent with disruption of ER-resident glycoprotein processing, several components of which require manganese as a cofactor. In roots, the small number of unique DEGs limited enrichment analysis, though the available signal centered on hypoxia response and oxidative stress (Supplemental Figure S38D), consistent with the role of Mn-superoxide dismutase in root antioxidant defense.

Gene set enrichment analysis of the full ranked transcriptome reinforced and extended the leaf-specific pattern. In leaves, GSEA-derived GO terms were enriched for protein homeostasis, intracellular protein trafficking, translation, and oxidative stress responses, including hydrogen peroxide catabolism (Figure 8E; Supplemental Figure S39A,B), while KEGG pathway enrichment identified activation of ribosome biogenesis, proteasome activity, spliceosome function, and ER protein processing, along with suppression of photosynthesis and photosynthesis antenna proteins, consistent with the essential role of manganese in the oxygen-evolving complex of photosystem II (Supplemental Figure S39C,D). In roots, GSEA identified plastoquinone biosynthesis as the sole significantly enriched GO term, with no KEGG pathways reaching significance, indicating a very limited but biologically relevant condition-specific signal in root tissue (Figure 8F).

##### Candidate Genes and Regulatory Networks

In leaves, PLS-DA (component 1: 26% variance explained) identified *ABCC4* (VIP = 2.35), *NAC078* (2.34), *NAC082* (2.34), *CYP72A219* (2.34), and *SEC11C* (2.33) among the strongest discriminating genes (Supplemental Figure S40A,B). GO term enrichment of transcripts with VIP scores ≥ 1.5 identified biological process terms associated with photosynthesis, ER stress, carbohydrate and lipid metabolism, and hormone-mediated regulation, including enrichment of photosystem I electron transport, likely reflecting compensatory rebalancing of photosynthetic electron flow when photosystem II activity is compromised by manganese limitation (Supplemental Figure S40C,D). Co-expression analysis identified module ME13 (*r* = 0.91, *p* = 4 × 10^−21^) as strongly associated with manganese deficiency in leaves—the second-strongest single module-trait correlation observed across all nutrient deficiencies examined in this study (Figure 4A). Hub genes within ME13, including *HSP70-17*, *PDIL2-3*, *SEC61G2*, *SEC61A*, *SEC31B*, and *SAR1A*, span ER protein translocation, COPII vesicle formation, and chaperone function, reinforcing disruption of secretory pathway processing as a central feature of the manganese deficiency response in leaves (Supplemental Figure S41A–C).

In roots, PLS-DA (component 1: 22% variance explained) identified *PAM71-HL* (VIP = 2.89), a manganese transporter directly involved in loading manganese into the thylakoid lumen for photosystem II activity, among the top discriminating genes, along with *CML36* (2.87), *ZAT10* (2.86), and *GRXC9* (2.86) (Supplemental Figure S40E,F). GO term enrichment of root VIP transcripts identified biological process terms associated with stress and defense responses, protein turnover, and cell wall remodeling, indicating that manganese-deficient roots are simultaneously responding to oxidative and abiotic stress while activating mechanisms associated with manganese homeostasis (Supplemental Figure S40G,H). No root co-expression modules met the threshold for strong correlation with manganese deficiency, indicating substantially weaker network organization than in leaves.

##### Biological Interpretation

Manganese deficiency elicited a transcriptomic response in leaves centered on two complementary themes: disruption of ER-resident glycoprotein processing and suppression of photosynthetic function. The exceptionally strong correlation between ME13 and manganese deficiency, supported by hub genes spanning ER translocation, vesicle formation, and chaperone activity, points to impaired manganese-dependent ER glycosyltransferase function as a central and previously underappreciated consequence of manganese limitation in quinoa [51,52,53]. In parallel, suppression of photosynthesis and photosynthesis antenna protein pathways, along with enrichment of photosystem I electron transport among leaf VIP genes, reflects the essential role of manganese in the oxygen-evolving complex of photosystem II [54].

In roots, the identification of *PAM71-HL* among the top discriminating genes is particularly notable given its direct, established role in manganese transport into the thylakoid [55], even though the broader root transcriptome showed comparatively limited and diffusely organized responses to manganese deficiency. Together, these findings indicate that manganese deficiency primarily affects leaf tissue through the combined disruption of secretory pathway function and photosynthetic capacity, whereas roots mount a more limited response centered on oxidative stress and the manganese transport machinery.

#### 2.6.4. Boron Deficiency

##### Transcriptomic Response

Boron deficiency produced one of the weakest and most asymmetric transcriptomic responses observed among all nutrients examined. In leaves, only 14 total DEGs were identified, none of which were unique to boron deficiency, precluding leaf-specific functional enrichment analysis (Supplemental Figure S42A). In roots, 211 total DEGs were identified, of which 19 (9.0%) were unique to this treatment; despite this small number, GO term enrichment of the unique DEG set identified a specific and coherent signal centered on borate transport and homeostasis, including enrichment of borate and arsenite transmembrane transporter activity, reflecting the well-documented overlap in substrate specificity between boron and arsenite transport via NIP-family aquaporins (Supplemental Figure S42A–C).

Gene set enrichment analysis of the full ranked transcriptome reinforced this tissue asymmetry. In leaves, GSEA identified very few significant GO terms, most notably suppression of the Golgi stack cellular component, consistent with the established role of boron in Golgi-dependent cross-linking of rhamnogalacturonan II during cell wall assembly (Figure 8G; Supplemental Figure S43A); no KEGG pathways reached significance in leaves. In roots, GSEA identified activation of borate uptake transporter activity as the most strongly enriched GO term, alongside stress-related terms including responses to heat and hydrogen peroxide (Figure 8H; Supplemental Figure S43B,C), while KEGG pathway enrichment identified protein processing in the endoplasmic reticulum as the sole significantly suppressed pathway, paralleling the Golgi-related disruption observed in leaves and suggesting broader impairment of the secretory pathway under boron limitation (Supplemental Figure S43D,E).

##### Candidate Genes and Regulatory Networks

In leaves, PLS-DA (component 1: 17% variance explained; component 2: 24%) identified *RPS28* (VIP = 3.12), *TIM23-3* (3.05), *CRT3* (2.93), and *HCBT1* (2.99) among the strongest discriminating genes (Supplemental Figure S44A,B). GO term enrichment of transcripts with VIP scores ≥ 1.5 identified biological process terms associated with cell cycle progression, chromosome segregation, and intracellular trafficking, along with molecular function terms related to ER protein folding, consistent with boron’s established role in cell wall integrity and cell plate formation during cytokinesis, where boron cross-links rhamnogalacturonan II pectin chains to stabilize the expanding cell wall [56] (Supplemental Figure S44C,D).

In roots, PLS-DA (component 1: 21% variance explained) identified *UGT76B1* (VIP = 3.06), *MDAR4* (3.00), and *PRT6* (2.92) among the top-discriminating genes (Supplemental Figure S44E,F). GO term enrichment of root VIP transcripts identified biological process terms associated with stress and defense responses, cell wall organization, and ion transport, with recurrent enrichment of arsenite transport terms reinforcing the co-regulation of boron and arsenite transport identified in the unique DEG analysis, likely through shared NIP-family aquaporin transporters (Supplemental Figure S44G,H).

Neither tissue yielded co-expression modules with a strong positive correlation to boron deficiency, and boron-responsive genes appeared distributed across multiple expression patterns rather than concentrated within coordinated modules, consistent with the low number of condition-specific DEGs identified in both tissues.

##### Biological Interpretation

Boron deficiency elicited a markedly asymmetric transcriptional response: leaves showed almost no evidence of a distinct boron-specific signature, while roots mounted a small but highly targeted response centered on boron acquisition. The repeated identification of borate transport, water channel activity, and arsenite co-transport across the DEG, PLS-DA, and GSEA analyses in roots implicates NIP-family aquaporins as central components of the root boron deficiency response, with the recurrent arsenite signal reflecting shared transporter substrate specificity rather than an independent arsenic-related response [57,58,59].

The suppression of Golgi-associated functions in leaves and ER protein processing in roots further suggests disruption of the secretory pathway under boron limitation, consistent with boron’s requirement for the synthesis and export of cell wall components [60]. Together, these findings suggest that boron’s physiological effects may act more through direct structural disruption of existing cell wall and membrane components than through large-scale transcriptional reprogramming, despite the visible phenotypic symptoms of deficiency.

#### 2.6.5. Copper Deficiency

##### Transcriptomic Response

Copper deficiency produced a markedly asymmetric transcriptomic response, with roots exhibiting substantially more extensive transcriptional changes than leaves. Leaves showed only 37 total DEGs, of which 15 (40.5%) were unique to this treatment, whereas roots showed 443 total DEGs, of which 91 (20.5%) were unique (Supplemental Figure S45A). GO term enrichment of the unique DEG sets showed that in leaves, functional enrichment centered on copper acquisition, chloroplast organization, and lignin metabolism, alongside enrichment of iron-starvation response genes (Supplemental Figure S45B,C). In roots, functional enrichment also identified plastid antioxidant metabolism, including vitamin E, carotenoid, and plastoquinone biosynthesis, as well as copper transport and iron-starvation response genes (Supplemental Figure S45D,E), indicating a broader and more diverse transcriptional response to copper limitation in root tissue.

Gene set enrichment analysis of the full ranked transcriptome reinforced this asymmetry. The leaf GSEA response was minimal, with only two significant GO terms, copper ion transmembrane transport and amino-acid racemase activity (Figure 8I), and suppression of lysine biosynthesis as the sole enriched KEGG pathway (Supplemental Figure S46A,B). In roots, GSEA identified a substantially broader response, with activation of antioxidant and isoprenoid biosynthesis pathways, including vitamin E and carotenoid biosynthesis, alongside suppression of protein homeostasis and stress-response genes (Figure 8J), though no KEGG pathways reached significance. Together, these results indicate that copper deficiency primarily manifests through a modest, targeted response in leaves and a considerably more extensive transcriptional response in roots.

##### Candidate Genes and Regulatory Networks

In leaves, PLS-DA (component 1: 18% variance explained) identified *BHLH160* (VIP = 3.03) and *COPT6* (2.92) among the top discriminating genes, alongside *ZIP2* (2.93), *LAC17* (2.91), and *HEMW* (2.97) (Supplemental Figure S47A,B). GO term enrichment of transcripts with VIP scores ≥ 1.5 identified biological process terms associated with chloroplast organization, plastid translation, and lignin biosynthesis, consistent with the well-established sensitivity of copper-dependent photosynthetic proteins such as plastocyanin to copper deficiency (Supplemental Figure S47C,D). No leaf co-expression modules met the threshold for strong correlation with copper deficiency.

In roots, PLS-DA (component 1: 24% variance explained) identified the same key regulator, *BHLH160* (VIP = 2.57), along with *FRO4* (2.59), *ZIP1* (2.61), and *SODCC* (2.56) among the top discriminating genes (Supplemental Figure S47E,F). GO term enrichment of root VIP transcripts identified biological process terms associated with copper homeostasis, protein turnover, cell cycle regulation, and stress responses, presenting a clear picture of roots actively responding to copper limitation through upregulation of copper acquisition machinery while managing the oxidative consequences of reduced Cu/Zn SOD activity (Supplemental Figure S47G,H). Co-expression analysis identified module ME36 (*r* = 0.96, *p* = 3 × 10^−30^) as strongly associated with copper deficiency—the single strongest module-trait correlation observed across all nutrients and tissues examined in this study (Figure 4B; Supplemental Figure S48A,B). Hub genes within ME36 included *BHLH160*, *COPT6*, *FRO4*, and *YSL2*, together forming a well-characterized copper deficiency response network in which BHLH transcription factors regulate copper transporter and ferric reductase expression to maximize copper acquisition under limiting conditions (Supplemental Figure S48C). Notably, although *SPL7* is a well-established upstream master regulator of copper deficiency responses in Arabidopsis, acting via BHLH transcription factors to induce expression of *FRO4* and other copper-responsive genes [61], the two annotated quinoa *SPL7* orthologs were not differentially expressed among the top VIP-scoring transcripts or present as hub genes in either tissue in the present study, despite the clear presence of its downstream target *FRO4* within ME36.

##### Biological Interpretation

Copper deficiency elicited a markedly tissue-asymmetric response, with roots mounting a substantially larger and more coordinated transcriptional program than leaves. The exceptionally strong correlation of ME36 with copper deficiency (*r* = 0.96) indicates that despite copper deficiency’s comparatively modest DEG counts, it elicits one of the most tightly coordinated transcriptional responses observed across all twelve nutrients, underscoring that the magnitude of differential expression and degree of transcriptional coordination can diverge substantially.

In leaves, the more limited response centered on copper acquisition and chloroplast function, consistent with the well-established sensitivity of copper-dependent photosynthetic proteins such as plastocyanin to copper limitation, where impaired copper supply disrupts electron transport between the cytochrome b6f complex and photosystem I [62]. In roots, the coordinated activation of *BHLH160*, *COPT6*, *FRO4*, and *YSL2* reflects a conserved plant copper deficiency response network in which BHLH transcription factors regulate the expression of copper transporters and ferric reductases to maximize copper acquisition and redistribute metal resources under limiting conditions [61,63,64,65], while the repeated co-enrichment of iron-starvation genes alongside copper transport genes points to cross-regulation between copper and iron homeostasis pathways [66]. The concurrent activation of vitamin E and carotenoid biosynthesis in roots further suggests a compensatory increase in lipophilic antioxidant capacity, potentially offsetting the reduced Cu/Zn superoxide dismutase activity under copper limitation [67,68].

Together, these findings demonstrate that copper deficiency in quinoa is characterized by a highly coordinated, root-centered regulatory response built around a small number of canonical copper-homeostasis genes, despite its comparatively modest impact on overall transcript abundance.

#### 2.6.6. Molybdenum Deficiency

##### Transcriptomic Response

Molybdenum deficiency produced the second-fewest total DEGs, with only 14 total DEGs in leaves and 236 in roots, of which just 2 (14.3%) and 11 (4.7%) were unique to this treatment, respectively (Supplemental Figure S49). The scarcity of condition-specific DEGs is consistent with the relatively small number of molybdenum-dependent enzymes in plants, which are largely restricted to nitrate reductase, sulfite oxidase, xanthine dehydrogenase, and aldehyde oxidase, precluding meaningful GO term enrichment from the unique DEG sets in either tissue.

Gene set enrichment analysis of the full ranked transcriptome likewise identified very little significant signal in either tissue. In leaves, GSEA identified only two enriched GO terms, both activated and related to RNA polymerase II transcriptional regulation, with no KEGG pathways reaching significance (Figure 8K). In roots, GSEA identified a single enriched GO term, regulation of anatomical structure morphogenesis, which was activated, again with no significant KEGG pathways (Figure 8L); this term is consistent with the presence of *EXPA1*, an expansin involved in cell wall loosening and root morphogenesis, among the top VIP genes identified by PLS-DA. Together, these results reinforce that molybdenum limitation elicited comparatively modest and diffuse transcriptional reprogramming at the sampling time point.

##### Candidate Genes and Regulatory Networks

Despite the near-absence of differentially expressed genes, PLS-DA successfully discriminated molybdenum-deficient from control transcriptomes in both tissues. In leaves, PLS-DA (component 1: 22% variance explained) identified *RNR1* (VIP = 2.46), *NIP2-1* (2.45), and *PILS2* (2.47) among the top discriminating genes (Supplemental Figure S50A,B). GO term enrichment of transcripts with VIP scores ≥ 1.5 identified biological process terms associated with ribosome biogenesis and translation, potentially reflecting downstream consequences of impaired nitrate reductase activity on nitrogen assimilation and protein synthesis capacity, along with enrichment of isopentenyl diphosphate metabolism, a pathway contributing to cytokinin biosynthesis (Supplemental Figure S50C,D).

In roots, PLS-DA (component 1: 22% variance explained) identified *EXPA1* (VIP = 2.89), *AHP4* (2.78), *ACX4* (2.80), and *GRXC9* (2.85) among the top discriminating genes (Supplemental Figure S50E,F). GO term enrichment of root VIP transcripts identified biological process terms associated with defense and immune responses, hormone signaling, and secondary metabolism, suggesting substantial reorganization of stress and hormone signaling networks under molybdenum limitation (Supplemental Figure S50G,H). Notably, the identification of *EXPA1* among the top VIP genes was independently corroborated by GSEA, together implicating altered root developmental signaling as a specific, if modest, consequence of molybdenum deficiency. No co-expression modules in either tissue met the threshold for strong correlation with molybdenum deficiency.

##### Biological Interpretation

Despite the essential role of molybdenum in nitrate reductase activity and other molybdoenzymes, molybdenum deficiency produced only modest transcriptional responses in both tissues, consistent with the relatively small number of molybdenum-dependent enzymes in plants [69]. The enrichment of ribosome biogenesis and translation terms among leaf VIP genes likely reflects indirect downstream consequences of impaired nitrate reductase activity, where reduced nitrogen assimilation capacity would be expected to limit amino acid and nucleotide availability for protein synthesis [70], while the enrichment of isopentenyl diphosphate metabolism is notable given its contribution to cytokinin biosynthesis and the documented links between molybdenum-dependent pathways and altered cytokinin levels [71].

The identification of *EXPA1* through both PLS-DA and GSEA in roots suggests that at least one specific, biologically interpretable consequence of molybdenum limitation, altered root morphogenesis, can be detected even amid an otherwise limited transcriptional response, though the overall scarcity of condition-specific signatures may reflect either the relatively mild physiological stress present at the 30-day sampling point or regulation occurring primarily through enzyme activity and post-transcriptional mechanisms rather than large-scale changes in gene expression.

Along with boron deficiency, molybdenum limitation demonstrates that not all nutrient deficiencies elicit extensive transcriptomic reprogramming, indicating that the magnitude of transcriptional responses reflects both the physiological role of the deficient nutrient and the extent to which adaptation is mediated through enzyme-level regulation rather than widespread changes in gene expression.

## 3. Discussion

This study provides the first comprehensive transcriptomic comparison of quinoa responses to the deficiencies of twelve essential macro- and micronutrients across leaf and root tissues. Although individual nutrient deficiencies differed substantially in the magnitude and character of their transcriptional responses, several overarching patterns emerged. Nutrient limitation consistently activated a conserved stress program involving photosynthetic adjustment, oxidative stress responses, protein turnover, ion transport, and nutrient recycling, while each nutrient also induced distinct transcriptional programs reflecting its physiological functions. Together, these findings demonstrate that quinoa responds to nutrient limitation through the integration of generalized stress adaptation and nutrient-specific regulatory mechanisms.

### 3.1. Leaf and Root Tissues Employ Distinct Strategies for Nutrient Stress Adaptation

One of the most consistent findings across all nutrient deficiencies was the notable contrast between leaf and root transcriptomes. Leaves generally exhibited substantially greater transcriptional plasticity than roots, particularly under nitrogen, potassium, magnesium, and zinc deficiency. Iron and copper deficiency were notable exceptions to this pattern, both eliciting larger and more distinctive transcriptional responses in roots than in leaves—consistent with the central role of roots in iron acquisition from the rhizosphere, and, for copper, reflecting an exceptionally coordinated root-specific regulatory network [37,66,72]. This tissue-level contrast is also reflected in the WGCNA soft-thresholding powers selected for each tissue (5 for leaves and 3 for roots): the more diffuse correlation structure of root transcriptomes, consistent with their substantial cross-nutrient similarity described below, reached an adequate scale-free topology fit at a lower power, whereas the more distinctly modular leaf networks, shaped by stronger and more nutrient-specific responses, required a higher power to suppress spurious correlations. Beyond this general pattern of reduced transcriptional plasticity, root responses across nutrients were also characterized by a smaller proportion of condition-specific genes relative to leaves, with substantial overlap in the genes and pathways activated across different deficiencies, particularly regulation of nutrient transport, ion homeostasis, and nutrient recycling pathways; this pattern of shared root responses is examined directly in Section 3.2. These differences reflect the distinct physiological roles of the two tissues: leaves function as the primary site of photosynthesis and carbon assimilation and therefore respond strongly to deficiencies that impair chloroplast function and primary metabolism, whereas roots function primarily in nutrient acquisition and environmental sensing, explaining the consistent enrichment of nutrient transporters, stress signaling pathways, and structural remodeling under multiple nutrient deficiencies [14,20,73]. The contrasting responses observed here suggest that quinoa partitions nutrient-stress adaptation between photosynthetically active tissues and nutrient-acquiring tissues to maintain whole-plant homeostasis.

### 3.2. Conserved Transcriptional Responses Are Concentrated in Root Tissue

The identification of a gene set shared across all twelve nutrient deficiencies in roots (Section 2.4) provides the clearest evidence for a genuinely conserved transcriptional response to nutrient limitation. This shared set was consistently downregulated and enriched for ubiquitin-mediated protein degradation, RNA turnover, defense signaling, and ion homeostasis, suggesting that root transcriptomes activate a common baseline stress program largely independent of the specific nutrient being limited. This finding parallels a recent systems-level analysis of Arabidopsis root transcriptomes across fifteen nutrient and beneficial-element treatments, which similarly identified a core cluster of co-regulated root genes shared across diverse nutrient conditions, enriched for suberin biosynthesis and stress-related metabolic pathways [74]; together, these findings suggest that a shared, nutrient-independent regulatory network coordinating root-level stress responses may be a broadly conserved feature of plant nutrient signaling. In leaves, by contrast, the genes shared among the four deficiencies producing the largest transcriptional responses (nitrogen, potassium, magnesium, and zinc) did not resolve into any significantly enriched biological process, indicating that although these deficiencies share a substantial number of differentially expressed genes, their collective functions are too heterogeneous to constitute a coherent shared program at this level of analysis.

Separately, suppression of photosynthesis-related genes recurred across several distinct nutrient deficiencies in leaves, including nitrogen, potassium, magnesium, iron, and manganese, consistent with the individual nutrient-specific findings described above [7,10,14,41,54]. Because this pattern emerged from each nutrient’s own differential expression and pathway enrichment analyses rather than from genes literally shared among these treatments, it likely reflects convergent, independently arising suppression of photosynthetic capacity as a common consequence of several distinct forms of nutrient stress, rather than a single shared regulatory mechanism. This convergence was not universal, however, as photosynthesis-related genes were activated rather than suppressed in nitrogen-deficient roots, and in zinc-deficient leaves, the photosynthesis antenna protein pathway was activated even as overall photosynthesis-associated genes were suppressed, indicating that the direction of this response depends on both tissue and nutrient context.

Notably, the leaf-root asymmetry described in Section 3.1 was also evident in the shared-gene analysis: only roots produced an interpretable, all-twelve-nutrient-conserved signature, while the leaf-side overlap was functionally uninterpretable despite involving more genes. No genes appeared among the shared sets identified in both the all-nutrient leaf and all-nutrient root intersections, indicating that quinoa does not rely on a single unified transcriptional program spanning both tissues; instead, the conserved response to nutrient limitation is itself organized along tissue-specific lines, distinct from the coordinated but non-overlapping stress programs each tissue deploys independently.

The identification of a conserved root transcriptional program has implications beyond quinoa, though the corresponding absence in leaves is itself a notable finding. Future work examining whether this asymmetry reflects genuine biological differences in how leaves and roots organize stress responses, or whether it results from the specific gene sets and thresholds used to define “shared” DEGs in this study, would help clarify the generality of this pattern.

### 3.3. Macronutrients and Micronutrients Employ Distinct Adaptive Strategies

Comparison of transcriptomic responses across all twelve nutrient deficiencies revealed clear differences between macronutrient and micronutrient limitation. Both classes contributed to the root-centered conserved response described above (Section 3.2), but they differed substantially in the additional, nutrient-specific biological processes most strongly affected. Macronutrient deficiencies generally produced broader transcriptional reprogramming affecting central metabolic processes required for growth and energy production. Nitrogen deficiency strongly suppressed photosynthesis while promoting nutrient recycling; phosphorus deficiency activated phosphate conservation and membrane lipid remodeling; potassium deficiency induced osmotic stress and defense pathways; sulfur deficiency targeted sulfur assimilation and one-carbon metabolism; and magnesium deficiency extensively disrupted ribosome biogenesis and translational machinery. Calcium deficiency was a notable exception among the macronutrients, producing a comparatively limited and diffuse transcriptional signature, with few condition-specific DEGs and no strongly correlated co-expression modules in either tissue. Collectively, these responses indicate that deficiencies of nutrients required in large quantities primarily affect core metabolic functions essential for plant growth and development, though the extent of this reprogramming varies considerably even among macronutrients.

In contrast, micronutrient deficiencies predominantly altered pathways associated with metal homeostasis, oxidative stress, and specialized biochemical functions. Iron deficiency activated coordinated iron acquisition pathways involving metal transport and riboflavin biosynthesis, zinc deficiency strongly regulated genome maintenance and DNA repair, manganese deficiency disrupted photosynthetic electron transport while activating endoplasmic reticulum stress responses, and copper deficiency produced a response dominated by root-centered copper and iron homeostasis pathways, with a comparatively modest leaf response affecting photosynthetic electron transport. Notably, the strongest single module-trait correlations identified anywhere in this study occurred under micronutrient deficiency: copper deficiency in roots (ME36, *r* = 0.96) and manganese deficiency in leaves (ME13, *r* = 0.91), indicating that micronutrient limitation can elicit highly coordinated transcriptional responses even when, as in the case of copper, overall differential expression is comparatively modest. Similar decoupling between differential expression magnitude and co-expression network coherence has been reported in other nutrient-deficient plant systems, including rice under macronutrient limitation [75] and peanut under calcium deficiency [76], where a small number of differentially expressed genes nonetheless resolved into functionally coherent, tightly coordinated co-expression modules. Boron and molybdenum deficiencies resulted in comparatively limited transcriptional responses, suggesting that adaptation to these nutrient deficiencies depends less on large-scale transcriptional reprogramming than on structural or enzymatic regulation.

Cross-regulation among metal ion homeostasis networks emerged as a recurring theme among the micronutrients: copper deficiency was associated with repeated activation of iron-starvation response genes across the unique DEG, WGCNA, and GSEA analyses, while zinc deficiency showed co-enrichment of iron- and zinc-related transport functions, consistent with the extensive regulatory interactions known to exist among transition metal homeostasis pathways [66,77]. A related pattern extended beyond the micronutrients: phosphorus deficiency was similarly associated with enrichment of iron-starvation response genes in leaves, consistent with recently characterized crosstalk between phosphorus and iron signaling, including the HRZ-PHR regulatory module, in which phosphorus deficiency represses HRZ-mediated degradation of the PHR1 transcription factor, directly linking phosphorus status to iron homeostasis regulation [78]. Similarly, activation of DNA repair pathways, including mismatch repair, base excision repair, and homologous recombination, was shared between iron and zinc deficiencies in leaves, suggesting a common consequence of disrupting micronutrient-dependent processes involved in genome maintenance [45,79].

These contrasting patterns likely reflect fundamental differences in the biological roles of macro- and micronutrients. Macronutrients are required in relatively large quantities as structural components of proteins, nucleic acids, membranes, and other primary metabolites, whereas micronutrients primarily function as enzyme cofactors, metal centers, and signaling components. Consequently, macronutrient limitation broadly disrupts primary metabolism, whereas micronutrient limitation preferentially affects specialized metabolic pathways that depend on specific metal cofactors. Despite these differences, both nutrient classes ultimately converge on common stress-response pathways that maintain cellular homeostasis during nutrient limitation.

### 3.4. Implications for Improving Nutrient Use Efficiency in Quinoa

The comprehensive nature of this study provides a valuable resource for understanding nutrient stress adaptation in quinoa and for identifying candidate genes that may improve nutrient use efficiency through breeding or functional genomics. Across all nutrient deficiencies, several regulatory genes, transporters, and co-expression modules consistently emerged as central components of nutrient stress responses, including regulators of nutrient transport, transcriptional control, oxidative stress, and cellular homeostasis. Genes supported by convergent evidence across multiple independent analytical approaches, or identified in both leaf and root tissue, represent particularly strong candidates for initial functional validation. For example, *BHLH160* and *COPT6* were identified as top discriminating genes in both leaf and root PLS-DA analyses and as hub genes within the strongly correlated root copper-responsive co-expression module (ME36, r = 0.96), consistent with their established roles in copper acquisition in other plant species [64,65,72]. Similarly, *NLP3*, a nitrate-responsive transcription factor with established roles in nitrogen use efficiency in rice and quinoa, was among the strongest discriminating genes in nitrogen-deficient roots. Genes such as these, which combine strong statistical support within this dataset with prior functional characterization in the literature, may be reasonably prioritized over genes identified through only a single analytical approach or lacking prior functional annotation. These genes provide promising targets for future validation using reverse genetics, gene editing, and marker-assisted breeding.

Beyond identifying nutrient-specific candidate genes, the comparative design of this study provides a broader framework for understanding how plants integrate responses to nutrient limitation. The identification of a conserved transcriptional program shared across multiple deficiencies suggests that improving general stress resilience may simultaneously enhance tolerance to multiple nutrient limitations, whereas nutrient-specific regulators may provide opportunities to improve the acquisition or utilization of individual nutrients. Such complementary strategies may prove particularly valuable for developing cultivars adapted to nutrient-poor or marginal soils.

Finally, this study establishes a standardized comparative framework for investigating nutrient stress responses in quinoa. Because all twelve essential nutrient deficiencies were characterized using the same genotype, developmental stage, experimental design, sequencing platform, and analytical pipeline, the resulting dataset enables direct comparison of transcriptomic responses across nutrient limitations. The publicly available RNA-seq dataset generated in this study provides a valuable resource for future comparative analyses of nutrient stress responses in quinoa. Integration of these transcriptomic data with future physiological, metabolomic, proteomic, and quantitative genetic studies will further improve understanding of nutrient stress adaptation and accelerate development of quinoa cultivars with enhanced nutrient use efficiency and resilience to nutrient-limited environments.

### 3.5. Limitations and Future Directions

Although this study provides the first comprehensive comparison of transcriptomic responses to deficiencies of twelve essential nutrients in quinoa, several limitations should be considered when interpreting the results. First, the analyses were conducted using a single quinoa genotype (QQ74), and nutrient stress responses may vary among genetically diverse cultivars. Future studies incorporating multiple cultivars may help elucidate ecotype-specific responses. Second, transcriptomes were sampled at a single developmental stage and time point (30 days after planting), providing a snapshot of nutrient responses rather than their temporal progression. Future time-course experiments will be important for distinguishing early signaling events from longer-term physiological adaptation.

The hydroponic growth system used in this study provided precise control over nutrient availability and enabled direct comparison among nutrient deficiencies. However, nutrient dynamics in field soils are considerably more complex, and interactions among soil chemistry, microbial communities, and environmental conditions typically buffer nutrient decline, allowing it to develop more gradually than the abrupt and continuous limitation imposed by this hydroponic system. Consequently, the transcriptional responses characterized here may reflect a more rapid and pronounced deficiency response than would typically develop under field conditions, and this distinction should be considered when extrapolating these findings to agricultural settings. In addition, because deficiency treatments were created by reducing or omitting a single nutrient from an otherwise complete solution, treatments may have differed slightly in electrical conductivity and overall ionic balance in addition to the availability of the targeted nutrient, a common limitation of single-nutrient-omission hydroponic designs that could be addressed in future work through conductivity-matched control solutions.

Finally, transcript abundance does not necessarily reflect protein abundance, enzyme activity, or metabolite accumulation, and candidate genes identified here were not independently validated using RT-qPCR. Integration of transcriptomic data with proteomic, metabolomic, and quantitative genetic analyses, together with RT-qPCR or functional validation of candidate genes, particularly those involved in nutrient transport, transcriptional regulation, riboflavin biosynthesis, membrane lipid remodeling, and translational regulation, represents an important next step toward improving nutrient use efficiency in this emerging crop.

## 4. Materials and Methods

### 4.1. Plant Material and Experimental Design

Quinoa accession QQ74 (PI 614886), for which a chromosome-scale reference genome and comprehensive gene annotation are available [80,81], was used to characterize transcriptomic responses to nutrient deficiency. Plants were grown hydroponically under controlled environmental conditions using a 12.5 h light/11.5 h dark photoperiod at 25 ± 1 °C during the light period and 15 ± 1 °C during the dark period. Seedlings were established in aerated 14 L hydroponic containers, each fitted with eight hydroponic baskets sown with quinoa seed. Containers were shielded from light to prevent root-zone light exposure, and baskets were watered with deionized water until roots extended into the nutrient solution below, after which plants relied exclusively on hydroponic nutrient uptake.

The experiment consisted of thirteen nutrient treatments arranged in a randomized complete block design with four biological replicates (Supplemental Table S5). A control treatment, containing optimal concentrations of all essential nutrients based on previously established experiments [5,82] with minor modifications informed by preliminary trials, served as the baseline for all twelve nutrient deficiency treatments (Table 2). The remaining treatments imposed deficiency of a single nutrient while maintaining all other nutrients at control concentrations. Nutrient concentrations were reduced by 90% for N, P, K, S, Ca, and Mg; by 95% for Fe and Zn; and omitted completely for Mn, Cu, B, and Mo, following protocols established by Cole et al. [5] and Ioannou et al. [78]. Because this experiment also supported downstream phenotypic and yield evaluations requiring plants to survive to seed set, complete omission of iron and zinc was avoided after early observations indicated it would result in plant mortality before maturity; concentrations for these two nutrients were therefore adjusted to a 95% reduction at 21 DAP, applied uniformly across all four replicate blocks for the full treatment duration, allowing plants to survive through both the RNA-sequencing sampling point and later phenotypic measurements.

All nutrient solutions were prepared using the Hopkins single-nutrient-source hydroponic formulation adapted from previous methods [5,82], with ammonium nitrate; orthophosphoric, sulfuric, hydrochloric, boric, and nitric acids; calcium, magnesium, zinc, manganese, and copper carbonates; potassium hydroxide; sodium molybdate; iron EDDHA; HEDTA chelate; and MES buffer as nutrient sources. Nutrient solutions were continuously aerated throughout the experiment, and solution pH was maintained between 7.2 and 7.8 by weekly adjustment with HCl or NaOH. Although chloride is a recognized essential micronutrient, it was excluded from this nutrient panel due to the difficulty of controlling ambient contamination and established challenges in accurately quantifying chloride deficiency.

### 4.2. Tissue Collection

Following seedling establishment, plants were thinned to one plant per hydroponic basket, resulting in eight plants per container. At 30 days after planting (DAP), one plant was harvested from each treatment within each of the four replicate blocks, yielding four biological replicates per treatment. Leaf tissue was sampled from the upper, middle, and lower canopy and pooled into a single sample per plant, whereas the entire root system was rinsed three times with deionized water prior to collection. All tissues were immediately flash-frozen in liquid nitrogen and stored at −80 °C until RNA extraction. In total, 104 leaf and root samples were collected across all 13 nutrient treatments and four biological replicates for RNA sequencing.

### 4.3. RNA Extraction and Sequencing

Flash-frozen leaf and root tissues were ground in liquid nitrogen, homogenized in TRI Reagent, and stored at −80 °C until RNA extraction. Total RNA was isolated using the Direct-zol RNA MiniPrep Plus Kit (Zymo Research, Irvine, CA, USA) according to the manufacturer’s instructions. RNA quantity and purity were initially assessed using a NanoDrop™ One/OneC microvolume spectrophotometer (Thermo Fisher Scientific, Waltham, MA, USA), and RNA integrity was evaluated with an Agilent 2100 Bioanalyzer (Agilent Technologies, Santa Clara, CA, USA). Samples with A260/A280 and A260/A230 ratios > 1.8 and an RNA integrity number (RIN) > 7 were used for library preparation. Poly(A)-selected mRNA libraries were constructed and sequenced by Novogene (Davis, CA, USA) on an Illumina NovaSeq X Plus platform (San Diego, CA, USA) using 2 × 150 bp paired-end sequencing, targeting approximately 30 million read pairs per sample.

### 4.4. Read Processing, Mapping, and Transcript Quantification

Raw sequencing reads were evaluated using FastQC v0.11.9 [83] and trimmed to remove residual Illumina adapter sequences using Trimmomatic v0.39 [84]. Potential contamination was assessed by classifying trimmed reads with Kraken2 v2.1.2 [85] against the PlusPFP database, followed by species-level abundance estimation with Bracken v2.6.1 [86]. The proportion of reads assigned to Viridiplantae was used to confirm that the sequenced libraries were predominantly plant-derived.

Quality-filtered reads were aligned to the *C. quinoa* QQ74 v2.0 reference genome using HISAT2 v2.2.1 [87] with the -k 10 option to retain up to ten alignments per read, enabling multi-mapping for downstream transcript quantification. Alignments were converted to sorted BAM files using SAMtools v1.19.2 [88]. Gene-level read counts were generated with featureCounts from the Subread v2.0.8 package [89] using the -M --fraction options to retain multi-mapping reads and assign fractional counts. Gene-level counts from all samples were merged using the QQ74 v2.0 gene identifiers from the annotation by Jaggi et al. [90] into a count matrix for subsequent differential expression analyses.

### 4.5. Differential Expression Analysis

Differential expression analyses were performed separately for leaf and root tissues using DESeq2 v1.48.2 [91] in R v4.5.2. Because multi-mapping reads were assigned fractional counts during transcript quantification, gene counts were rounded to the nearest integer prior to analysis. A DESeqDataSet was constructed for each tissue using a single-factor design (~condition), where condition denotes the nutrient-deficiency treatment. Pairwise comparisons were performed between each nutrient deficiency treatment and the control. Log_2_FC values were shrunk using the adaptive shrinkage estimator (ashr) implemented in the lfcShrink function [92]. Genes with a *p*-adj < 0.05 (Benjamini–Hochberg correction) and an absolute log_2_FC ≥ 1 were considered differentially expressed.

### 4.6. Replicate Validation and Correlation Assessment

Sample relationships were evaluated using PCA and Pearson correlation analyses based on variance-stabilizing transformed (VST) counts generated with the vst function in DESeq2. PCA was performed separately for each tissue, with blind = FALSE to preserve variance associated with the experimental treatments, enabling assessment of overall sample clustering and identification of potential outliers or batch effects. PCA figures were generated with ggplot2 v4.0.2 [93], and sample labels were added using ggrepel v0.9.8 [94]. Pearson correlation heatmaps were generated for all combined samples as well as separately for each treatment, including the control, using pheatmap v1.0.13 [95] with VST-transformed counts generated with blind = TRUE to assess reproducibility among biological replicates independent of the experimental design. Rows and columns were hierarchically clustered using correlation-based distances and average linkage.

To assess the sensitivity of differential expression estimates to individual biological replicates, a leave-one-out analysis was performed for each nutrient-deficiency comparison in leaf and root tissue. For each tissue, DESeq2 was fit to the complete multi-condition dataset, and the model was iteratively refitted while excluding one treatment replicate at a time, preserving the pooled dispersion estimate across all conditions rather than isolating each pairwise comparison. Robustness was quantified as the Pearson correlation between log_2_FC estimates from the full-replicate model and each leave-one-out iteration, calculated both across all genes and within confidence tiers (*p-*adj < 0.05, <0.01, <0.001) to distinguish genuine instability in effect-size estimates from expected power loss among lower-confidence genes. Mean correlation values across replicates were visualized as a heatmap for each nutrient-tissue combination and confidence tier, allowing treatments with consistently high replicate robustness to be distinguished from those showing greater sensitivity to individual samples.

### 4.7. Differential Expression Visualization

Differential expression results were visualized using volcano plots generated with ggplot2, in which genes were plotted according to log_2_FC and −log_10_ *p*-adj. To facilitate comparisons across nutrient-deficiency treatments, volcano plots were displayed on a common *y*-axis scale. The numbers of upregulated and downregulated genes were summarized for each treatment and tissue in a grouped bar plot.

To visualize differential expression patterns across nutrient treatments and tissues, genes significant in at least one nutrient-vs-control comparison were compiled into a combined heatmap displaying log_2_ fold-change values across all conditions. For visualization purposes, the color scale was capped at ±5 log_2_FC; genes with fold-change values exceeding this threshold were displayed at the maximum color intensity rather than being excluded from the analysis. Hierarchical clustering (Euclidean distance, average linkage) was applied to both genes and conditions using pheatmap v1.0.13 to group genes with similar expression patterns across the dataset.

Overlap among DEGs across nutrient deficiency treatments was evaluated using UpSetR v1.4.0 [96], with intersections containing fewer than ten genes were excluded to improve visualization. Shared DEGs identified from major UpSet intersections were visualized as clustered heatmaps using pheatmap v1.0.13, with rows hierarchically clustered; where multiple DEGs shared the same annotated gene name, only the entry with the largest absolute log_2_FC was retained for visualization. Shared DEGs were functionally annotated using overrepresentation analysis (ORA) as described in Section 4.8.

### 4.8. Functional Annotation and Enrichment Analysis

Transcript sequences were functionally annotated using TRAPID [97], with GO annotations assigned using the PLAZA 4.5 dicots database. This annotation served as the basis for both overrepresentation and gene set enrichment analyses described below.

Overrepresentation analysis was performed using TRAPID’s built-in enrichment test to identify GO terms enriched among genes of interest. This approach was applied to condition-specific DEGs identified from UpSet plot intersections, VIP-scoring genes from PLS-DA, and genes belonging to WGCNA co-expression modules of interest, as described in the corresponding sections below. Enriched biological process (BP) and molecular function (MF) GO terms identified from ORA were clustered according to semantic similarity using rrvgo v1.20.0 [98] with GO term relationships derived from the *Arabidopsis thaliana* genome-wide annotation (org.At.tair.db) as the reference database.

Gene Set Enrichment Analysis was performed separately to evaluate GO and KEGG pathway enrichment across the full ranked transcriptome for each treatment–tissue comparison. Transcript-level GO annotations from TRAPID were aggregated to gene-level annotations and formatted as custom TERM2GENE and TERM2NAME tables for use with clusterProfiler v4.16.0 [99]. GSEA was performed separately for each treatment–tissue comparison using ranked gene lists ordered by log_2_FC, with Benjamini–Hochberg multiple testing correction (*p <* 0.05). Enriched BP and MF terms were summarized by semantic similarity using rrvgo v1.20.0, and results were visualized as dot plots showing the top 15 enriched terms per direction.

KEGG pathway enrichment was performed using gseKEGG in clusterProfiler. Quinoa protein sequences were aligned to the RefSeq quinoa genome annotation (GCF_001683475.1) using BLASTP [100], retaining the best hit per transcript based on bit score. RefSeq protein accessions were mapped to NCBI GeneIDs using GFF annotations and converted to quinoa KEGG identifiers (organism code: cqi), retaining the highest-scoring hit per gene where multiple transcripts mapped to the same NCBI GeneID. GSEA was performed with a *p-*value cutoff of 0.05, Benjamini–Hochberg correction, and a fixed random seed to ensure reproducibility. Enriched pathways were visualized as dot plots showing the top 15 terms, faceted by enrichment direction, and pathways with a false discovery rate <0.05 were additionally visualized using Pathview v1.48.0 [101], with gene-level log_2_FC values mapped onto native KEGG pathway diagrams.

### 4.9. Multivariate and Co-Expression Network Analyses

Partial least squares discriminant analysis was performed using the mixOmics v6.32.0 package [102] in R to identify genes that best discriminated each nutrient deficiency treatment from the control within leaf and root tissues. Analyses were performed separately for each treatment–control comparison using variance-stabilizing transformed expression values. Model performance was evaluated using four-fold cross-validation with 50 repeats. Variable importance in projection scores were calculated for each gene on component 1, and the top 20 genes for each comparison were retained for reporting. Genes with VIP scores ≥ 1.5 were used for downstream functional enrichment analyses.

Weighted gene co-expression network analysis (WGCNA) was performed separately for leaf and root tissues using the WGCNA v1.74 package [103]. Sample and gene quality were assessed using goodSamplesGenes, and flagged observations were excluded. Networks were constructed from VST-transformed expression values (blind = FALSE) after removing the lowest 25% of genes based on expression variance. Signed co-expression networks and a signed topological overlap matrix (TOM) were generated using blockwiseModules, with soft-thresholding powers selected according to the scale-free topology criterion (R^2^ ≥ 0.80); powers of 5 and 3 were selected for leaves and roots, respectively. Minimum module size and module merging parameters were set to 30 genes and a merge cut height of 0.25, respectively.

Module eigengenes were correlated with nutrient deficiency treatments using Pearson correlation. Modules with correlation coefficients ≥ 0.5 and *p-*values < 0.05 were considered for further analysis. Hub genes were identified based on module membership (kME), and the top 20 genes with the highest absolute kME values were retained for each module. Co-expression relationships among hub genes were visualized in Cytoscape v3.10.4 [104] using edge weights derived from the topological overlap matrix (TOM). Functional annotation and enrichment analyses of PLS-DA VIP genes and genes within nutrient-associated WGCNA modules were performed using ORA as described in Section 4.8.

## 5. Conclusions

This study provides the first comprehensive comparative transcriptomic analysis of quinoa responses to deficiencies of all essential macro- and micronutrients across leaf and root tissues. Although the magnitude and character of transcriptional responses differed substantially among nutrient deficiencies, several overarching patterns emerged. A conserved transcriptional program, most evident in roots and centered on protein turnover, ion transport, and nutrient recycling, was activated across nearly all nutrient deficiencies, while individual nutrient deficiencies also elicited specialized regulatory pathways that reflect their distinct physiological functions. Suppression of photosynthesis-related genes recurred independently across several nutrient deficiencies in leaves, suggesting a convergent, though not uniformly shared, consequence of nutrient stress on photosynthetic capacity. Macronutrient deficiencies primarily affected central metabolic processes, including photosynthesis, translation, membrane remodeling, and nutrient assimilation, whereas micronutrient deficiencies predominantly altered metal homeostasis, oxidative stress responses, and specialized cofactor-dependent pathways. The pronounced differences between leaf and root transcriptomes further demonstrate that nutrient stress adaptation is coordinated through complementary tissue-specific regulatory strategies.

Notably, the magnitude, tissue distribution, and coordination of these transcriptional responses did not always align; some deficiencies produced extensive differential expression alongside comparatively modest co-expression network organization, while others showed the reverse. This underscores the value of integrating multiple complementary analytical approaches when characterizing nutrient stress responses. Together, these findings provide new insight into the molecular basis of nutrient stress adaptation in quinoa and establish a standardized comparative framework for investigating nutrient deficiency responses across essential mineral nutrients. The publicly available RNA-seq dataset generated in this study provides a valuable resource for future comparative analyses, while the candidate genes and regulatory pathways identified here provide promising targets for functional validation, improving nutrient use efficiency, and developing quinoa cultivars better adapted to nutrient-limited environments.

## Figures and Tables

**Table 1:** Physical effects of macro- and micronutrient deficiencies across all quinoa plants at 30 days after planting. Visual rating scores range from 0—extreme chlorosis, stunted growth, very unhealthy, to 5—plant is green, growth is robust, very healthy. Height measurements represent bucket averages for each block.

Treatment	Block	Visual Rating	Phenotype	Height (cm)	NDVI	Shoot Dry Mass (g)	Root Dry Mass (g)
Ctrl	I	5.0	No visual abnormalities	11.8	0.74	6.7	2.11
	II	5.0	No visual abnormalities	11.4	0.73	8.6	2.2
	III	5.0	No visual abnormalities	9.6	0.72	6.4	2.3
	IV	5.0	No visual abnormalities	10.8	0.68	7.7	2.31
N	I	2.5	Tight, curled roots; general chlorosis in old leaves	6.7	0.49	3.6	2.18
	II	2.6	Tight, curled roots; general chlorosis in old leaves	6.9	0.41	3	2
	III	3.3	Tight, curled roots; general chlorosis in old leaves	7.9	0.45	3.7	2.29
	IV	2.5	Tight, curled roots; general chlorosis in old leaves	9.0	0.31	3.2	1.8
P	I	5.0	No visual abnormalities	10.7	0.65	7.4	2.6
	II	4.0	No visual abnormalities	6.7	0.58	4.3	1.47
	III	4.5	No visual abnormalities	8.3	0.6	6.6	1.9
	IV	4.5	General chlorosis	8.0	0.57	7.9	1.9
K	I	2.3	Marginal chlorosis, deformed leaves, mottling	8.8	0.23	3.4	1.9
	II	3.5	General chlorosis in new leaves	5.8	0.25	2.7	1.3
	III	2.3	General chlorosis, deformed leaves, mottling	7.2	0.34	2.6	1.01
	IV	2.3	Marginal chlorosis, deformed leaves, mottling	9.1	0.23	3.7	1.3
S	I	4.5	General chlorosis	10.4	0.51	7.9	2.7
	II	3.8	General chlorosis in new leaves	6.2	0.47	7.5	1.39
	III	4.2	General chlorosis	10.1	0.66	-	2.16
	IV	4.8	No visual abnormalities	10.0	0.5	-	-
Ca	I	4.2	Interveinal chlorosis in new leaves	10.6	0.56	8.3	2.54
	II	2.0	Interveinal chlorosis, mottling	5.8	0.25	2	0.7
	III	2.3	Interveinal chlorosis, mottling	7.3	0.31	2.8	0.8
	IV	2.8	Interveinal chlorosis, mottling	10.1	0.44	4	1.1
Mg	I	1.2	Interveinal chlorosis, mottling	4.9	0.09	1.5	0.3
	II	2.2	Interveinal chlorosis, mottling	7.9	0.17	3.5	0.92
	III	2.8	Interveinal chlorosis, mottling, deformed leaves	8.2	0.49	4.5	1.1
	IV	2.9	Interveinal chlorosis, mottling, deformed leaves	11.7	0.39	8.6	1.5
Zn	I	1.5	General chlorosis	4.2	0.19	0.5	0.15
	II	0.8	General chlorosis, mottling	2.0	0.03	0.08	0.04
	III	1.5	General chlorosis	3.3	0.07	0.2	0.14
	IV	1.0	General chlorosis	2.9	0.03	0.1	0.2
Fe	I	0.8	Marginal chlorosis	2.9	0.06	0.1	0.02
	II	0.5	General chlorosis	2.1	0.06	0.07	0.02
	III	2.0	Interveinal chlorosis	5.1	0.31	0.5	0.12
	IV	2.0	Interveinal chlorosis	5.1	0.13	0.5	0.14
Mn	I	2.0	Interveinal chlorosis, mottling	9.1	0.55	9.6	1.56
	II	1.8	Interveinal chlorosis, mottling	5.3	0.3	8.6	0.62
	III	2.8	Interveinal chlorosis, mottling	7.4	0.46	11.9	1.7
	IV	2.9	Interveinal chlorosis, mottling	9.2	0.41	6.6	1.6
B	I	4.3	Slight chlorosis in new leaves	7.6	0.42	5.5	2.1
	II	4	General chlorosis	6.9	0.55	5.2	1.92
	III	4.9	General chlorosis in new leaves	9.3	0.67	7.7	2.4
	IV	4.8	No visual abnormalities	9.4	0.63	16.6	2.4
Cu	I	3.7	General chlorosis in new leaves	6.7	0.34	3	0.8
	II	4.2	General chlorosis in new leaves	8.5	0.48	4.7	1.1
	III	3.5	Interveinal chlorosis	7.1	0.38	2.6	0.83
	IV	4.2	General chlorosis	8.3	0.4	5.2	1.2
Mo	I	4.3	No visual abnormalities	8.8	0.48	-	1.2
	II	4.6	Slight chlorosis	8.8	0.52	-	1.6
	III	4.3	No visual abnormalities	7.8	0.52	-	1.9
	IV	4.9	No visual abnormalities	9.3	-	-	1.68

**Table 2:** Nutrient solution concentrations for control and deficient treatments used in the hydroponics experiment.

	Nutrient	Source	Optimal Trts (μM)	Deficient Trts (μM)
Concentrated strong acids	Cl	Hydrochloric acid	39.8	n/a
	S	Sulfuric acid	2148	214.8
	P	Orthophosphoric acid	750	75
	N	Nitric acid	12,000	1200
Salts added as solids	Ca	Calcium carbonate	3961	396.1
	Mg	Magnesium carbonate	1500	150
Salts added as liquids	-	HEDTA	64	n/a
	Zn	Zinc carbonate	4.2	0.2
	Mn	Manganese acetate	8.11	-
	Cu	Copper carbonate	1.5	-
	B	Boric acid	18.1	-
	Mo	Sodium molybdate	0.22	-
	N	Ammonium nitrate	3000	300
	K	Potassium carbonate	6000	600
	Fe	Iron EDDHA	27	1.4
	-	MES	10,000	n/a

## Data Availability

RNA sequence data generated in this study have been deposited in GenBank under the BioProject ID PRJNA1484747 (Supplemental Table S6). To make the transcriptomic data generated in this study broadly accessible to the research community, we collaborated with the Bio-Analytic Resource for Plant Biology (BAR) at the University of Toronto to develop a *Chenopodium quinoa* nutrient deficiency electronic Fluorescent Pictograph (eFP) Browser. The eFP Browser is an interactive web-based visualization tool that allows users to query individual genes from the annotation by Jaggi et al. [90] by their QQ74 v2.0 gene identifier and visualize relative expression levels across all twelve nutrient deficiency treatments in both leaf and root tissue simultaneously, displayed as color-coded plant diagrams ranging from blue (downregulated) to red (upregulated) on a log_2_ ratio scale. It complements the broader dataset presented in this study and is intended to serve as a long-term community resource for functional genomic investigation of nutrient use efficiency, stress adaptation, and gene discovery in quinoa and related *Chenopodium* species. The browser is publicly accessible at https://bar.utoronto.ca/efp_quinoa/cgi-bin/efpWeb.cgi.

## Supplementary Materials

The following supporting information can be downloaded at: https://www.mdpi.com/article/10.3390/plants15182828/s1, Figure S1: Pearson correlation heatmap of all 104 replicates across the positive control and deficiency treatments; Figure S2: Pairwise Pearson correlation heatmaps of individual leaf biological replicates for each treatment condition; Figure S3: Pairwise Pearson correlation heatmaps of individual root biological replicates for each treatment condition; Figure S4: Leave-one-out replicate robustness across all nutrient-deficiency comparisons; Figure S5: Volcano plots displaying differential gene expression in leaf samples for each nutrient deficiency treatment relative to the control; Figure S6: Volcano plots displaying differential gene expression in root samples for each nutrient deficiency treatment relative to the control; Figure S7: Condition-specific differentially expressed genes (DEGs) under nitrogen deficiency in leaves and roots; Figure S8: Gene set enrichment analysis (GSEA) results for nitrogen-deficient leaf and root transcriptomes; Figure S9: Partial least squares discriminant analysis (PLS-DA) of nitrogen-deficient and control transcriptomes in leaves and roots; Figure S10: Co-expression module characterization for module eigengenes strongly correlated with nitrogen deficiency in leaves and roots; Figure S11: Condition-specific differentially expressed genes (DEGs) under phosphorus deficiency in leaves and roots; Figure S12: Gene set enrichment analysis (GSEA) results for phosphorus-deficient leaf and root transcriptomes; Figure S13: Partial least squares discriminant analysis (PLS-DA) of phosphorus-deficient and control transcriptomes in leaves and roots; Figure S14: Co-expression module characterization for module eigengenes strongly correlated with phosphorus deficiency in leaves and roots; Figure S15: Condition-specific differentially expressed genes (DEGs) under potassium deficiency in leaves and roots; Figure S16: Gene set enrichment analysis (GSEA) results for potassium-deficient leaf and root transcriptomes; Figure S17: Partial least squares discriminant analysis (PLS-DA) of potassium-deficient and control transcriptomes in leaves and roots; Figure S18: Co-expression module characterization for module eigengenes strongly correlated with potassium deficiency in leaves; Figure S19: Condition-specific differentially expressed genes (DEGs) under sulfur deficiency in leaves and roots; Figure S20: Gene set enrichment analysis (GSEA) results for the sulfur-deficient leaf transcriptome; Figure S21: Partial least squares discriminant analysis (PLS-DA) of sulfur-deficient and control transcriptomes in leaves and roots; Figure S22: Co-expression module characterization for module eigengenes strongly correlated with sulfur deficiency in leaves and roots; Figure S23: Condition-specific differentially expressed genes (DEGs) under calcium deficiency in leaves and roots displayed as a stacked bar chart; Figure S24: Gene set enrichment analysis (GSEA) results for the calcium-deficient leaf transcriptome; Figure S25: Partial least squares discriminant analysis (PLS-DA) of calcium-deficient and control transcriptomes in leaves and roots; Figure S26: Condition-specific differentially expressed genes (DEGs) under magnesium deficiency in leaves and roots; Figure S27: Gene set enrichment analysis (GSEA) results for the magnesium-deficient leaf transcriptome; Figure S28: Partial least squares discriminant analysis (PLS-DA) of magnesium-deficient and control transcriptomes in leaves and roots; Figure S29: Co-expression module characterization for module eigengenes strongly correlated with magnesium deficiency in leaves and roots; Figure S30: Condition-specific differentially expressed genes (DEGs) under zinc deficiency in leaves and roots; Figure S31: Gene set enrichment analysis (GSEA) results for zinc-deficient leaf and root transcriptomes; Figure S32: Partial least squares discriminant analysis (PLS-DA) of zinc-deficient and control transcriptomes in leaves and roots; Figure S33: Co-expression module characterization for module eigengenes strongly correlated with zinc deficiency in leaves and roots; Figure S34: Condition-specific differentially expressed genes (DEGs) under iron deficiency in leaves and roots; Figure S35: Gene set enrichment analysis (GSEA) results for iron-deficient leaf and root transcriptomes; Figure S36: Partial least squares discriminant analysis (PLS-DA) of iron-deficient and control transcriptomes in leaves and roots; Figure S37: Co-expression module characterization for module eigengenes strongly correlated with iron deficiency in leaves and roots; Figure S38: Condition-specific differentially expressed genes (DEGs) under manganese deficiency in leaves and roots; Figure S39: Gene set enrichment analysis (GSEA) results for the manganese-deficient leaf transcriptome; Figure S40: Partial least squares discriminant analysis (PLS-DA) of manganese-deficient and control transcriptomes in leaves and roots; Figure S41: Co-expression module characterization for module eigengenes strongly correlated with manganese deficiency in leaves; Figure S42: Condition-specific differentially expressed genes (DEGs) under boron deficiency in leaves and roots; Figure S43: Gene set enrichment analysis (GSEA) results for boron-deficient leaf and root transcriptomes; Figure S44: Partial least squares discriminant analysis (PLS-DA) of boron-deficient and control transcriptomes in leaves and roots; Figure S45: Condition-specific differentially expressed genes (DEGs) under copper deficiency in leaves and roots; Figure S46: Gene set enrichment analysis (GSEA) results for copper-deficient leaf transcriptomes; Figure S47: Partial least squares discriminant analysis (PLS-DA) of copper-deficient and control transcriptomes in leaves and roots; Figure S48: Co-expression module characterization for module eigengenes strongly correlated with copper deficiency in roots; Figure S49: Condition-specific differentially expressed genes (DEGs) under molybdenum deficiency in leaves and roots displayed as a stacked bar chart; Figure S50: Partial least squares discriminant analysis (PLS-DA) of molybdenum-deficient and control transcriptomes in leaves and roots; Table S1: Paired-end read summary statistics for all RNA-seq samples and replicates; Table S2: Quality control metrics for all RNA-seq replicates; Table S3: Mapping statistics for paired-end reads mapped to the QQ74 v2.0 reference; Table S4: Domain-level taxonomic classification summary of RNA-seq reads across all samples; Table S5: Randomized complete block design for the hydroponics experiment involving four blocks with the positive control and all 12 macro- and micronutrient deficiencies; Table S6: RNA sequencing resources and data availability.
