# Supplementary material for: Comparative Transcriptomic Analysis Reveals Conserved and Nutrient-Specific Responses to Nutrient Deficiencies in Quinoa
Source: Plants (Basel). 2026 Sep 15;15(18):2828. doi: 10.3390/plants15182828 (PMC13610311; doi:10.3390/plants15182828)

Supplementary Material

Supplemental Tables

Supplemental Table S1. Paired-end read summary statistics for all RNA-seq samples and replicates

Sample	Raw reads	Raw data	Effective(%)	Error(%)	Q20(%)	Q30(%)	GC(%)
B_L_1	64723864	9708579600	93.34	0.01	99.22	96.71	44.07
B_L_2	77542732	1.16E+10	95.82	0.01	99.26	96.87	44.05
B_L_3	66990978	1.00E+10	93.98	0.01	99.3	97.14	44.13
B_L_4	61179366	9176904900	93.6	0.01	98.88	96.81	44.12
B_R_1	67758808	1.02E+10	93.15	0.01	99.22	96.78	42.98
B_R_2	67786812	1.02E+10	89.56	0.01	99.31	97.17	42.52
B_R_3	65837992	9875698800	92.65	0.01	99.15	96.49	42.82
B_R_4	68799118	1.03E+10	94.91	0.01	99.03	97.21	42.48
Ca_L_1	81053608	1.22E+10	93.38	0.01	99.22	96.73	44.04
Ca_L_2	84101006	1.26E+10	94.82	0.01	99.21	96.76	44.14
Ca_L_3	71408094	1.07E+10	92.66	0.01	99.33	97.16	44.13
Ca_L_4	72251456	1.08E+10	92.87	0.01	98.94	97.05	44.06
Ca_R_1	81661842	1.22E+10	91.85	0.01	98.89	95.4	42.79
Ca_R_2	84088510	1.26E+10	94.85	0.01	99.19	96.75	42.7
Ca_R_3	70843080	1.06E+10	96.49	0.01	99.33	97.31	42.65
Ca_R_4	71620916	1.07E+10	96.23	0.01	99.09	97.46	42.53
Ctrl_L_1	72923046	1.09E+10	94.78	0.01	99.27	96.83	43.9
Ctrl_L_2	74497276	1.12E+10	94.96	0.01	99.19	96.6	43.82
Ctrl_L_3	87941098	1.32E+10	93.52	0.01	99.34	97.19	44.13
Ctrl_L_4	69908596	1.05E+10	93.81	0.01	99.31	97.11	44.31
Ctrl_R_1	62870176	9430526400	93.33	0.01	99.24	96.83	42.79
Ctrl_R_2	90321230	1.35E+10	94.36	0.01	99.21	96.71	42.92
Ctrl_R_3	62241628	9336244200	92.87	0.01	99.25	97.08	42.73

Ctrl_R_4	71862790	1.08E+10	93.31	0.01	99.18	96.93	42.84
Cu_L_1	70797042	1.06E+10	95.1	0.01	99.29	96.93	44.35
Cu_L_2	69167184	1.04E+10	95.99	0.01	99.24	96.87	43.99
Cu_L_3	68421544	1.03E+10	94.94	0.01	99.35	97.33	44.14
Cu_L_4	71665334	1.08E+10	92.41	0.01	99.18	96.71	44.12
Cu_R_1	77629496	1.16E+10	93.94	0.01	99.24	96.8	42.85
Cu_R_2	80897562	1.21E+10	92.86	0.01	99.33	97.14	42.79
Cu_R_3	70411430	1.06E+10	94.77	0.01	99.32	97.16	42.85
Cu_R_4	109427160	1.64E+10	96.18	0.01	99.08	97.42	42.78
Fe_L_1	65562622	9834393300	93.51	0.01	99.18	96.62	43.97
Fe_L_2	87708572	1.32E+10	95.26	0.01	99.23	96.8	43.17
Fe_L_3	64272606	9640890900	94.68	0.01	99.34	97.25	44.09
Fe_L_4	59713256	8956988400	94.13	0.01	99.24	96.78	44.13
Fe_R_1	72308496	1.08E+10	92.83	0.01	99.16	96.6	42.51
Fe_R_2	69791438	1.05E+10	94.85	0.01	99.23	96.77	42.38
Fe_R_3	70872508	1.06E+10	93.55	0.01	99.26	97.28	42.77
Fe_R_4	78402052	1.18E+10	96.94	0.01	99.11	97.5	42.68
K_L_1	73411174	1.10E+10	93.1	0.01	99.21	96.75	43.06
K_L_2	67419332	1.01E+10	94.06	0.01	99.04	96.08	43.86
K_L_3	79174282	1.19E+10	92.91	0.01	99.32	97.15	43.49
K_L_4	87831850	1.32E+10	94.91	0.01	99.29	97.19	43.48
K_R_1	66217644	9932646600	93.25	0.01	99.22	96.7	42.54
K_R_2	101568554	1.52E+10	95.5	0.01	99.25	96.78	42.84
K_R_3	61142754	9171413100	94.33	0.01	99.28	97.12	42.7
K_R_4	64900872	9735130800	93.77	0.01	98.91	96.96	43.03
Mg_L_1	77383814	1.16E+10	95.65	0.01	99.27	96.85	43.16
Mg_L_2	71399302	1.07E+10	95.03	0.01	99.27	96.93	44

Mg_L_3	61107052	9166057800	95.06	0.01	99.27	97.04	42.96
Mg_L_4	63703392	9555508800	93.24	0.01	98.86	96.83	43.21
Mg_R_1	61645174	9246776100	94.93	0.01	99.26	96.75	43.09
Mg_R_2	81280894	1.22E+10	94.49	0.01	99.2	96.78	42.84
Mg_R_3	67431114	1.01E+10	93.79	0.01	99.27	97	42.55
Mg_R_4	73451500	1.10E+10	95.94	0.01	99.27	97.03	42.43
Mn_L_1	75601024	1.13E+10	92.64	0.01	99.19	96.66	43.62
Mn_L_2	70132718	1.05E+10	95.63	0.01	99.2	96.68	43.8
Mn_L_3	61125246	9168786900	88.04	0.01	99.29	97.17	43.66
Mn_L_4	63608712	9541306800	94.78	0.01	99.24	96.81	43.56
Mn_R_1	64744340	9711651000	93.16	0.01	99.25	96.84	42.86
Mn_R_2	84960472	1.27E+10	93.59	0.01	99.35	97.27	42.58
Mn_R_3	62949040	9442356000	94.72	0.01	99.27	97.11	42.92
Mn_R_4	80979062	1.21E+10	95.74	0.01	99.07	97.34	42.71
Mo_L_1	80046964	1.20E+10	92.52	0.01	99.2	96.76	44.07
Mo_L_2	98470664	1.48E+10	95.8	0.01	99.23	96.76	44.3
Mo_L_3	74119012	1.11E+10	94.07	0.01	99.29	97.16	44.06
Mo_L_4	63021134	9453170100	90.9	0.01	97.1	92.4	44.38
Mo_R_1	72467216	1.09E+10	93.88	0.01	99.2	96.8	42.64
Mo_R_2	61301446	9195216900	89.35	0.01	98.87	96.9	42.59
Mo_R_3	64114466	9617169900	93.6	0.01	99.3	97.19	42.83
Mo_R_4	70647906	1.06E+10	95.61	0.01	98.98	97.07	42.83
N_L_1	102146332	1.53E+10	92.39	0.01	99.23	96.77	43.19
N_L_2	77580508	1.16E+10	93.4	0.01	99.07	96.23	43.04
N_L_3	73917908	1.11E+10	92.34	0.01	99.32	97.11	43.15
N_L_4	71921970	1.08E+10	93.32	0.01	99.27	97.16	43.13
N_R_1	78477952	1.18E+10	97.41	0.01	99.29	97	41.9

N_R_2	65730978	9859646700	94.6	0.01	99.24	96.88	42.65
N_R_3	69819284	1.05E+10	93.49	0.01	99.31	97.23	42.89
N_R_4	70341606	1.06E+10	92.85	0.01	98.94	97.01	42.75
P_L_1	74965124	1.12E+10	92.92	0.01	99.17	96.68	43.54
P_L_2	82234320	1.23E+10	94.24	0.01	99.22	96.7	43.96
P_L_3	82734142	1.24E+10	92.19	0.01	99.32	97.15	44.3
P_L_4	64157408	9623611200	93.94	0.01	99.25	97.05	44.41
P_R_1	77418934	1.16E+10	92.34	0.01	99.19	96.67	42.79
P_R_2	82323336	1.23E+10	93.46	0.01	99.2	96.73	43.02
P_R_3	66972324	1.00E+10	93.9	0.01	99.28	97.15	42.91
P_R_4	61232316	9184847400	93.25	0.01	98.9	96.91	42.77
S_L_1	62440030	9366004500	92.74	0.01	99.22	96.63	43.89
S_L_2	109380400	1.64E+10	95.55	0.01	99.28	96.88	44
S_L_3	71706502	1.08E+10	93.47	0.01	99.3	97.24	44.17
S_L_4	67883298	1.02E+10	92.41	0.01	98.87	96.83	44.23
S_R_1	77418686	1.16E+10	94.51	0.01	99.25	96.84	42.66
S_R_2	106524352	1.60E+10	93.82	0.01	99.19	96.74	42.93
S_R_3	68080976	1.02E+10	93.85	0.01	99.28	97.26	42.86
S_R_4	80481172	1.21E+10	97.13	0.01	99.08	97.41	42.92
Zn_L_1	68080342	1.02E+10	93.57	0.01	99.21	96.65	44.36
Zn_L_2	65622508	9843376200	96.2	0.01	99.24	96.78	43.85
Zn_L_3	69726670	1.05E+10	94.51	0.01	99.36	97.33	43.68
Zn_L_4	66592608	9988891200	93.25	0.01	99.19	96.73	43.74
Zn_R_1	72968266	1.09E+10	93.7	0.01	99.22	96.75	42.81
Zn_R_2	72266356	1.08E+10	94.7	0.01	99.15	96.57	42.64
Zn_R_3	68136314	1.02E+10	94.04	0.01	99.33	97.24	42.95
Zn_R_4	106484738	1.60E+10	97.48	0.01	99.15	97.56	42.76

Supplemental Table S2. Quality control metrics for all RNA-seq replicates

Sample	% Duplication	% GC	Total Sequences (millions)
B_L_1	77.7236633	44	32.361932
B_L_2	71.21017792	44	38.771366
B_L_3	72.55563708	44	33.495489
B_L_4	67.73129522	44	30.589683
B_R_1	63.95828773	43	33.879404
B_R_2	70.37060896	42	33.893406
B_R_3	70.72001397	42.5	32.918996
B_R_4	63.60163941	42	34.399559
Ca_L_1	74.5587573	44	40.526804
Ca_L_2	77.09445058	44	42.050503
Ca_L_3	77.21294962	44	35.704047
Ca_L_4	71.50665932	44	36.125728
Ca_R_1	78.041859	42	40.830921
Ca_R_2	70.38367022	42	42.044255
Ca_R_3	73.99220117	42	35.42154
Ca_R_4	74.59790128	42	35.810458
Ctrl_L_1	80.00729998	43.5	36.461523
Ctrl_L_2	72.8281729	43	37.248638
Ctrl_L_3	78.09626272	44	43.970549
Ctrl_L_4	72.90942665	44	34.954298
Ctrl_R_1	71.31513472	42	31.435088
Ctrl_R_2	76.44760532	43	45.160615
Ctrl_R_3	69.48119224	42	31.120814
Ctrl_R_4	63.96999742	42.5	35.931395
Cu_L_1	79.02625182	44	35.398521
Cu_L_2	75.05523236	44	34.583592
Cu_L_3	76.82474331	44	34.210772
Cu_L_4	67.57407627	44	35.832667
Cu_R_1	66.92613066	42.5	38.814748
Cu_R_2	75.63749729	42	40.448781
Cu_R_3	64.85825437	42	35.205715
Cu_R_4	79.57196915	42	54.71358
Fe_L_1	69.35536381	44	32.781311
Fe_L_2	73.47971508	43	43.854286
Fe_L_3	74.20684801	44	32.136303
Fe_L_4	68.8323744	44	29.856628

Fe_R_1	81.38330883	42	36.154248
Fe_R_2	66.89510329	42	34.895719
Fe_R_3	68.01666844	42	35.436254
Fe_R_4	86.75880952	42	39.201026
K_L_1	74.65406386	43	36.705587
K_L_2	70.39144958	43	33.709666
K_L_3	71.2359081	43	39.587141
K_L_4	71.24591997	43	43.915925
K_R_1	66.28727116	42	33.108822
K_R_2	76.75348437	42	50.784277
K_R_3	63.79768339	42	30.571377
K_R_4	71.40520628	43	32.450436
Mg_L_1	66.4533189	43	38.691907
Mg_L_2	72.5958487	44	35.699651
Mg_L_3	65.99797759	43	30.553526
Mg_L_4	64.98261908	43	31.851696
Mg_R_1	70.12482173	43	30.822587
Mg_R_2	66.45830751	42	40.640447
Mg_R_3	69.78426739	42	33.715557
Mg_R_4	76.59281438	42	36.72575
Mn_L_1	72.12680226	43	37.800512
Mn_L_2	67.30670646	43	35.066359
Mn_L_3	91.81140484	43	30.562623
Mn_L_4	66.51927661	43	31.804356
Mn_R_1	69.21635835	43	32.37217
Mn_R_2	73.51846857	42	42.480236
Mn_R_3	66.16477678	43	31.47452
Mn_R_4	75.88296236	42	40.489531
Mo_L_1	74.08048093	44	40.023482
Mo_L_2	79.0448347	44	49.235332
Mo_L_3	75.5428874	44	37.059506
Mo_L_4	63.73206906	45	31.510567
Mo_R_1	74.15100499	42	36.233608
Mo_R_2	67.09789113	42	30.650723
Mo_R_3	70.55826484	42	32.057233
Mo_R_4	69.81805677	42	35.323953
N_L_1	80.27189634	43	51.073166
N_L_2	87.0746857	43	38.790254
N_L_3	71.88519387	43	36.958954

N_L_4	68.81638682	43	35.960985
N_R_1	58.84193561	41	39.238976
N_R_2	65.87493493	42	32.865489
N_R_3	68.17389365	43	34.909642
N_R_4	61.4526272	42	35.170803
P_L_1	82.87102325	43	37.482562
P_L_2	76.06913413	44	41.11716
P_L_3	76.7007755	44	41.367071
P_L_4	71.51522552	44	32.078704
P_R_1	77.03248386	42.5	38.709467
P_R_2	73.79264388	43	41.161668
P_R_3	71.68616775	43	33.486162
P_R_4	62.29939636	42	30.616158
S_L_1	73.53107043	44	31.220015
S_L_2	82.13140012	44	54.6902
S_L_3	68.74761048	44	35.853251
S_L_4	71.69765178	44	33.941649
S_R_1	68.38069544	42	38.709343
S_R_2	77.39106298	43	53.262176
S_R_3	68.50376164	43	34.040488
S_R_4	78.01052815	42.5	40.240586
Zn_L_1	68.69618213	44	34.040171
Zn_L_2	75.55704074	43.5	32.811254
Zn_L_3	75.17312067	43	34.863335
Zn_L_4	67.97378207	43	33.296304
Zn_R_1	69.99744426	42	36.484133
Zn_R_2	69.91405389	42	36.133178
Zn_R_3	72.32112372	43	34.068157
Zn_R_4	79.47357414	42	53.242369

Supplemental Table S3. Mapping statistics for paired-end reads mapped to the QQ74 v2.0 reference

Sample	Total Reads	Mapped	Mapped %	Properly Paired	Properly Paired %
B_L_1	83903350	78130456	93.12	46928240	78.8
B_L_2	86744726	81689940	94.17	61541844	84.19
B_L_3	84664367	79424890	93.81	51220616	82.34

B_L_4	67706796	62671805	92.56	45459884	80.55
B_R_1	72375662	64926118	89.71	46323064	74.42
B_R_2	70894981	64857876	91.48	51585850	84.94
B_R_3	68291971	63947358	93.64	51457182	85.52
B_R_4	74550287	69789924	93.61	54169422	83.96
Ca_L_1	96643458	88883443	91.97	55717270	74.67
Ca_L_2	96063915	89267805	92.93	63446420	80.83
Ca_L_3	86759635	81242488	93.64	54443088	83.08
Ca_L_4	79977866	73911063	92.41	53948932	81.44
Ca_R_1	86357865	81392668	94.25	63736308	87.24
Ca_R_2	92096932	83722573	90.91	61750828	78.74
Ca_R_3	83935065	79717562	94.98	58464914	86.61
Ca_R_4	84950575	79970719	94.14	55004038	80.83
Ctrl_L_1	92644064	86680507	93.56	54999484	80.79
Ctrl_L_2	93259654	86111698	92.34	54495438	78.47
Ctrl_L_3	105872770	98376590	92.92	64531514	79.35
Ctrl_L_4	84865267	79949330	94.21	53423870	82.41
Ctrl_R_1	66479670	60811959	91.47	45307618	78.15
Ctrl_R_2	99641533	93862187	94.2	71578956	85.25
Ctrl_R_3	65729811	59985544	91.26	44759478	78.33
Ctrl_R_4	76282676	70601328	92.55	55068910	83.11
Cu_L_1	97709905	91777646	93.93	52066382	78.54
Cu_L_2	88558916	82821795	93.52	52846972	81.01
Cu_L_3	83385549	78097654	93.66	53323526	83.03
Cu_L_4	79035596	73339467	92.79	54486876	83.33
Cu_R_1	82216052	73623377	89.55	51496878	71.64
Cu_R_2	86017607	78766832	91.57	58349550	78.44

Cu_R_3	77288962	71044065	91.92	54181664	82.2
Cu_R_4	118600926	110552416	93.21	83049768	79.96
Fe_L_1	71956082	68055128	94.58	51788148	85.74
Fe_L_2	96389176	88790844	92.12	64929450	79
Fe_L_3	77090158	71903826	93.27	49087026	81.63
Fe_L_4	66127162	61423734	92.89	44797984	80.88
Fe_R_1	77607232	70441990	90.77	51582770	77.97
Fe_R_2	75901668	69313050	91.32	52743004	80.94
Fe_R_3	80537388	75330666	93.54	55446096	84.46
Fe_R_4	88459337	84202238	95.19	62826590	83.86
K_L_1	96529393	89818636	93.05	51605060	76.61
K_L_2	78861776	72073428	91.39	48277562	77.81
K_L_3	93312684	86936903	93.17	59211072	81.35
K_L_4	107715789	101447263	94.18	69169180	84.11
K_R_1	68830458	61891333	89.92	44440984	73.03
K_R_2	109992061	101954863	92.69	79476744	83.23
K_R_3	67422782	63577237	94.3	50195900	88.12
K_R_4	68918842	65819934	95.5	53312378	88.69
Mg_L_1	86340216	79240893	91.78	54994732	75.52
Mg_L_2	79616182	74133058	93.11	55275532	82.67
Mg_L_3	66500366	61297308	92.18	45694694	79.79
Mg_L_4	71245723	65409335	91.81	46779306	79.83
Mg_R_1	65222967	57855807	88.7	40579232	70.37
Mg_R_2	87587086	81655153	93.23	63079578	83.45
Mg_R_3	73859509	68012938	92.08	51710182	82.74
Mg_R_4	79490172	75391746	94.84	58961154	84.95
Mn_L_1	113221586	107961257	95.35	56673886	82.08

Mn_L_2	78733881	73044300	92.77	53560740	81.32
Mn_L_3	72397811	68525931	94.65	47314180	87.36
Mn_L_4	79706309	74567871	93.55	48093000	80.99
Mn_R_1	68490832	61747819	90.15	43962482	73.81
Mn_R_2	90900107	81870889	90.07	60016200	76.3
Mn_R_3	68545958	64014356	93.39	49544106	84.18
Mn_R_4	88333462	82981339	93.94	63474928	82.89
Mo_L_1	93007633	84184475	90.51	54909390	75.12
Mo_L_2	110093600	103635658	94.13	76438460	82.49
Mo_L_3	85747834	79837632	93.11	54990562	79.94
Mo_L_4	69486799	64095165	92.24	41798450	78.34
Mo_R_1	77411077	69657426	89.98	50376268	75.09
Mo_R_2	65544609	60503189	92.31	46106764	84.64
Mo_R_3	69256573	65122219	94.03	51154904	86.18
Mo_R_4	76952012	71349214	92.72	52239604	78.51
N_L_1	125476706	116812914	93.1	74586746	80.09
N_L_2	114262633	107503623	94.08	56710418	79.81
N_L_3	79838933	73086782	91.54	55190860	81.63
N_L_4	84369751	79886276	94.69	56880842	85.74
N_R_1	85944800	78782777	91.67	60236998	80.24
N_R_2	68795486	62995594	91.57	49563970	80.94
N_R_3	72758660	66618697	91.56	51315344	79.51
N_R_4	72678249	67690246	93.14	54453224	84.3
P_L_1	104330988	97945338	93.88	53892754	78.5
P_L_2	88959398	81647193	91.78	62051478	81.33
P_L_3	90863495	82745600	91.07	58970442	78.07
P_L_4	73115032	69382666	94.9	51716086	86.95

P_R_1	81043142	72775164	89.8	52456880	74.39
P_R_2	86977173	81036049	93.17	63271524	83.42
P_R_3	70410315	64725002	91.93	49236546	79.31
P_R_4	63652951	58964439	92.63	46819988	83.05
S_L_1	80669372	74923280	92.88	43000832	75.29
S_L_2	141557799	132778474	93.8	85065962	82.65
S_L_3	77598665	73202550	94.33	56749086	85.48
S_L_4	77153284	72023583	93.35	51273080	82.85
S_R_1	83339639	74675081	89.6	52440880	72.75
S_R_2	113736718	105913841	93.12	81918258	83.18
S_R_3	71661346	66841742	93.27	53559908	84.69
S_R_4	87526926	84132374	96.12	68489826	89.01
Zn_L_1	75169167	72274060	96.15	55718878	88.87
Zn_L_2	73862618	69572728	94.19	51424402	82.85
Zn_L_3	80868411	75600683	93.49	53083916	81.43
Zn_L_4	75198519	69694766	92.68	49288846	80.55
Zn_R_1	80404267	73121211	90.94	52684920	78.21
Zn_R_2	80954642	75459795	93.21	55827688	83.05
Zn_R_3	77100091	71914109	93.27	52630706	83.01
Zn_R_4	130258561	122155095	93.78	84180538	82.22

Supplemental Table S4. Domain-level taxonomic classification summary of RNA-seq reads across all samples. Percentage of sequencing reads assigned to each taxonomic domain for each of the 104 samples, as determined by Kraken2 taxonomic classification. Columns report the percentage of reads that were unclassified, or classified as Eukaryota, Bacteria, Viruses, or Archaea, relative to total reads per sample. This summary was used to screen for potential microbial or viral contamination prior to downstream expression analyses.

Sample	% Reads Unclassified	% Reads Eukaryote	% Reads Bacterial	% Reads Viral	% Reads Archaean
B_L_1	45.24	54.06	0.47	0	0.01

B_L_2	38.03	61.18	0.53	0.01	0.02
B_L_3	42.6	56.64	0.53	0	0.02
B_L_4	36.79	62.42	0.53	0.01	0.02
B_R_1	42.41	56.93	0.41	0.01	0.02
B_R_2	49.94	49.2	0.63	0.01	0.03
B_R_3	45.61	53.71	0.46	0.01	0.02
B_R_4	49.06	50.26	0.47	0.01	0.02
Ca_L_1	40.25	59.01	0.48	0	0.02
Ca_L_2	38.53	60.64	0.57	0.01	0.02
Ca_L_3	41.58	57.62	0.57	0.01	0.02
Ca_L_4	38.74	60.46	0.54	0.01	0.02
Ca_R_1	47.11	52.19	0.47	0.01	0.02
Ca_R_2	48.34	50.98	0.44	0.01	0.02
Ca_R_3	48.26	51.08	0.43	0.01	0.02
Ca_R_4	49.09	50.26	0.41	0.01	0.02
Ctrl_L_1	42.7	56.6	0.46	0	0.02
Ctrl_L_2	44.15	55.14	0.48	0	0.02
Ctrl_L_3	38.74	60.5	0.51	0	0.02
Ctrl_L_4	39.75	59.5	0.51	0	0.02
Ctrl_R_1	44.64	54.69	0.42	0.01	0.02
Ctrl_R_2	46.68	52.49	0.58	0.01	0.02
Ctrl_R_3	45.3	54	0.45	0.01	0.02
Ctrl_R_4	45.87	53.44	0.45	0.01	0.02
Cu_L_1	42.04	57.23	0.48	0	0.02
Cu_L_2	43.52	55.79	0.47	0	0.01
Cu_L_3	40.45	58.78	0.54	0.01	0.02
Cu_L_4	37.66	61.5	0.6	0.01	0.02
Cu_R_1	42.15	57.2	0.39	0.01	0.01
Cu_R_2	43.81	55.53	0.42	0.01	0.02
Cu_R_3	46.02	53.28	0.46	0.01	0.02
Cu_R_4	44.14	55.21	0.42	0.01	0.02
Fe_L_1	38.17	61.04	0.53	0.01	0.02
Fe_L_2	42.16	57.08	0.49	0.01	0.02

Fe_L_3	40.3	58.93	0.53	0.01	0.02
Fe_L_4	36.84	62.33	0.57	0	0.02
Fe_R_1	45.53	53.82	0.4	0.01	0.02
Fe_R_2	51.54	47.77	0.46	0.01	0.02
Fe_R_3	47.48	51.85	0.44	0.01	0.02
Fe_R_4	45.51	53.87	0.38	0.01	0.02
K_L_1	51.45	47.96	0.37	0.01	0.01
K_L_2	40.63	58.61	0.52	0	0.02
K_L_3	43.9	55.42	0.44	0.01	0.02
K_L_4	45.1	54.23	0.43	0.01	0.02
K_R_1	44.9	54.43	0.42	0.01	0.02
K_R_2	46.52	52.75	0.48	0.01	0.02
K_R_3	48.96	50.33	0.47	0.01	0.02
K_R_4	45.18	54.05	0.5	0.01	0.02
Mg_L_1	43.38	55.95	0.42	0.01	0.01
Mg_L_2	37.31	61.89	0.55	0.01	0.02
Mg_L_3	42.2	57.09	0.46	0.02	0.01
Mg_L_4	44.61	54.73	0.42	0.01	0.01
Mg_R_1	42.47	56.82	0.44	0.02	0.02
Mg_R_2	44.65	54.71	0.41	0.01	0.02
Mg_R_3	48.35	50.97	0.45	0.01	0.02
Mg_R_4	48.86	50.51	0.41	0.01	0.02
Mn_L_1	48.86	50.51	0.4	0	0.01
Mn_L_2	38.51	60.7	0.53	0.01	0.02
Mn_L_3	44.82	54.33	0.64	0.01	0.02
Mn_L_4	43.7	55.56	0.48	0.01	0.02
Mn_R_1	42.8	56.54	0.4	0.01	0.02
Mn_R_2	46.49	52.83	0.44	0.01	0.02
Mn_R_3	45.99	53.34	0.44	0.01	0.02
Mn_R_4	45.8	53.53	0.43	0.01	0.02
Mo_L_1	39.42	59.83	0.5	0	0.02
Mo_L_2	35.83	63.37	0.54	0	0.02
Mo_L_3	37.85	61.36	0.54	0.01	0.02

Mo_L_4	40.61	58.65	0.49	0.01	0.02
Mo_R_1	46.25	53.09	0.41	0.01	0.02
Mo_R_2	50.14	49.11	0.52	0.01	0.02
Mo_R_3	46.38	52.94	0.45	0.01	0.02
Mo_R_4	44.51	54.84	0.41	0.01	0.02
N_L_1	48.66	50.69	0.43	0.01	0.01
N_L_2	55.55	43.88	0.38	0.01	0.01
N_L_3	45.69	53.59	0.5	0	0.02
N_L_4	47.78	51.56	0.44	0	0.02
N_R_1	49.72	49.58	0.45	0.01	0.02
N_R_2	46.79	52.52	0.45	0.01	0.02
N_R_3	43.46	55.87	0.42	0.01	0.02
N_R_4	44.37	54.95	0.43	0.01	0.02
P_L_1	48.05	51.31	0.4	0	0.02
P_L_2	36.62	62.58	0.56	0.01	0.02
P_L_3	36.56	62.61	0.58	0	0.02
P_L_4	37.22	61.93	0.59	0	0.02
P_R_1	43.62	55.68	0.43	0.01	0.02
P_R_2	42.98	56.33	0.45	0.01	0.02
P_R_3	43.12	56.2	0.43	0.01	0.02
P_R_4	45.09	54.23	0.44	0.01	0.02
S_L_1	43.71	55.6	0.45	0	0.02
S_L_2	42.77	56.47	0.53	0	0.02
S_L_3	37.67	61.55	0.54	0	0.02
S_L_4	38.23	60.99	0.54	0	0.02
S_R_1	43.53	55.8	0.41	0.01	0.02
S_R_2	44.63	54.71	0.43	0.01	0.02
S_R_3	46.93	52.33	0.51	0.01	0.02
S_R_4	44	55.34	0.42	0.01	0.02
Zn_L_1	36.26	62.85	0.59	0.01	0.02
Zn_L_2	37.6	61.61	0.53	0.01	0.02
Zn_L_3	39.9	59.35	0.51	0.01	0.02
Zn_L_4	39.85	59.4	0.51	0.01	0.02

Zn_R_1	45.64	53.73	0.4	0.01	0.02
Zn_R_2	48.13	51.26	0.4	0.01	0.02
Zn_R_3	46.1	53.22	0.44	0.01	0.02
Zn_R_4	47.8	51.58	0.4	0.01	0.02

Supplemental Table S5. Randomized complete block design for the hydroponics experiment involving four blocks with the positive control and all 12 macro- and micronutrient deficiencies.

**I**	**II**	**III**	**IV**
Ctrl	B	S	K
N	Ca	Mo	Ctrl
P	Fe	Zn	N
K	Mn	Cu	Mn
S	Mo	N	Ca
Ca	Ctrl	Mg	Fe
Mg	N	P	B
Zn	S	B	Mg
Fe	Zn	Mn	P
Mn	P	Ctrl	Cu
Cu	K	Ca	Zn
B	Cu	Fe	S
Mo	Mg	K	Mo

Supplemental Table S6. RNA sequencing resources and data availability. Sequencing data is deposited in the NCBI Sequence Read Archive (SRA) under BioProject ID PRJNA1484747. BioSample ID, Nutrient deficiency (treatment), tissue and replication and corresponding NCBI SRA accession ID are provided. All samples were sequenced using Illumina paired-end (2 X 150 bp) sequencing.

**BioProject ID**	**BioSample ID**	**Nutrient Deficiency**	**Tissue**	**Replication**	**NCBI SRA #**
PRJNA1484747	SAMN61254393	Boron	Leaf	Rep1	SRR39419353
PRJNA1484747	SAMN61254393	Boron	Leaf	Rep2	SRR39419352
PRJNA1484747	SAMN61254393	Boron	Leaf	Rep3	SRR39419337
PRJNA1484747	SAMN61254393	Boron	Leaf	Rep4	SRR39419326

PRJNA1484747	SAMN61254393	Boron	Root	Rep1	SRR39419315
PRJNA1484747	SAMN61254393	Boron	Root	Rep2	SRR39419304
PRJNA1484747	SAMN61254393	Boron	Root	Rep3	SRR39419293
PRJNA1484747	SAMN61254393	Boron	Root	Rep4	SRR39419282
PRJNA1484747	SAMN61254393	Calcium	Leaf	Rep1	SRR39419271
PRJNA1484747	SAMN61254393	Calcium	Leaf	Rep2	SRR39419260
PRJNA1484747	SAMN61254393	Calcium	Leaf	Rep3	SRR39419351
PRJNA1484747	SAMN61254393	Calcium	Leaf	Rep4	SRR39419346
PRJNA1484747	SAMN61254393	Calcium	Root	Rep1	SRR39419345
PRJNA1484747	SAMN61254393	Calcium	Root	Rep2	SRR39419344
PRJNA1484747	SAMN61254393	Calcium	Root	Rep3	SRR39419343
PRJNA1484747	SAMN61254393	Calcium	Root	Rep4	SRR39419342
PRJNA1484747	SAMN61254393	Control	Leaf	Rep1	SRR39419341
PRJNA1484747	SAMN61254393	Control	Leaf	Rep2	SRR39419340
PRJNA1484747	SAMN61254393	Control	Leaf	Rep3	SRR39419339
PRJNA1484747	SAMN61254393	Control	Leaf	Rep4	SRR39419338
PRJNA1484747	SAMN61254393	Control	Root	Rep1	SRR39419336
PRJNA1484747	SAMN61254393	Control	Root	Rep2	SRR39419335
PRJNA1484747	SAMN61254393	Control	Root	Rep3	SRR39419334
PRJNA1484747	SAMN61254393	Control	Root	Rep4	SRR39419333
PRJNA1484747	SAMN61254393	Copper	Leaf	Rep1	SRR39419332
PRJNA1484747	SAMN61254393	Copper	Leaf	Rep2	SRR39419331
PRJNA1484747	SAMN61254393	Copper	Leaf	Rep3	SRR39419330
PRJNA1484747	SAMN61254393	Copper	Leaf	Rep4	SRR39419329
PRJNA1484747	SAMN61254393	Copper	Root	Rep1	SRR39419328
PRJNA1484747	SAMN61254393	Copper	Root	Rep2	SRR39419327
PRJNA1484747	SAMN61254393	Copper	Root	Rep3	SRR39419325
PRJNA1484747	SAMN61254393	Copper	Root	Rep4	SRR39419324
PRJNA1484747	SAMN61254393	Iron	Leaf	Rep1	SRR39419323

PRJNA1484747	SAMN61254393	Iron	Leaf	Rep2	SRR39419322
PRJNA1484747	SAMN61254393	Iron	Leaf	Rep3	SRR39419321
PRJNA1484747	SAMN61254393	Iron	Leaf	Rep4	SRR39419320
PRJNA1484747	SAMN61254393	Iron	Root	Rep1	SRR39419319
PRJNA1484747	SAMN61254393	Iron	Root	Rep2	SRR39419318
PRJNA1484747	SAMN61254393	Iron	Root	Rep3	SRR39419317
PRJNA1484747	SAMN61254393	Iron	Root	Rep4	SRR39419316
PRJNA1484747	SAMN61254393	Potassium	Leaf	Rep1	SRR39419314
PRJNA1484747	SAMN61254393	Potassium	Leaf	Rep2	SRR39419313
PRJNA1484747	SAMN61254393	Potassium	Leaf	Rep3	SRR39419312
PRJNA1484747	SAMN61254393	Potassium	Leaf	Rep4	SRR39419311
PRJNA1484747	SAMN61254393	Potassium	Root	Rep1	SRR39419310
PRJNA1484747	SAMN61254393	Potassium	Root	Rep2	SRR39419309
PRJNA1484747	SAMN61254393	Potassium	Root	Rep3	SRR39419308
PRJNA1484747	SAMN61254393	Potassium	Root	Rep4	SRR39419307
PRJNA1484747	SAMN61254393	Magnesium	Leaf	Rep1	SRR39419306
PRJNA1484747	SAMN61254393	Magnesium	Leaf	Rep2	SRR39419305
PRJNA1484747	SAMN61254393	Magnesium	Leaf	Rep3	SRR39419303
PRJNA1484747	SAMN61254393	Magnesium	Leaf	Rep4	SRR39419302
PRJNA1484747	SAMN61254393	Magnesium	Root	Rep1	SRR39419301
PRJNA1484747	SAMN61254393	Magnesium	Root	Rep2	SRR39419300
PRJNA1484747	SAMN61254393	Magnesium	Root	Rep3	SRR39419299
PRJNA1484747	SAMN61254393	Magnesium	Root	Rep4	SRR39419298
PRJNA1484747	SAMN61254393	Manganese	Leaf	Rep1	SRR39419297
PRJNA1484747	SAMN61254393	Manganese	Leaf	Rep2	SRR39419296
PRJNA1484747	SAMN61254393	Manganese	Leaf	Rep3	SRR39419295
PRJNA1484747	SAMN61254393	Manganese	Leaf	Rep4	SRR39419294
PRJNA1484747	SAMN61254393	Manganese	Root	Rep1	SRR39419292
PRJNA1484747	SAMN61254393	Manganese	Root	Rep2	SRR39419291

PRJNA1484747	SAMN61254393	Manganese	Root	Rep3	SRR39419290
PRJNA1484747	SAMN61254393	Manganese	Root	Rep4	SRR39419289
PRJNA1484747	SAMN61254393	Molybdenum	Leaf	Rep1	SRR39419288
PRJNA1484747	SAMN61254393	Molybdenum	Leaf	Rep2	SRR39419287
PRJNA1484747	SAMN61254393	Molybdenum	Leaf	Rep3	SRR39419286
PRJNA1484747	SAMN61254393	Molybdenum	Leaf	Rep4	SRR39419285
PRJNA1484747	SAMN61254393	Molybdenum	Root	Rep1	SRR39419284
PRJNA1484747	SAMN61254393	Molybdenum	Root	Rep2	SRR39419283
PRJNA1484747	SAMN61254393	Molybdenum	Root	Rep3	SRR39419281
PRJNA1484747	SAMN61254393	Molybdenum	Root	Rep4	SRR39419280
PRJNA1484747	SAMN61254393	Nitrogen	Leaf	Rep1	SRR39419279
PRJNA1484747	SAMN61254393	Nitrogen	Leaf	Rep2	SRR39419278
PRJNA1484747	SAMN61254393	Nitrogen	Leaf	Rep3	SRR39419277
PRJNA1484747	SAMN61254393	Nitrogen	Leaf	Rep4	SRR39419276
PRJNA1484747	SAMN61254393	Nitrogen	Root	Rep1	SRR39419275
PRJNA1484747	SAMN61254393	Nitrogen	Root	Rep2	SRR39419274
PRJNA1484747	SAMN61254393	Nitrogen	Root	Rep3	SRR39419273
PRJNA1484747	SAMN61254393	Nitrogen	Root	Rep4	SRR39419272
PRJNA1484747	SAMN61254393	Phosphorus	Leaf	Rep1	SRR39419270
PRJNA1484747	SAMN61254393	Phosphorus	Leaf	Rep2	SRR39419269
PRJNA1484747	SAMN61254393	Phosphorus	Leaf	Rep3	SRR39419268
PRJNA1484747	SAMN61254393	Phosphorus	Leaf	Rep4	SRR39419267
PRJNA1484747	SAMN61254393	Phosphorus	Root	Rep1	SRR39419266
PRJNA1484747	SAMN61254393	Phosphorus	Root	Rep2	SRR39419265
PRJNA1484747	SAMN61254393	Phosphorus	Root	Rep3	SRR39419264
PRJNA1484747	SAMN61254393	Phosphorus	Root	Rep4	SRR39419263
PRJNA1484747	SAMN61254393	Sulfur	Leaf	Rep1	SRR39419262
PRJNA1484747	SAMN61254393	Sulfur	Leaf	Rep2	SRR39419261
PRJNA1484747	SAMN61254393	Sulfur	Leaf	Rep3	SRR39419259

PRJNA1484747	SAMN61254393	Sulfur	Leaf	Rep4	SRR39419258
PRJNA1484747	SAMN61254393	Sulfur	Root	Rep1	SRR39419257
PRJNA1484747	SAMN61254393	Sulfur	Root	Rep2	SRR39419256
PRJNA1484747	SAMN61254393	Sulfur	Root	Rep3	SRR39419255
PRJNA1484747	SAMN61254393	Sulfur	Root	Rep4	SRR39419254
PRJNA1484747	SAMN61254393	Zinc	Leaf	Rep1	SRR39419253
PRJNA1484747	SAMN61254393	Zinc	Leaf	Rep2	SRR39419252
PRJNA1484747	SAMN61254393	Zinc	Leaf	Rep3	SRR39419251
PRJNA1484747	SAMN61254393	Zinc	Leaf	Rep4	SRR39419250
PRJNA1484747	SAMN61254393	Zinc	Root	Rep1	SRR39419350
PRJNA1484747	SAMN61254393	Zinc	Root	Rep2	SRR39419349
PRJNA1484747	SAMN61254393	Zinc	Root	Rep3	SRR39419348
PRJNA1484747	SAMN61254393	Zinc	Root	Rep4	SRR39419347

Supplemental Figures

Supplemental Figure S1. Pearson correlation heatmap of all 104 replicates across the positive control and deficiency treatments. Each cell displays the Pearson correlation coefficient between two samples, with the color scale ranging from blue (lower correlation, $r = 0.6$) to red (high correlation, $r = 1.0$), with tissue of origin indicated by the color bar (green = leaf, brown = root).

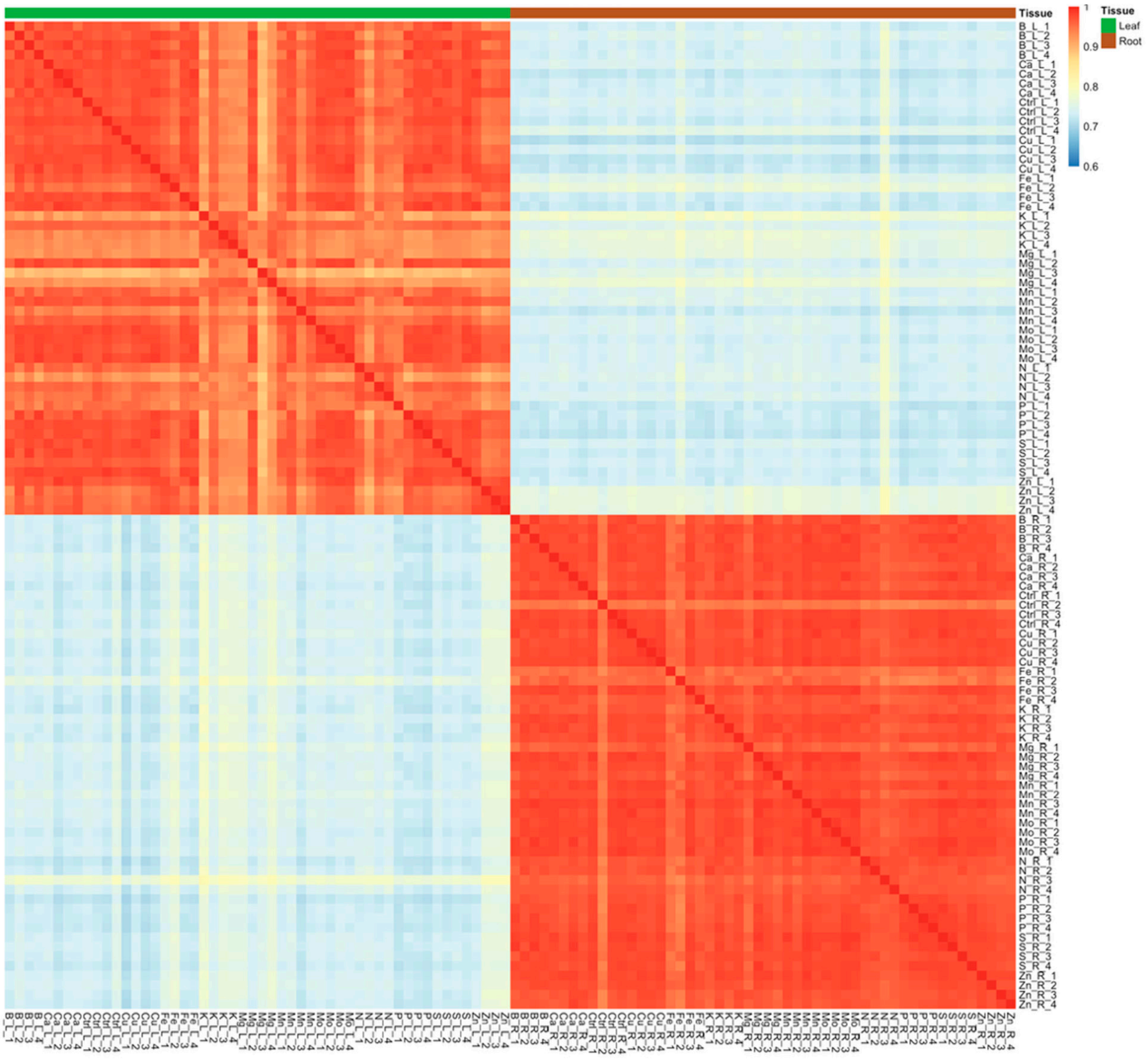

Supplemental Figure S2. Pairwise Pearson correlation heatmaps of individual leaf biological replicates for each treatment condition. Each cell displays the Pearson correlation coefficient between two samples, with color scale ranging from blue (lower correlation, $r = 0.9$) to red (higher correlation, $r = 1.0$). Samples are hierarchically clustered within each panel. (A) Control, (B) nitrogen deficiency, (C) phosphorus deficiency, (D) potassium deficiency, (E) sulfur deficiency, (F) calcium deficiency, (G) magnesium deficiency, (H) zinc deficiency, (I) iron deficiency, (J) manganese deficiency, (K) boron deficiency, (L) copper deficiency, and (M) molybdenum deficiency.

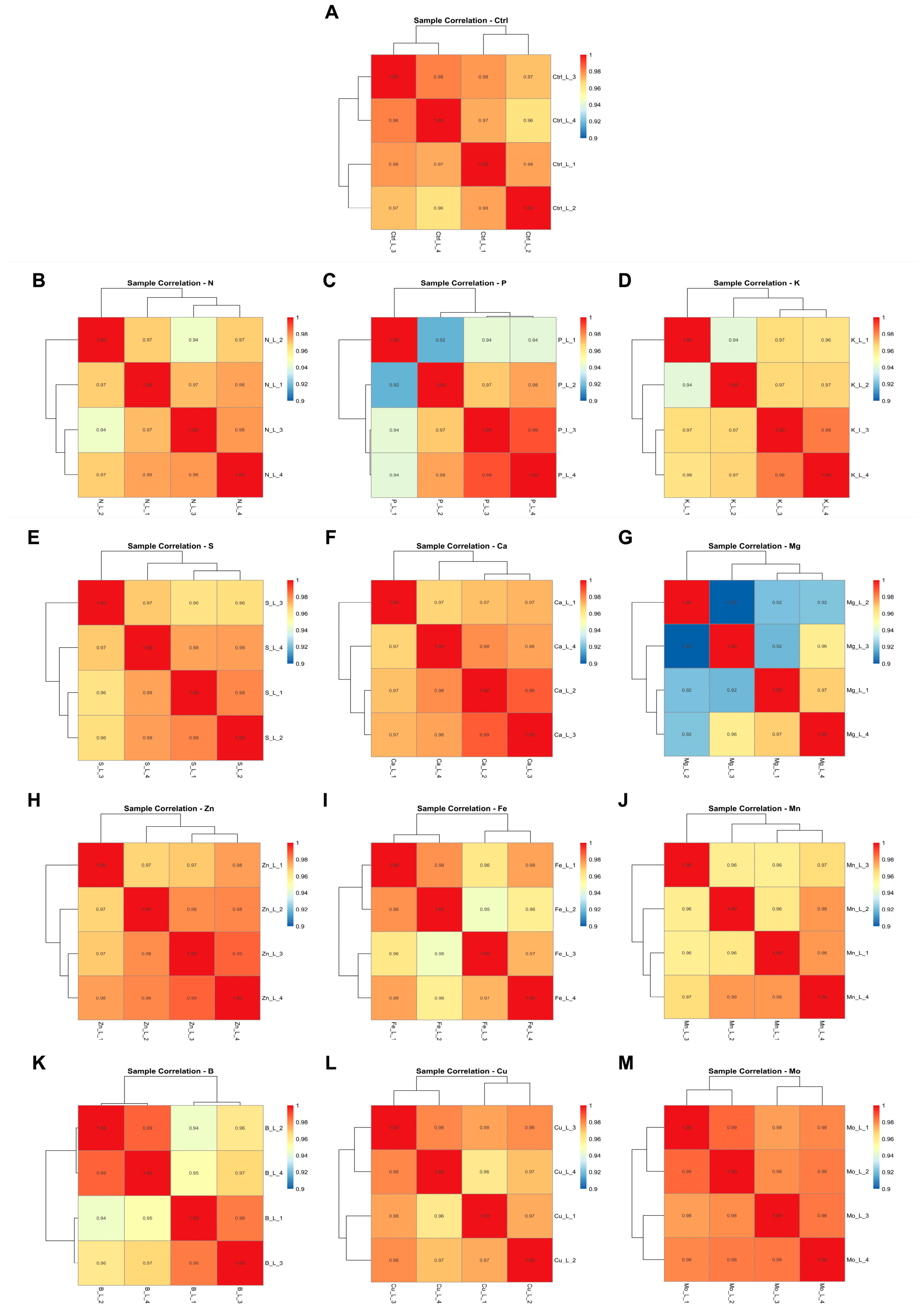
A
Sample Correlation - Ctrl
B
Sample Correlation - N
C
Sample Correlation - P
D
Sample Correlation - K
E
Sample Correlation - S
F
Sample Correlation - Ca
G
Sample Correlation - Mg
H
Sample Correlation - Zn
I
Sample Correlation - Fe
J
Sample Correlation - Mn
K
Sample Correlation - B
L
Sample Correlation - Cu
M
Sample Correlation - Mo

Supplemental Figure S3. Pairwise Pearson correlation heatmaps of individual root biological replicates for each treatment condition. Each cell displays the Pearson correlation coefficient between two samples, with color scale ranging from blue (lower correlation, $r = 0.9$) to red (higher correlation, $r = 1.0$). Samples are hierarchically clustered within each panel. (A) Control, (B) nitrogen deficiency, (C) phosphorus deficiency, (D) potassium deficiency, (E) sulfur deficiency, (F) calcium deficiency, (G) magnesium deficiency, (H) zinc deficiency, (I) iron deficiency, (J) manganese deficiency, (K) boron deficiency, (L) copper deficiency, and (M) molybdenum deficiency.

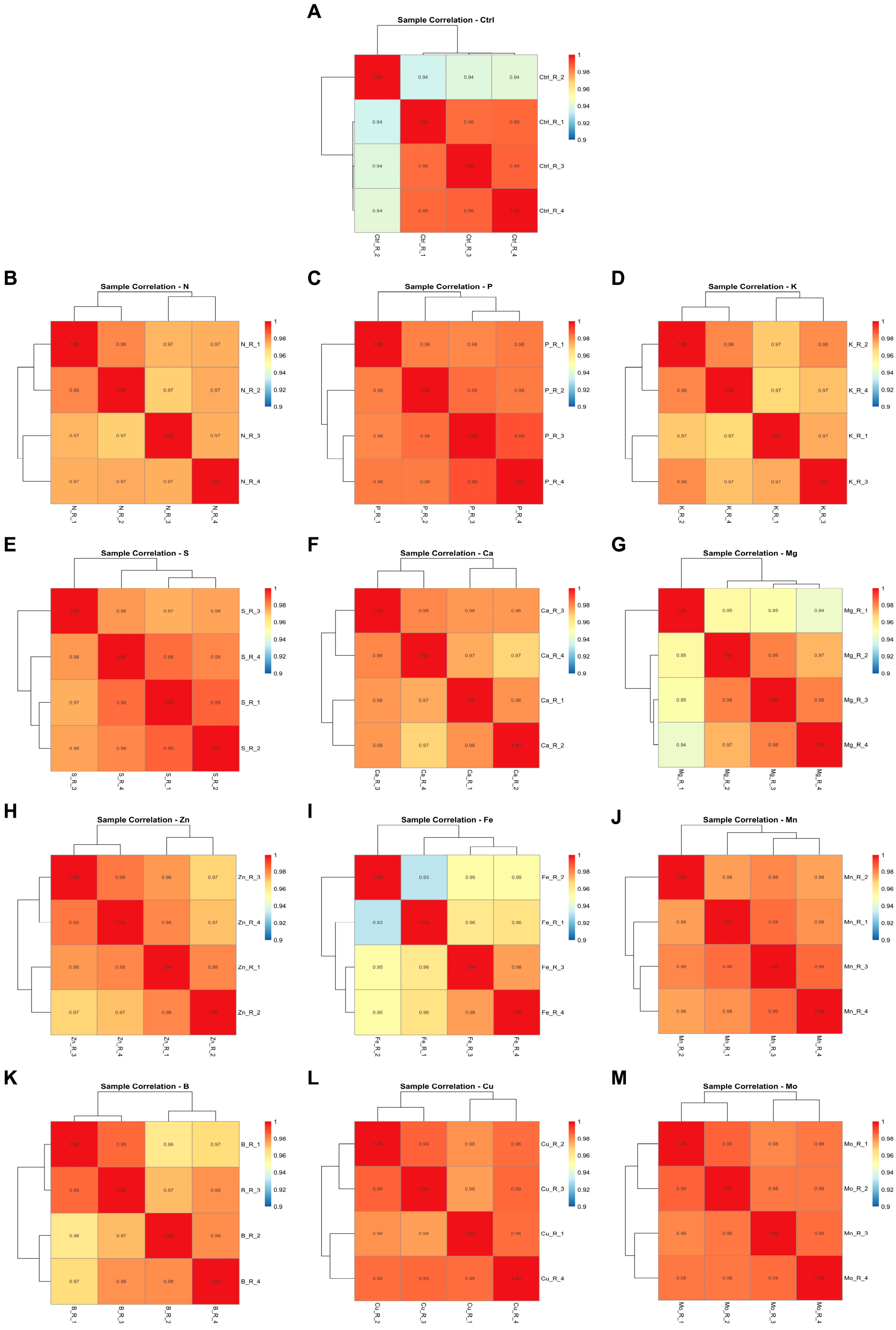
A
Sample Correlation - Ctrl
Ctrl_R_2
Ctrl_R_1
Ctrl_R_3
Ctrl_R_4
1
0.98
0.96
0.94
0.92
0.9
B
Sample Correlation - N
N_R_1
N_R_2
N_R_3
N_R_4
C
Sample Correlation - P
P_R_1
P_R_2
P_R_3
P_R_4
D
Sample Correlation - K
K_R_2
K_R_4
K_R_1
K_R_3
E
Sample Correlation - S
S_R_3
S_R_4
S_R_1
S_R_2
F
Sample Correlation - Ca
Ca_R_3
Ca_R_4
Ca_R_1
Ca_R_2
G
Sample Correlation - Mg
Mg_R_1
Mg_R_2
Mg_R_3
Mg_R_4
H
Sample Correlation - Zn
Zn_R_3
Zn_R_4
Zn_R_1
Zn_R_2
I
Sample Correlation - Fe
Fe_R_2
Fe_R_1
Fe_R_3
Fe_R_4
J
Sample Correlation - Mn
Mn_R_2
Mn_R_1
Mn_R_3
Mn_R_4
K
Sample Correlation - B
B_R_1
B_R_3
B_R_2
B_R_4
L
Sample Correlation - Cu
Cu_R_2
Cu_R_3
Cu_R_1
Cu_R_4
M
Sample Correlation - Mo
Mo_R_1
Mo_R_2
Mo_R_3
Mo_R_4

Supplemental Figure S4. Leave-one-out replicate robustness across all nutrient-deficiency comparisons. Heatmap showing the mean Pearson correlation coefficient (*r*) between log2 fold-change estimates from the full-replicate DESeq2 analysis and each leave-one-out iteration (replicate excluded one at a time), for all twelve nutrient-deficiency comparisons in leaf and root tissue. Correlations are shown across all tested genes ("all_genes") and within three confidence tiers defined by the adjusted p-value of the full-replicate analysis (p-adj < 0.05, p-adj < 0.01, p-adj < 0.001), with values averaged across all leave-one-out iterations for each nutrient-tissue comparison. Higher correlation values (pink/red) indicate greater stability of fold-change estimates regardless of which replicate was excluded, while lower values (white/blue) indicate greater sensitivity to individual replicates. Rows are hierarchically clustered by correlation profile (Euclidean distance, average linkage); columns are held in fixed order from least to most stringent confidence tier. Tissue of origin is indicated by the color bar at left (green = leaf, brown = root).

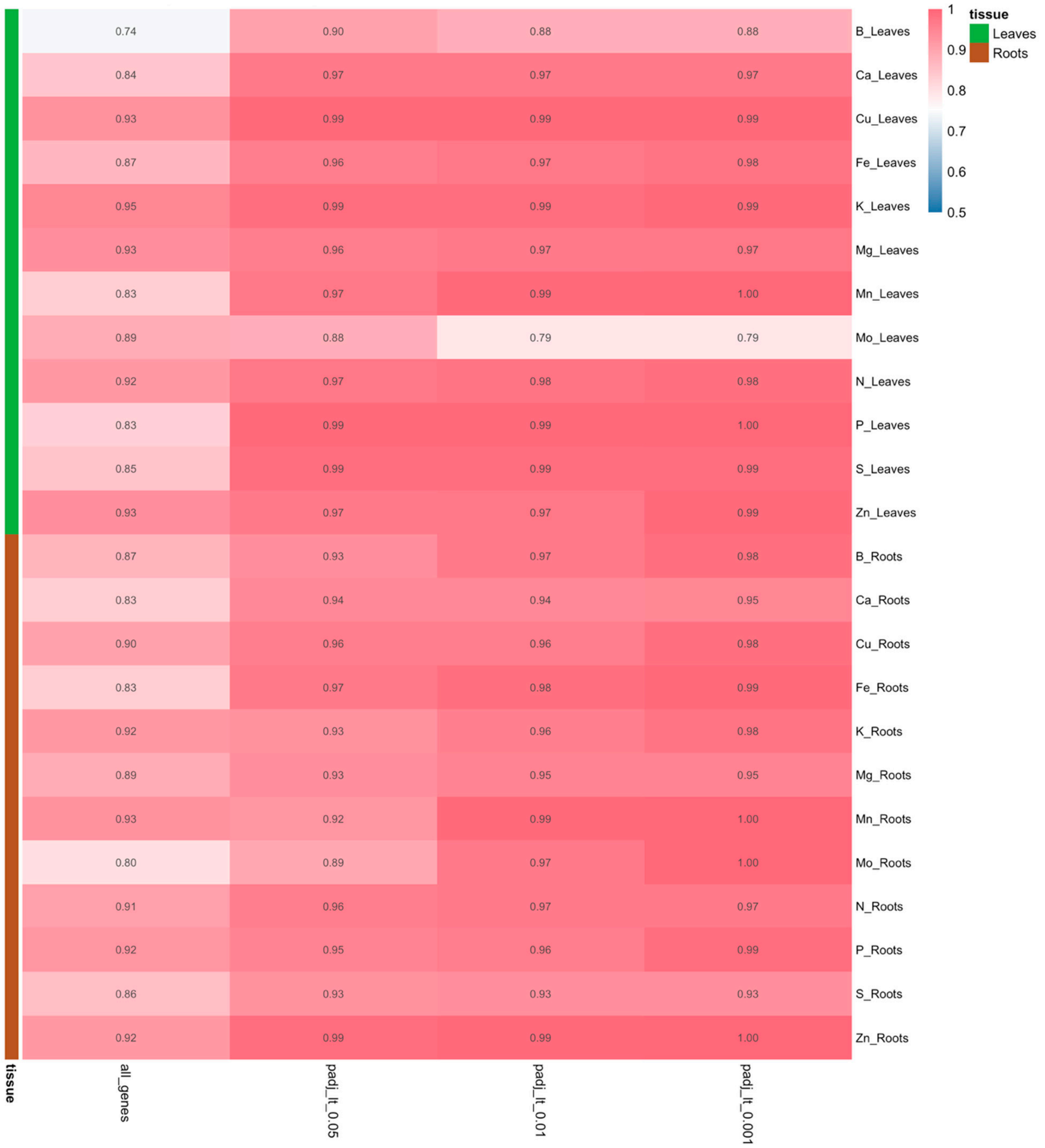

Supplemental Figure S5. Volcano plots displaying differential gene expression in leaf samples for each nutrient deficiency treatment relative to the control. Each point represents a gene, plotted as log_2FC on the x-axis against negative log10 adjusted *p*-value on the y-axis. Genes are colored by significance category: blue indicates significantly downregulated genes (*p*-adj < 0.05 and $log_2FC < -1$), yellow-green indicates significantly upregulated genes (*p*-adj < 0.05 and $log_2FC > 1$), and gray indicates non-significant genes. Dashed vertical lines indicate log_2FC thresholds of ±1 and the dashed horizontal line indicate the significance threshold (*p*-adj = 0.05).

(A) nitrogen, (B) phosphorus, (C) potassium, (D) sulfur, (E) calcium, (F) magnesium, (G) zinc, (H) iron, (I) manganese, (J) boron, (K) copper, and (L) molybdenum deficiency.

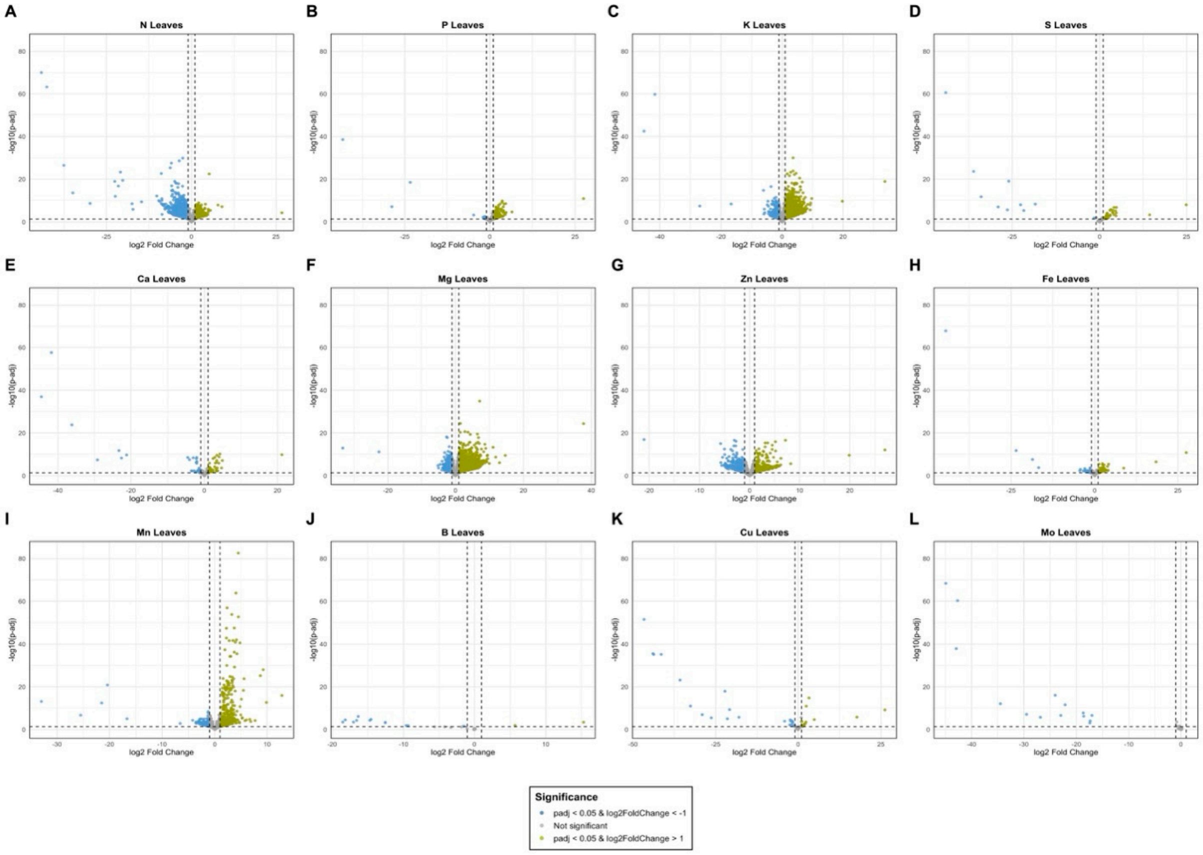

Supplemental Figure S6. Volcano plots displaying differential gene expression in root samples for each nutrient deficiency treatment relative to the control. Each point represents a gene, plotted as log_2FC on the x-axis against negative log10 adjusted p-value on the y-axis. Genes are colored by significance category: blue indicates significantly downregulated genes (*p*-adj < 0.05 and $log_2FC < -1$), yellow-green indicates significantly upregulated genes (p-adj < 0.05 and $log_2FC > 1$), and gray indicates non-significant genes. Dashed vertical lines indicate log_2FC thresholds of ±1 and the dashed horizontal line indicate the significance threshold (padj = 0.05). (A) nitrogen, (B) phosphorus, (C) potassium, (D) sulfur, (E) calcium, (F) magnesium, (G) zinc, (H) iron, (I) manganese, (J) boron, (K) copper, and (L) molybdenum deficiency.

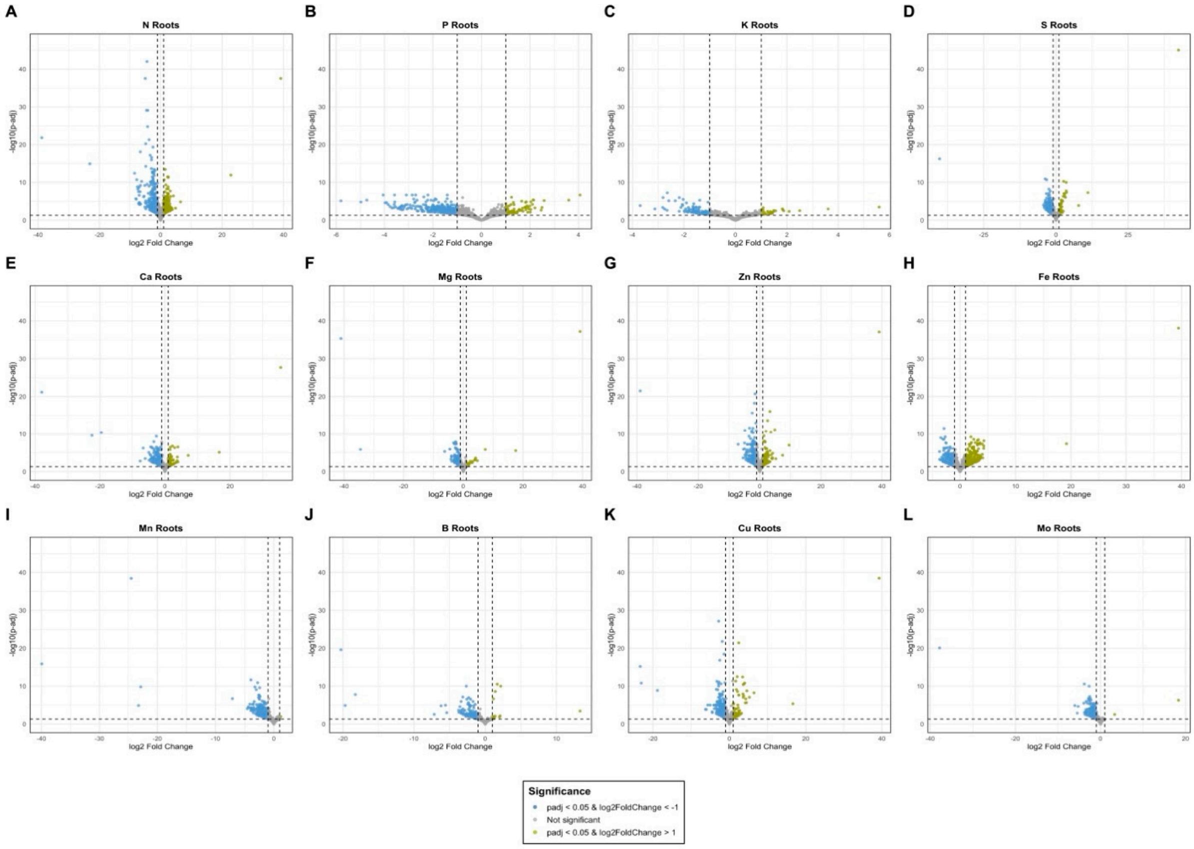

Supplemental Figure S7. Condition-specific differentially expressed genes (DEGs) under nitrogen deficiency in leaves and roots. (A) Stacked bar chart showing the total number of DEGs in each tissue, partitioned into unique (green) and shared (yellow) fractions. (B) Treemap of enriched and semantically clustered biological process (BP) Gene Ontology terms for nitrogen-specific leaf DEGs, with cluster size proportional to the number of genes associated with each term. (C) Treemap of enriched and semantically clustered molecular function (MF) Gene Ontology terms for nitrogen-specific leaf DEGs. (D) Treemap of enriched and semantically clustered BP Gene Ontology terms for nitrogen-specific root DEGs. (E) Treemap of enriched and semantically clustered MF Gene Ontology terms for nitrogen-specific root DEGs. GO term enrichment was performed using TRAPID and semantic clustering was performed using rrvgo.

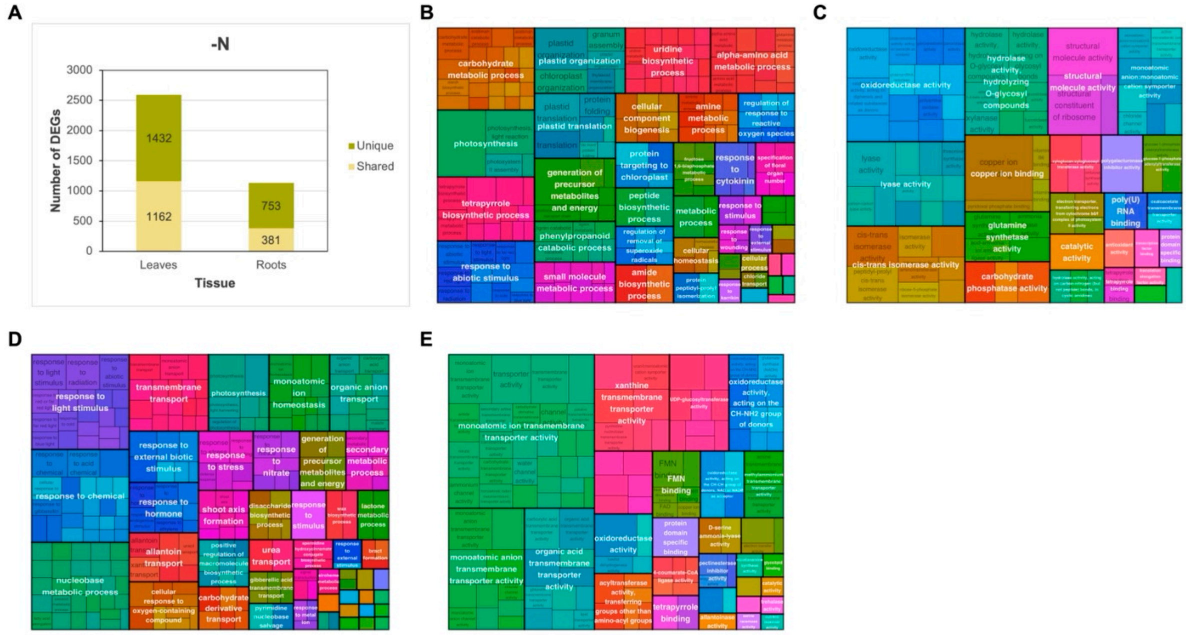

Supplemental Figure S8. Gene set enrichment analysis (GSEA) results for nitrogen-deficient leaf and root transcriptomes. (A) Treemap of enriched and semantically clustered biological process (BP) Gene Ontology terms in leaves. (B) Treemap of enriched and semantically clustered molecular function (MF) Gene Ontology terms in leaves. (C) Dot plot of enriched KEGG pathways in leaves, faceted by direction of enrichment, with dot size proportional to the number of genes in each pathway and dot color indicating adjusted p-value. (D) Pathview rendering of the photosynthesis KEGG pathway in leaves, with genes colored by log_2FC on a blue to red scale, where blue indicates downregulation and red indicates upregulation. (E) Treemap of enriched and semantically clustered biological process (BP) Gene Ontology terms in roots. (F) Treemap of enriched and semantically clustered molecular function (MF) Gene Ontology terms in roots. (G) Dot plot of enriched KEGG pathways in roots, faceted by direction of enrichment, with dot size proportional to the number of genes in each pathway and dot color indicating adjusted p-value. (H) Pathview rendering of the photosynthesis KEGG pathway in roots. GSEA was performed using the GSEA function in clusterProfiler with Benjamini-Hochberg correction and a p-value cutoff of 0.05. Pathway visualization was performed using Pathview.

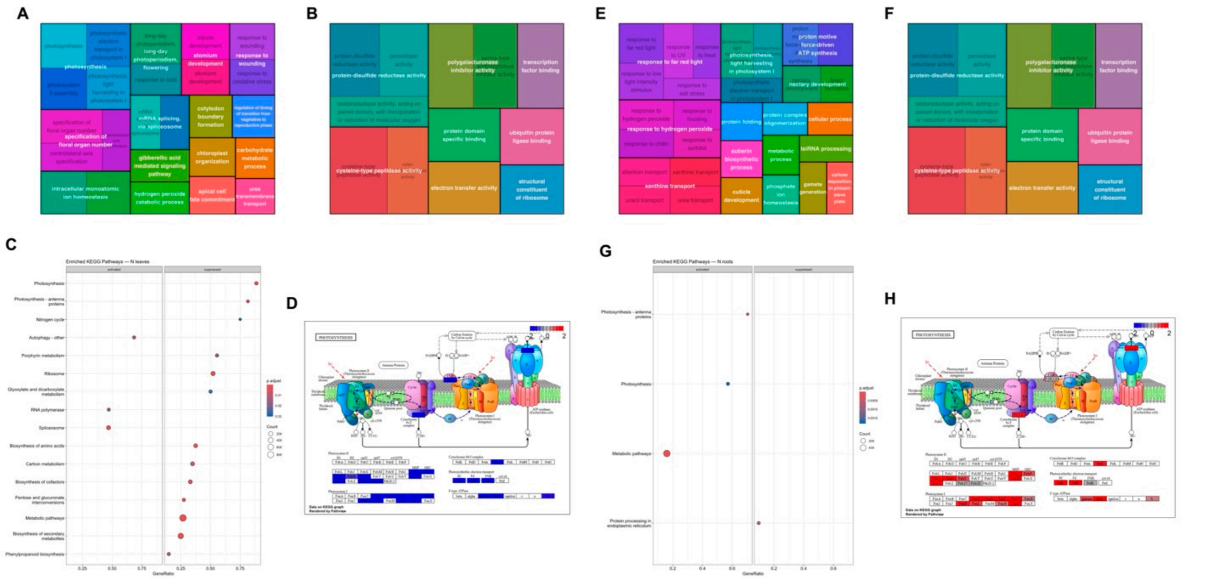

Supplemental Figure S9. Partial least squares discriminant analysis (PLS-DA) of nitrogen-deficient and control transcriptomes in leaves and roots. (A) Individuals plot for leaf samples, with each point representing a biological replicate colored by treatment group and 95% confidence ellipses shown for each group. The percentage of variance explained by each component is indicated on the respective axis. (B) Table of the top 20 genes ranked by variable importance in projection (VIP) score on component 1 for leaf samples, with gene name and VIP score shown. (C) Treemap of enriched and semantically clustered biological process (BP) Gene Ontology terms for leaf transcripts with a VIP score ≥ 1.5. (D) Treemap of enriched and semantically clustered molecular function (MF) Gene Ontology terms for leaf transcripts with a VIP score ≥ 1.5. (E) Individuals plot for root samples, with each point representing a biological replicate colored by treatment group and 95% confidence ellipses shown for each group. (F) Table of the top 20 genes ranked by VIP score on component 1 for root samples. (G) Treemap of enriched and semantically clustered BP Gene Ontology terms for root transcripts with a VIP score ≥ 1.5. (H) Treemap of enriched and semantically clustered MF Gene Ontology terms for root transcripts with a VIP score ≥ 1.5. PLS-DA was performed using the mixOmics package in R and GO term enrichment was performed using TRAPID with semantic clustering by rrvgo.

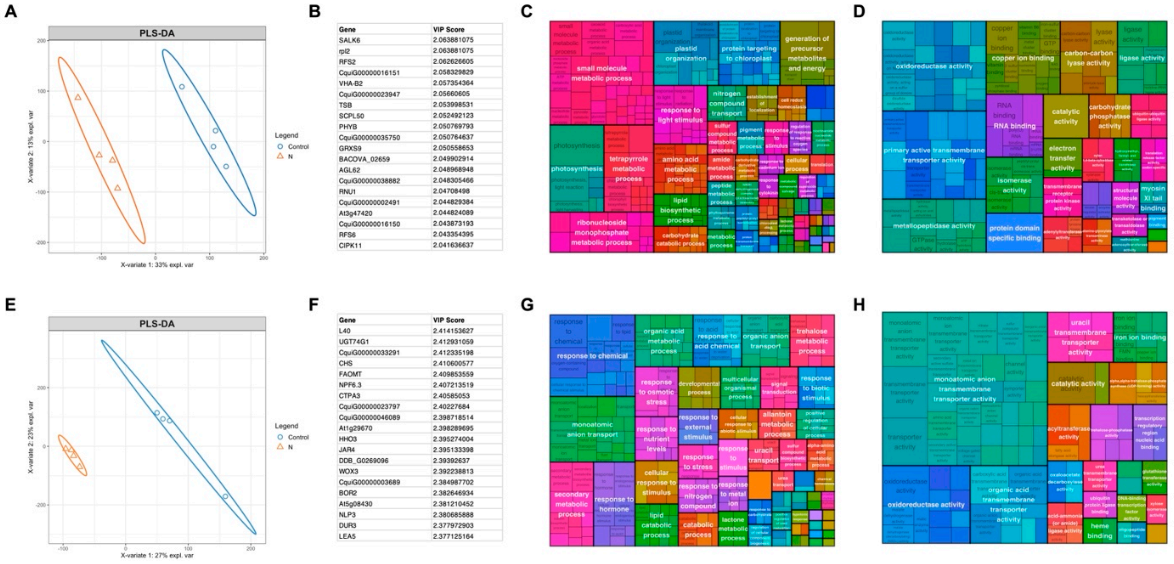

Gene	VIP Score
SALK6	2.063881075
rpl2	2.063881075
RFS2	2.062626605
CquiG00000016151	2.058329829
VHA-B2	2.057354364
CquiG00000023947	2.05660605
TSB	2.053998531
SCPL50	2.052492123
PHYB	2.050769793
CquiG00000035750	2.050764637
GRXS9	2.050558653
BACOVA_02659	2.049902914
AGL62	2.048968948
CquiG00000038882	2.048305466
RNU1	2.04708498
CquiG00000002491	2.044829384
At3g47420	2.044824089
CquiG00000016150	2.043873193
RFS6	2.043354395
CIPK11	2.041636637

Gene	VIP Score
L40	2.414153627
UGT74G1	2.412931059
CquiG00000033291	2.412335198
CHS	2.410600577
FAOMT	2.409853559
NPF6.3	2.407213519
CTPA3	2.40585053
CquiG00000023797	2.40227684
CquiG00000046089	2.398718514
At1g29670	2.398289695
HHO3	2.395274004
JAR4	2.395133398
DDB_G0269096	2.39392637
WOX3	2.392238813
CquiG00000003689	2.384987702
BOR2	2.382646934
At5g08430	2.381210452
NLP3	2.380685888
DUR3	2.377972903
LEA5	2.377125164

Supplemental Figure S10. Co-expression module characterization for module eigengenes strongly correlated with nitrogen deficiency in leaves and roots. (A) Treemap of enriched and semantically clustered biological process (BP) Gene Ontology terms for ME20 in leaves. (B) Treemap of enriched and semantically clustered molecular function (MF) Gene Ontology terms for ME20 in leaves. (C) Network plot of the top 20 hub genes in ME20 identified by intramodular connectivity, with node labels indicating gene names. (D) Treemap of enriched and semantically clustered BP Gene Ontology terms for ME6 in roots. (E) Treemap of enriched and semantically clustered MF Gene Ontology terms for ME6 in roots. (F) Network plot of the top 20 hub genes in ME6 identified by intramodular connectivity. GO term enrichment was performed using TRAPID and semantic clustering was performed using rrvgo. Module eigengenes were identified using the WGCNA package in R.

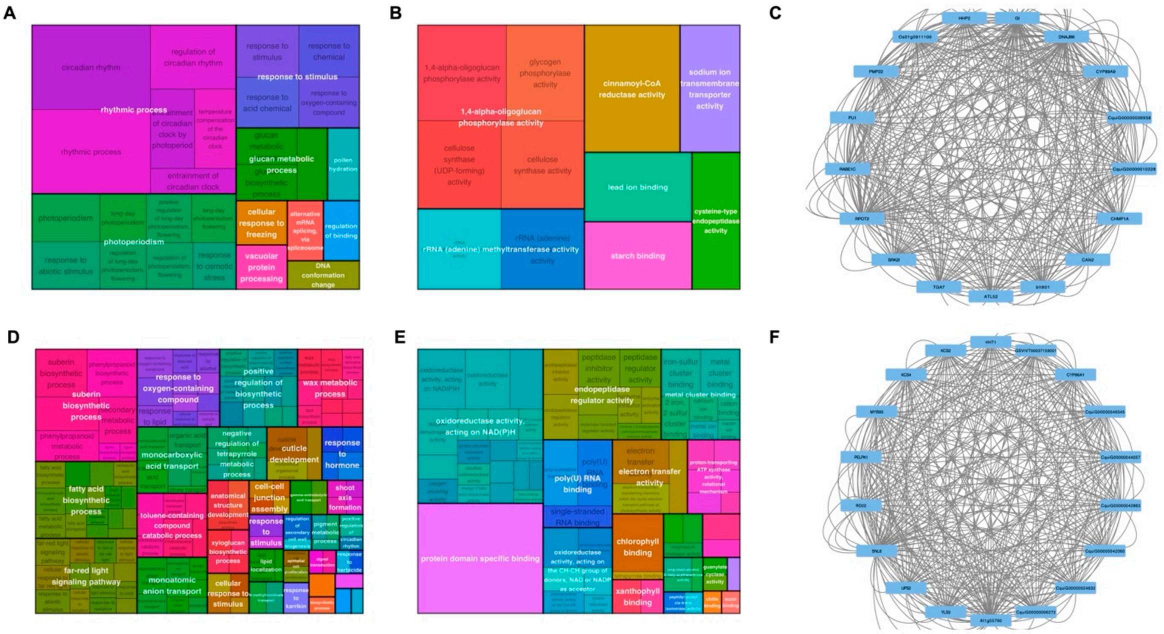

Supplemental Figure S11. Condition-specific differentially expressed genes (DEGs) under phosphorus deficiency in leaves and roots. (A) Stacked bar chart showing the total number of DEGs in each tissue, partitioned into unique (green) and shared (yellow) fractions. (B) Treemap of enriched and semantically clustered biological process (BP) Gene Ontology terms for phosphorus-specific leaf DEGs, with cluster size proportional to the number of genes associated with each term. (C) Treemap of enriched and semantically clustered molecular function (MF) Gene Ontology terms for phosphorus-specific leaf DEGs. (D) Treemap of enriched and semantically clustered BP Gene Ontology terms for phosphorus-specific root DEGs. (E) Treemap of enriched and semantically clustered MF Gene Ontology terms for phosphorus-specific root DEGs. GO term enrichment was performed using TRAPID and semantic clustering was performed using rrvgo.

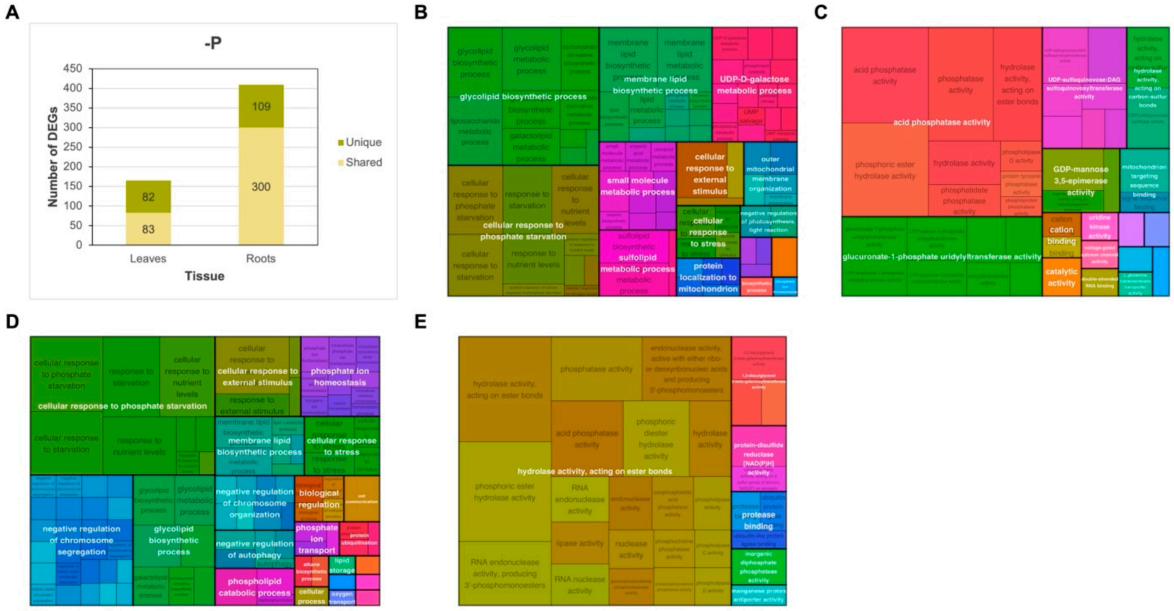

Supplemental Figure S12. Gene set enrichment analysis (GSEA) results for phosphorus-deficient leaf and root transcriptomes. (A) Treemap of enriched and semantically clustered biological process (BP) Gene Ontology terms in leaves. (B) Treemap of enriched and semantically clustered molecular function (MF) Gene Ontology terms in leaves. (C) Dot plot of enriched KEGG pathways in leaves, faceted by direction of enrichment, with dot size proportional to the number of genes in each pathway and dot color indicating adjusted p-value. (D) Pathview rendering of the sulfoquinovose metabolism KEGG pathway in leaves, with genes colored by log_2FC on a blue to red scale, where blue indicates downregulation and red indicates upregulation. (E) Treemap of enriched and semantically clustered biological process (BP) Gene Ontology terms in roots. (F) Treemap of enriched and semantically clustered molecular function (MF) Gene Ontology terms in roots. (G) Dot plot of enriched KEGG pathways in roots, faceted by direction of enrichment, with dot size proportional to the number of genes in each pathway and dot color indicating adjusted p-value. (H) Pathview rendering of the ribosome KEGG pathway in roots. GSEA was performed using the GSEA function in clusterProfiler with Benjamini-Hochberg correction and a p-value cutoff of 0.05. Pathway visualization was performed using Pathview.

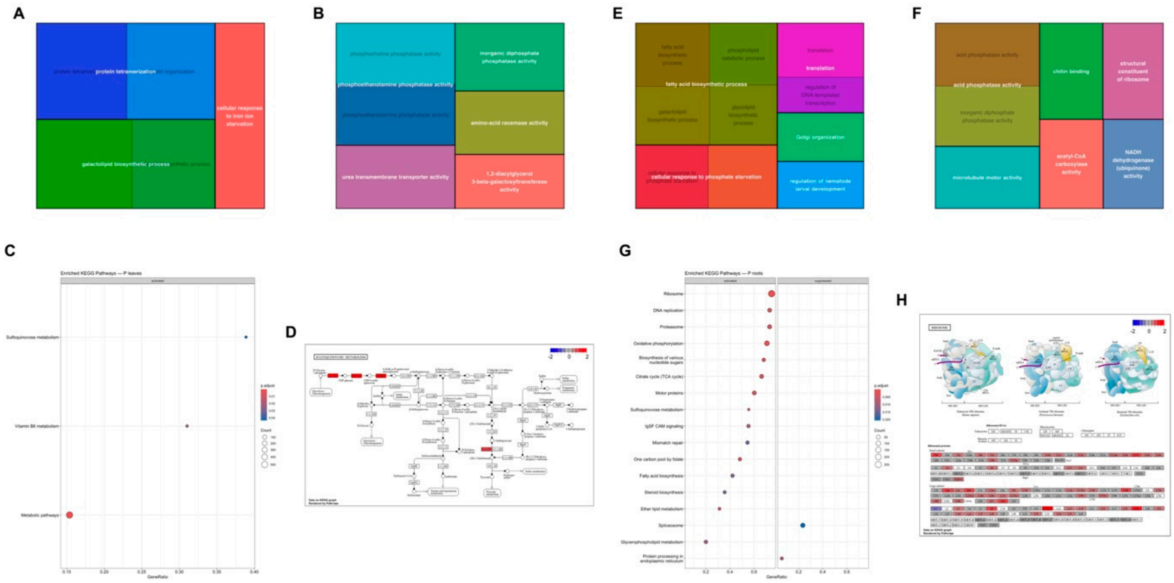

Supplemental Figure S13. Partial least squares discriminant analysis (PLS-DA) of phosphorus-deficient and control transcriptomes in leaves and roots. (A) Individuals plot for leaf samples, with each point representing a biological replicate colored by treatment group and 95% confidence ellipses shown for each group. The percentage of variance explained by each component is indicated on the respective axis. (B) Table of the top 20 genes ranked by variable importance in projection (VIP) score on component 1 for leaf samples, with gene name and VIP score shown. (C) Treemap of enriched and semantically clustered biological process (BP) Gene Ontology terms for leaf transcripts with a VIP score ≥ 1.5. (D) Treemap of enriched and semantically clustered molecular function (MF) Gene Ontology terms for leaf transcripts with a VIP score ≥ 1.5. (E) Individuals plot for root samples, with each point representing a biological replicate colored by treatment group and 95% confidence ellipses shown for each group. (F) Table of the top 20 genes ranked by VIP score on component 1 for root samples. (G) Treemap of enriched and semantically clustered BP Gene Ontology terms for root transcripts with a VIP score ≥ 1.5. (H) Treemap of enriched and semantically clustered MF Gene Ontology terms for root transcripts with a VIP score ≥ 1.5. PLS-DA was performed using the mixOmics package in R and GO term enrichment was performed using TRAPID with semantic clustering by rrvgo.

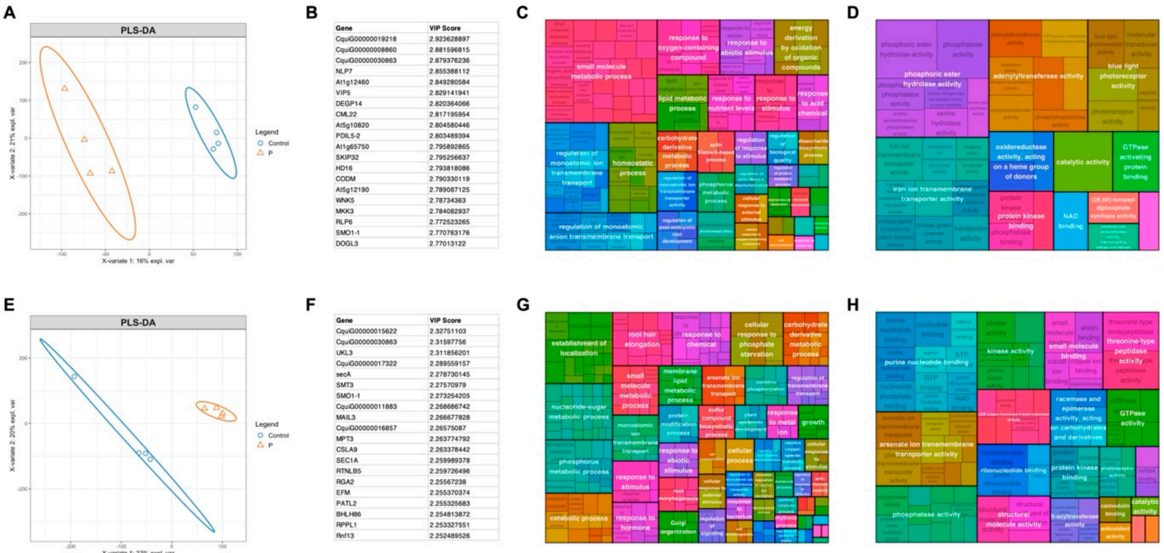

Supplemental Figure S14. Co-expression module characterization for module eigengenes strongly correlated with phosphorus deficiency in leaves and roots. (A) Treemap of enriched and semantically clustered biological process (BP) Gene Ontology terms for ME14 in leaves. (B) Treemap of enriched and semantically clustered molecular function (MF) Gene Ontology terms for ME14 in leaves. (C) Network plot of the top 20 hub genes in ME14 identified by intramodular connectivity, with node labels indicating gene names. (D) Treemap of enriched and semantically clustered BP Gene Ontology terms for ME27 in roots. (E) Treemap of enriched and semantically clustered MF Gene Ontology terms for ME27 in roots. (F) Network plot of the top 20 hub genes in ME27 identified by intramodular connectivity. GO term enrichment was performed using TRAPID and semantic clustering was performed using rrvgo. Module eigengenes were identified using the WGCNA package in R.

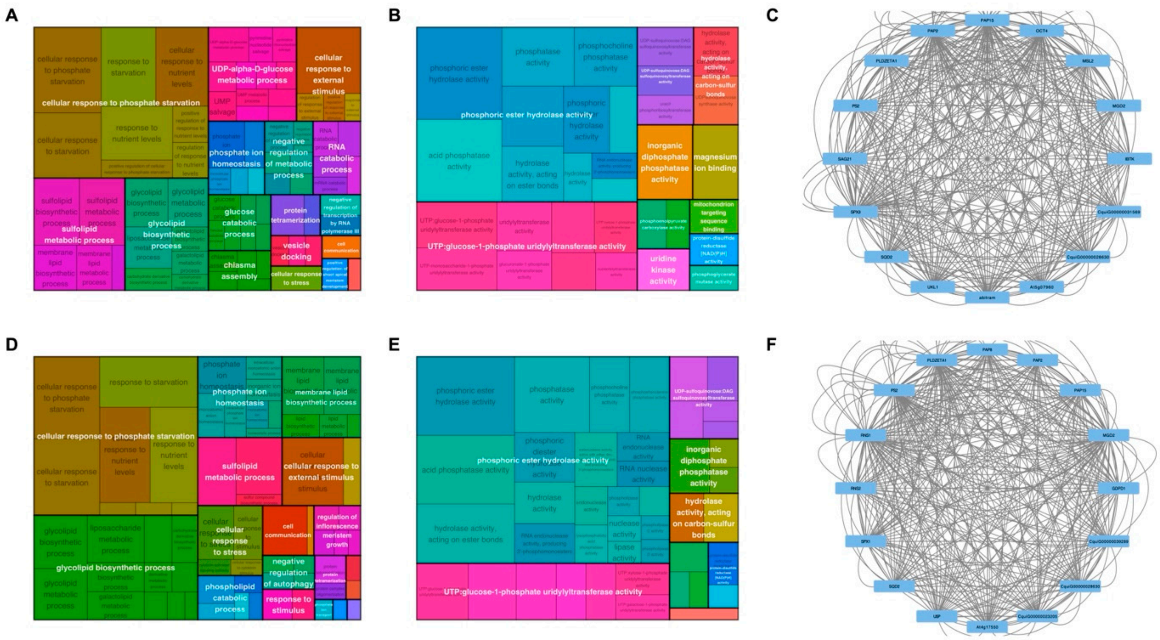

Supplemental Figure S15. Condition-specific differentially expressed genes (DEGs) under potassium deficiency in leaves and roots. (A) Stacked bar chart showing the total number of DEGs in each tissue, partitioned into unique (green) and shared (yellow) fractions. (B) Treemap of enriched and semantically clustered biological process (BP) Gene Ontology terms for potassium-specific leaf DEGs, with cluster size proportional to the number of genes associated with each term. (C) Treemap of enriched and semantically clustered molecular function (MF) Gene Ontology terms for potassium-specific leaf DEGs. (D) Table displaying enriched GO terms for potassium-specific root DEGs. GO term enrichment was performed using TRAPID and semantic clustering was performed using rrvgo.

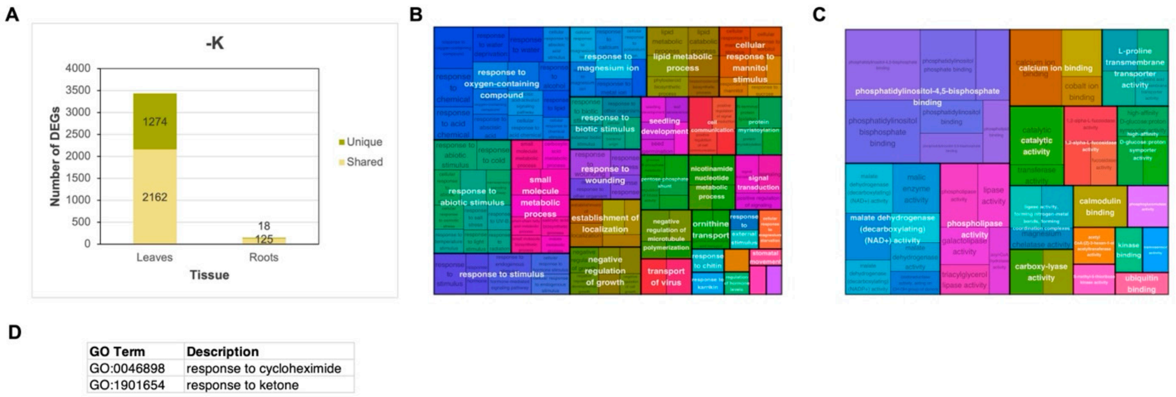

Supplemental Figure S16. Gene set enrichment analysis (GSEA) results for potassium-deficient leaf and root transcriptomes. (A) Treemap of enriched and semantically clustered biological

process (BP) Gene Ontology terms in leaves. (B) Treemap of enriched and semantically clustered molecular function (MF) Gene Ontology terms in leaves. (C) Dot plot of enriched KEGG pathways in leaves, faceted by direction of enrichment, with dot size proportional to the number of genes in each pathway and dot color indicating adjusted p-value. (D) Pathview rendering of the photosynthesis – antenna proteins KEGG pathway in leaves, with genes colored by $\log_2$FC on a blue to red scale, where blue indicates downregulation and red indicates upregulation. (E) Treemap of enriched and semantically clustered biological process (BP) Gene Ontology terms in roots. (F) Treemap of enriched and semantically clustered molecular function (MF) Gene Ontology terms in roots. (G) Dot plot of enriched KEGG pathways in roots, faceted by direction of enrichment, with dot size proportional to the number of genes in each pathway and dot color indicating adjusted p-value. (H) Pathview rendering of the betalain biosynthesis KEGG pathway in roots. GSEA was performed using the GSEA function in clusterProfiler with Benjamini-Hochberg correction and a p-value cutoff of 0.05. Pathway visualization was performed using Pathview.

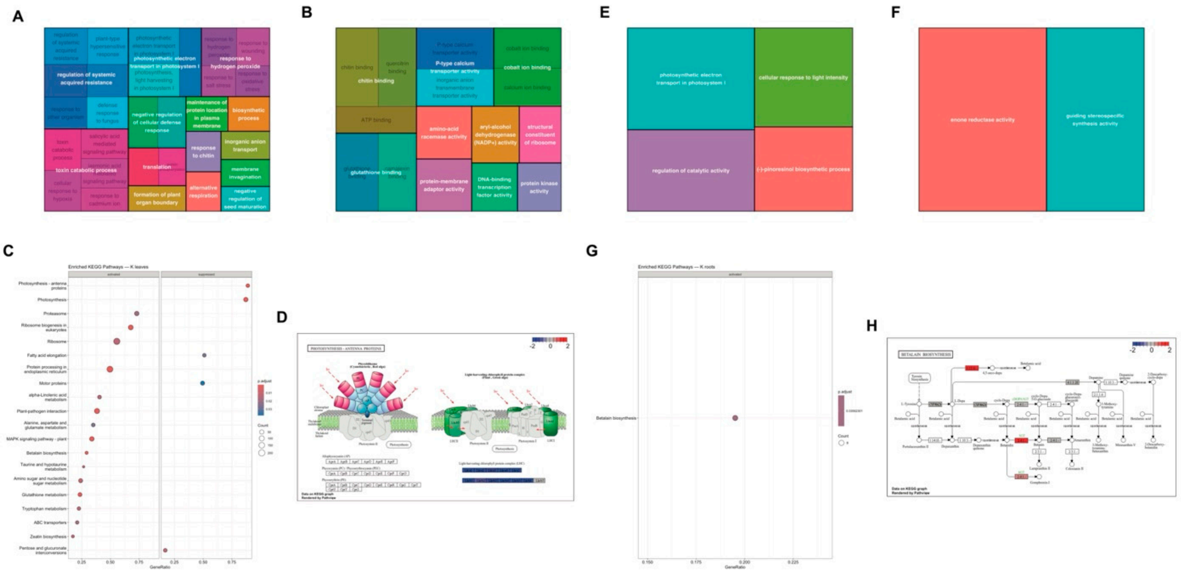

Supplemental Figure S17. Partial least squares discriminant analysis (PLS-DA) of potassium-deficient and control transcriptomes in leaves and roots. (A) Individuals plot for leaf samples, with each point representing a biological replicate colored by treatment group and 95% confidence ellipses shown for each group. The percentage of variance explained by each component is indicated on the respective axis. (B) Table of the top 20 genes ranked by variable importance in projection (VIP) score on component 1 for leaf samples, with gene name and VIP score shown. (C) Treemap of enriched and semantically clustered biological process (BP) Gene Ontology terms for leaf transcripts with a VIP score ≥ 1.5. (D) Treemap of enriched and semantically clustered molecular function (MF) Gene Ontology terms for leaf transcripts with a VIP score ≥ 1.5. (E) Individuals plot for root samples, with each point representing a biological

replicate colored by treatment group and 95% confidence ellipses shown for each group. (F) Table of the top 20 genes ranked by VIP score on component 1 for root samples. (G) Treemap of enriched and semantically clustered BP Gene Ontology terms for root transcripts with a VIP score ≥ 1.5. (H) Treemap of enriched and semantically clustered MF Gene Ontology terms for root transcripts with a VIP score ≥ 1.5. PLS-DA was performed using the mixOmics package in R and GO term enrichment was performed using TRAPID with semantic clustering by rrvgo.

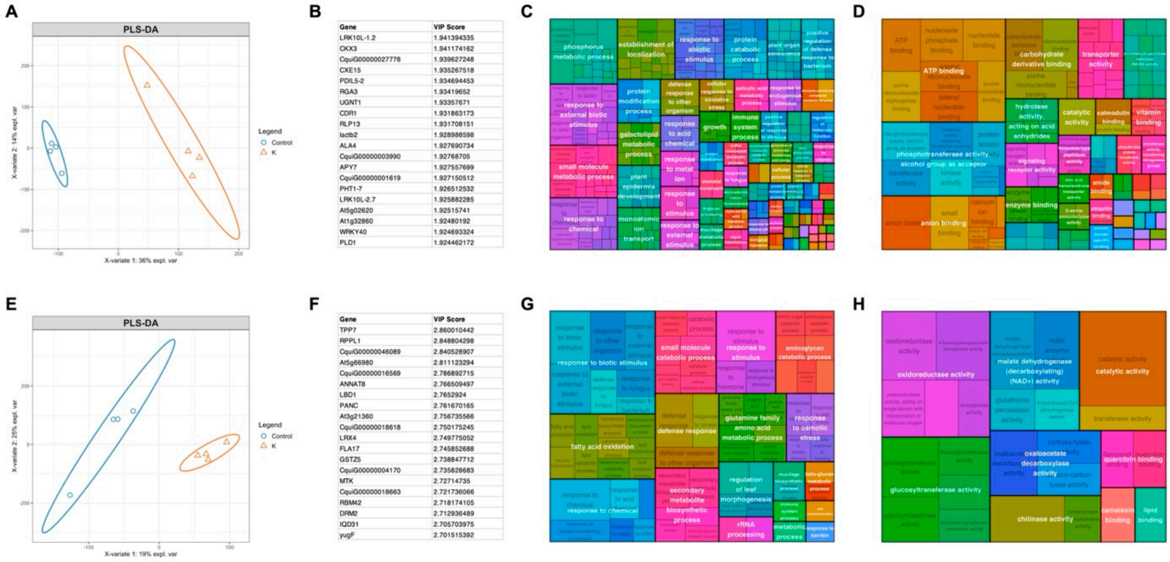

Supplemental Figure S18. Co-expression module characterization for module eigengenes strongly correlated with potassium deficiency in leaves. (A) Treemap of enriched and semantically clustered biological process (BP) Gene Ontology terms for ME4 in leaves. (B) Treemap of enriched and semantically clustered molecular function (MF) Gene Ontology terms for ME4 in leaves. (C) Network plot of the top 20 hub genes in ME4 identified by intramodular connectivity, with node labels indicating gene names. GO term enrichment was performed using TRAPID and semantic clustering was performed using rrvgo. Module eigengenes were identified using the WGCNA package in R.

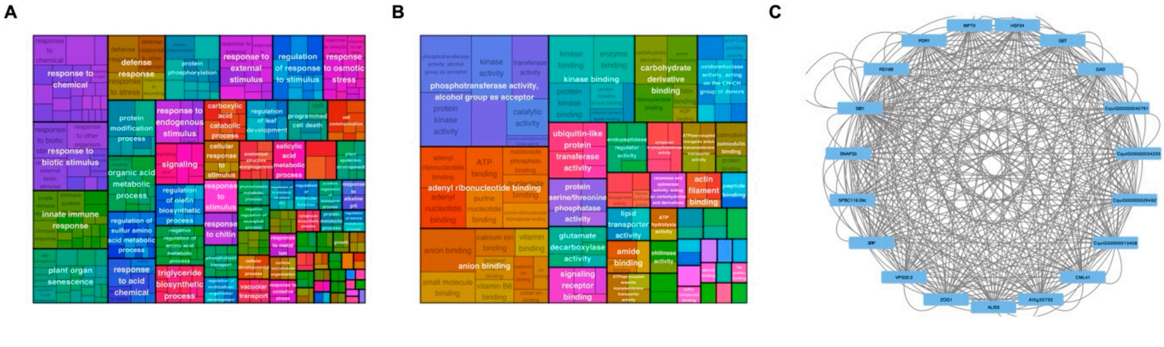

Supplemental Figure S19. Condition-specific differentially expressed genes (DEGs) under sulfur deficiency in leaves and roots. (A) Stacked bar chart showing the total number of DEGs in each tissue, partitioned into unique (green) and shared (yellow) fractions. (B) Treemap of enriched and semantically clustered biological process (BP) Gene Ontology terms for sulfur-specific leaf DEGs, with cluster size proportional to the number of genes associated with each term. (C) Treemap of enriched and semantically clustered molecular function (MF) Gene Ontology terms for sulfur-specific leaf DEGs. (D) Treemap of enriched and semantically clustered BP Gene Ontology terms for sulfur-specific root DEGs. (E) Treemap of enriched and semantically clustered MF Gene Ontology terms for sulfur-specific root DEGs. GO term enrichment was performed using TRAPID and semantic clustering was performed using rrvgo.

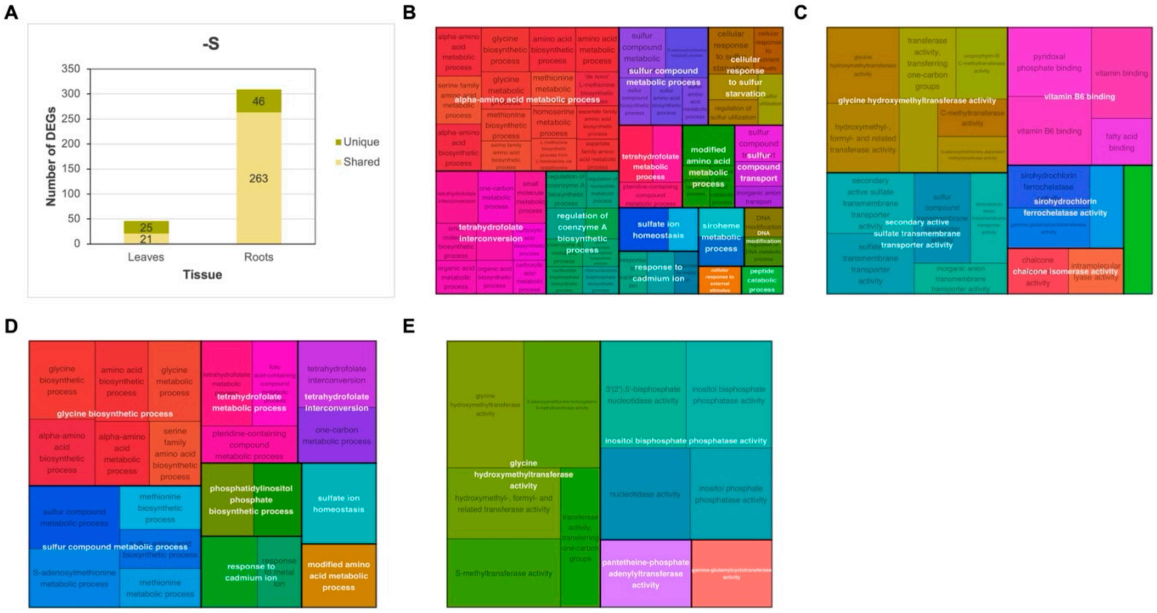

Supplemental Figure S20. Gene set enrichment analysis (GSEA) results for the sulfur-deficient leaf transcriptome. (A) Treemap of enriched and semantically clustered biological process (BP) Gene Ontology terms. (B) Treemap of enriched and semantically clustered molecular function (MF) Gene Ontology terms. GSEA was performed using the GSEA function in clusterProfiler with Benjamini-Hochberg correction and a p-value cutoff of 0.05.

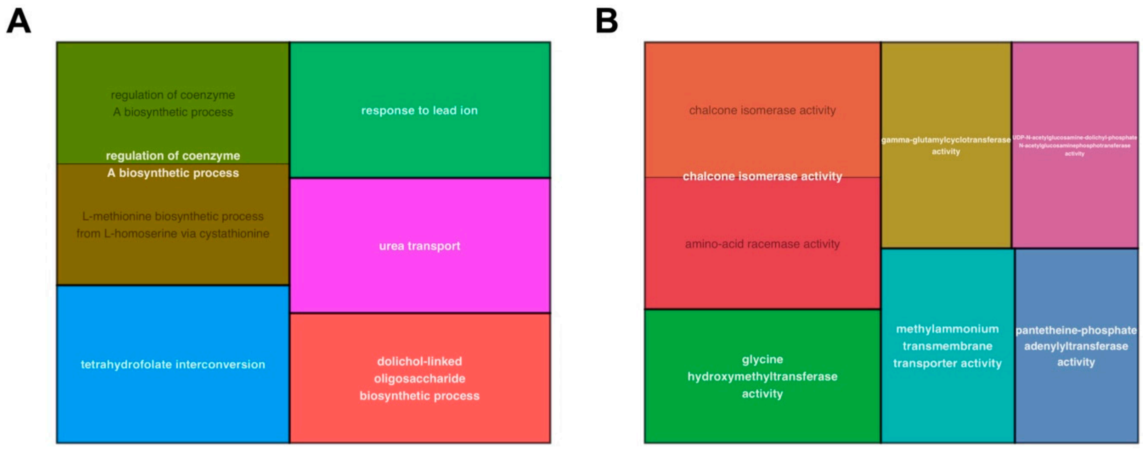

Supplemental Figure S21. Partial least squares discriminant analysis (PLS-DA) of sulfur-deficient and control transcriptomes in leaves and roots. (A) Individuals plot for leaf samples, with each point representing a biological replicate colored by treatment group and 95% confidence ellipses shown for each group. The percentage of variance explained by each component is indicated on the respective axis. (B) Table of the top 20 genes ranked by variable importance in projection (VIP) score on component 1 for leaf samples, with gene name and VIP score shown. (C) Treemap of enriched and semantically clustered biological process (BP) Gene Ontology terms for leaf transcripts with a VIP score ≥ 1.5. (D) Treemap of enriched and semantically clustered molecular function (MF) Gene Ontology terms for leaf transcripts with a VIP score ≥ 1.5. (E) Individuals plot for root samples, with each point representing a biological replicate colored by treatment group and 95% confidence ellipses shown for each group. (F) Table of the top 20 genes ranked by VIP score on component 1 for root samples. (G) Treemap of enriched and semantically clustered BP Gene Ontology terms for root transcripts with a VIP score ≥ 1.5. (H) Treemap of enriched and semantically clustered MF Gene Ontology terms for root transcripts with a VIP score ≥ 1.5. PLS-DA was performed using the mixOmics package in R and GO term enrichment was performed using TRAPID with semantic clustering by rrvgo.

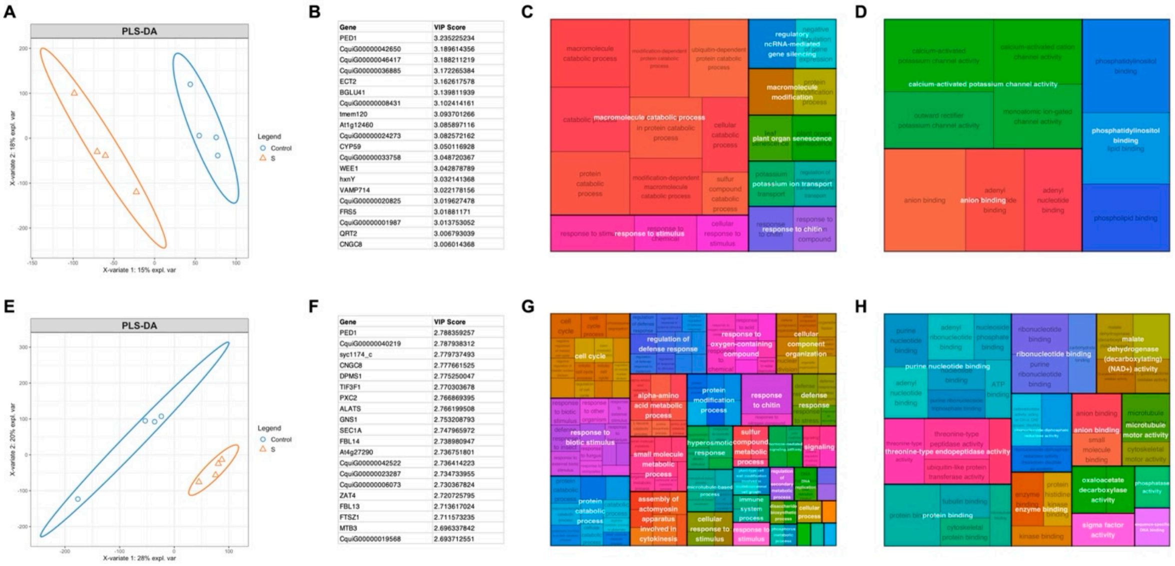

Supplemental Figure S22. Co-expression module characterization for module eigengenes strongly correlated with sulfur deficiency in leaves and roots. (A) Treemap of enriched and semantically clustered biological process (BP) Gene Ontology terms for ME31 in leaves. (B) Treemap of enriched and semantically clustered molecular function (MF) Gene Ontology terms for ME31 in leaves. (C) Network plot of the top 20 hub genes in ME31 identified by intramodular connectivity, with node labels indicating gene names. (D) Treemap of enriched and semantically clustered BP Gene Ontology terms for ME41 in roots. (E) Treemap of enriched and semantically clustered MF Gene Ontology terms for ME41 in roots. (F) Network plot of the top 20 hub genes in ME41 identified by intramodular connectivity. GO term enrichment was performed using TRAPID and semantic clustering was performed using rrvgo. Module eigengenes were identified using the WGCNA package in R.

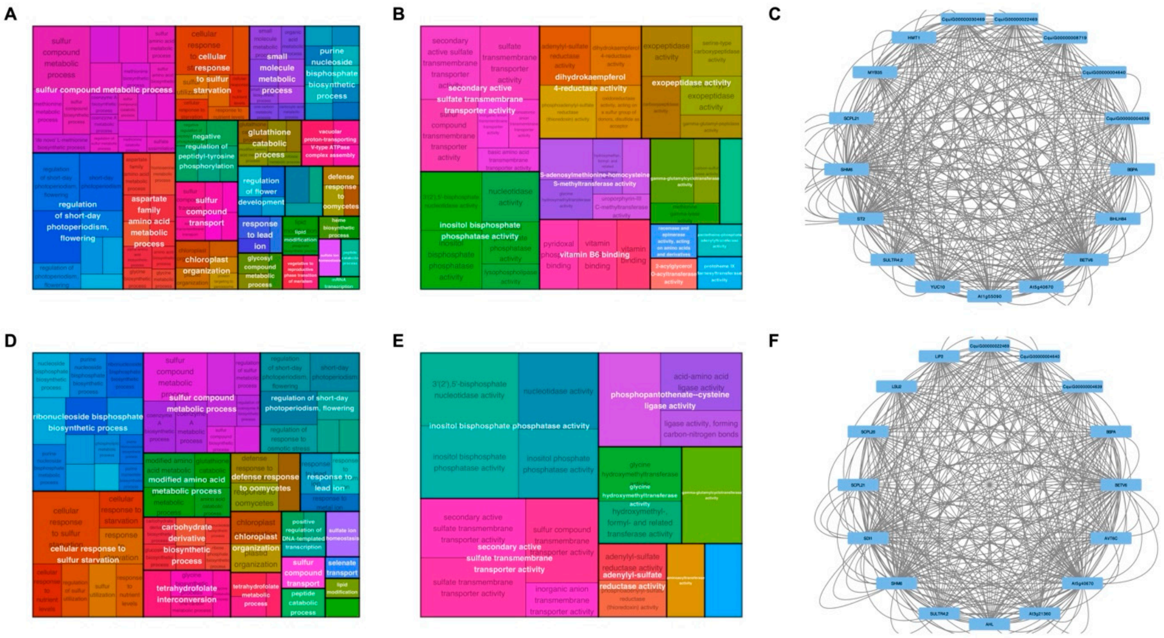

Supplemental Figure S23. Condition-specific differentially expressed genes (DEGs) under calcium deficiency in leaves and roots displayed as a stacked bar chart. The total number of DEGs in each tissue are partitioned into unique (green) and shared (yellow) fractions.

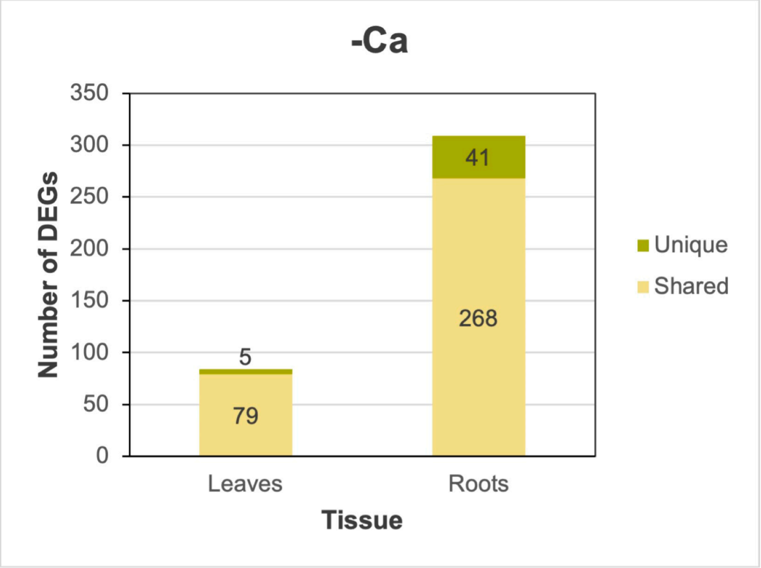

Supplemental Figure S24. Gene set enrichment analysis (GSEA) results for the calcium-deficient leaf transcriptome. (A) Treemap of enriched and semantically clustered biological process (BP) Gene Ontology terms. (B) Treemap of enriched and semantically clustered molecular function (MF) Gene Ontology terms. (C) Dot plot of enriched KEGG pathways, faceted by direction of enrichment, with dot size proportional to the number of genes in each

pathway and dot color indicating adjusted p-value. (D) Pathview rendering of the phenylpropanoid biosynthesis KEGG pathway, with genes colored by log_2FC on a blue to red scale, where blue indicates downregulation and red indicates upregulation. GSEA was performed using the GSEA function in clusterProfiler with Benjamini-Hochberg correction and a p-value cutoff of 0.05. Pathway visualization was performed using Pathview.

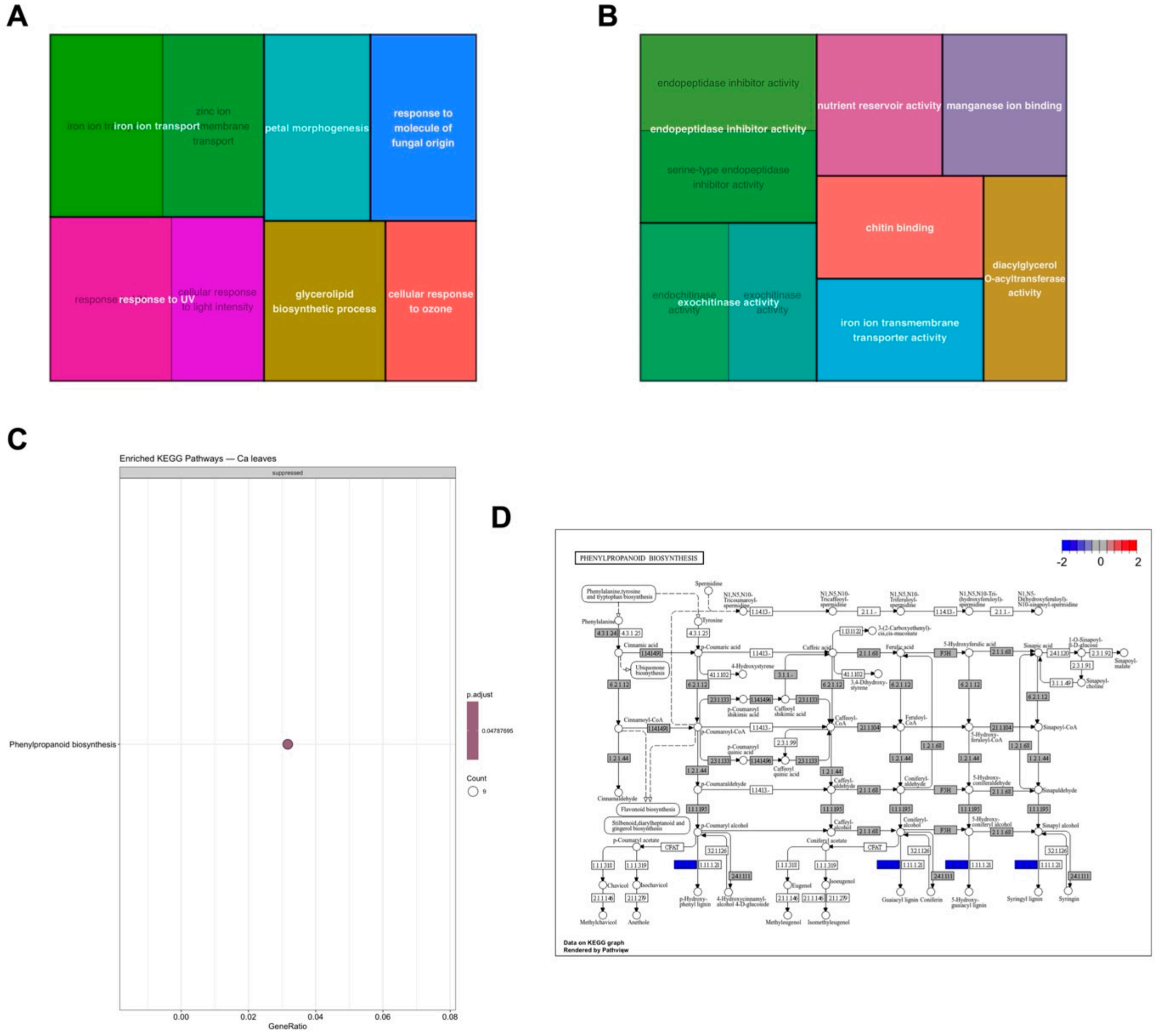

Supplemental Figure S25. Partial least squares discriminant analysis (PLS-DA) of calcium-deficient and control transcriptomes in leaves and roots. (A) Individuals plot for leaf samples, with each point representing a biological replicate colored by treatment group and 95% confidence ellipses shown for each group. The percentage of variance explained by each component is indicated on the respective axis. (B) Table of the top 20 genes ranked by variable importance in projection (VIP) score on component 1 for leaf samples, with gene name and VIP score shown. (C) Treemap of enriched and semantically clustered biological process (BP) Gene

Ontology terms for leaf transcripts with a VIP score ≥ 1.5. (D) Treemap of enriched and semantically clustered molecular function (MF) Gene Ontology terms for leaf transcripts with a VIP score ≥ 1.5. (E) Individuals plot for root samples, with each point representing a biological replicate colored by treatment group and 95% confidence ellipses shown for each group. (F) Table of the top 20 genes ranked by VIP score on component 1 for root samples. (G) Treemap of enriched and semantically clustered BP Gene Ontology terms for root transcripts with a VIP score ≥ 1.5. (H) Treemap of enriched and semantically clustered MF Gene Ontology terms for root transcripts with a VIP score ≥ 1.5. PLS-DA was performed using the mixOmics package in R and GO term enrichment was performed using TRAPID with semantic clustering by rrvgo.

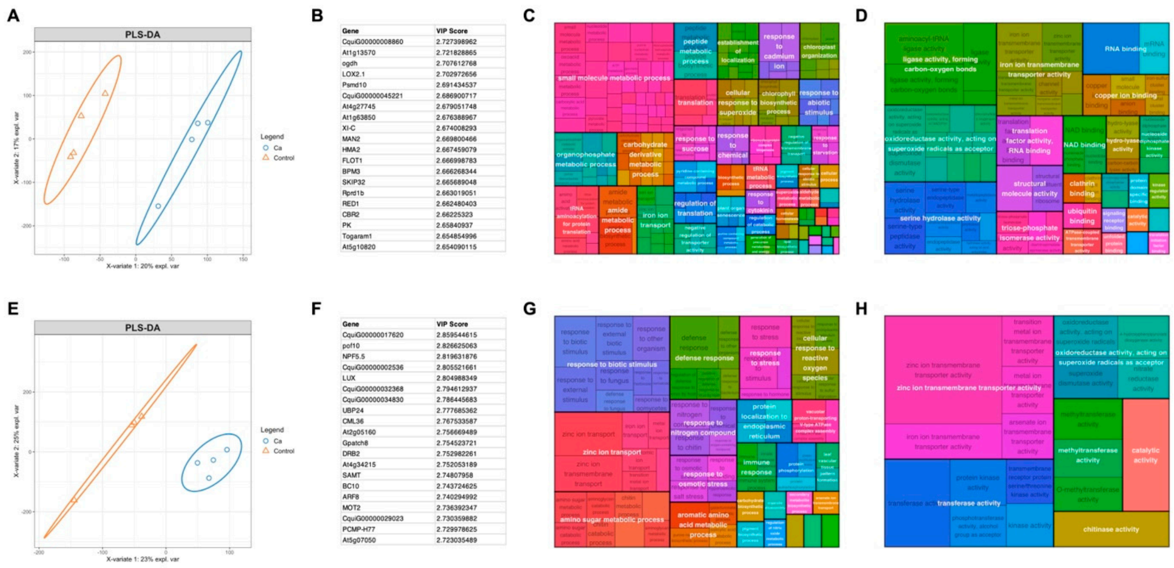

Supplemental Figure S26. Condition-specific differentially expressed genes (DEGs) under magnesium deficiency in leaves and roots. (A) Stacked bar chart showing the total number of DEGs in each tissue, partitioned into unique (green) and shared (yellow) fractions. (B) Treemap of enriched and semantically clustered biological process (BP) Gene Ontology terms for magnesium-specific leaf DEGs, with cluster size proportional to the number of genes associated with each term. (C) Treemap of enriched and semantically clustered molecular function (MF) Gene Ontology terms for magnesium-specific leaf DEGs. (D) Table displaying enriched GO terms for magnesium-specific root DEGs. GO term enrichment was performed using TRAPID and semantic clustering was performed using rrvgo.

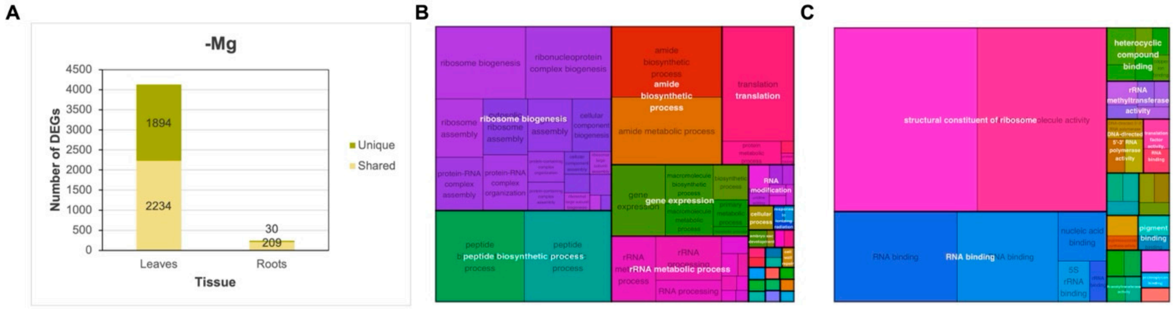

D

GO Term	Description
GO:0010076	maintenance of floral meristem identity
GO:0010077	maintenance of inflorescence meristem identity

Supplemental Figure S27. Gene set enrichment analysis (GSEA) results for the magnesium-deficient leaf transcriptome. (A) Treemap of enriched and semantically clustered biological process (BP) Gene Ontology terms. (B) Treemap of enriched and semantically clustered molecular function (MF) Gene Ontology terms. (C) Dot plot of enriched KEGG pathways, faceted by direction of enrichment, with dot size proportional to the number of genes in each pathway and dot color indicating adjusted p-value. (D) Pathview rendering of the photosynthesis – antenna proteins KEGG pathway, with genes colored by $\log_2$FC on a blue to red scale, where blue indicates downregulation and red indicates upregulation. GSEA was performed using the GSEA function in clusterProfiler with Benjamini-Hochberg correction and a p-value cutoff of 0.05. Pathway visualization was performed using Pathview.

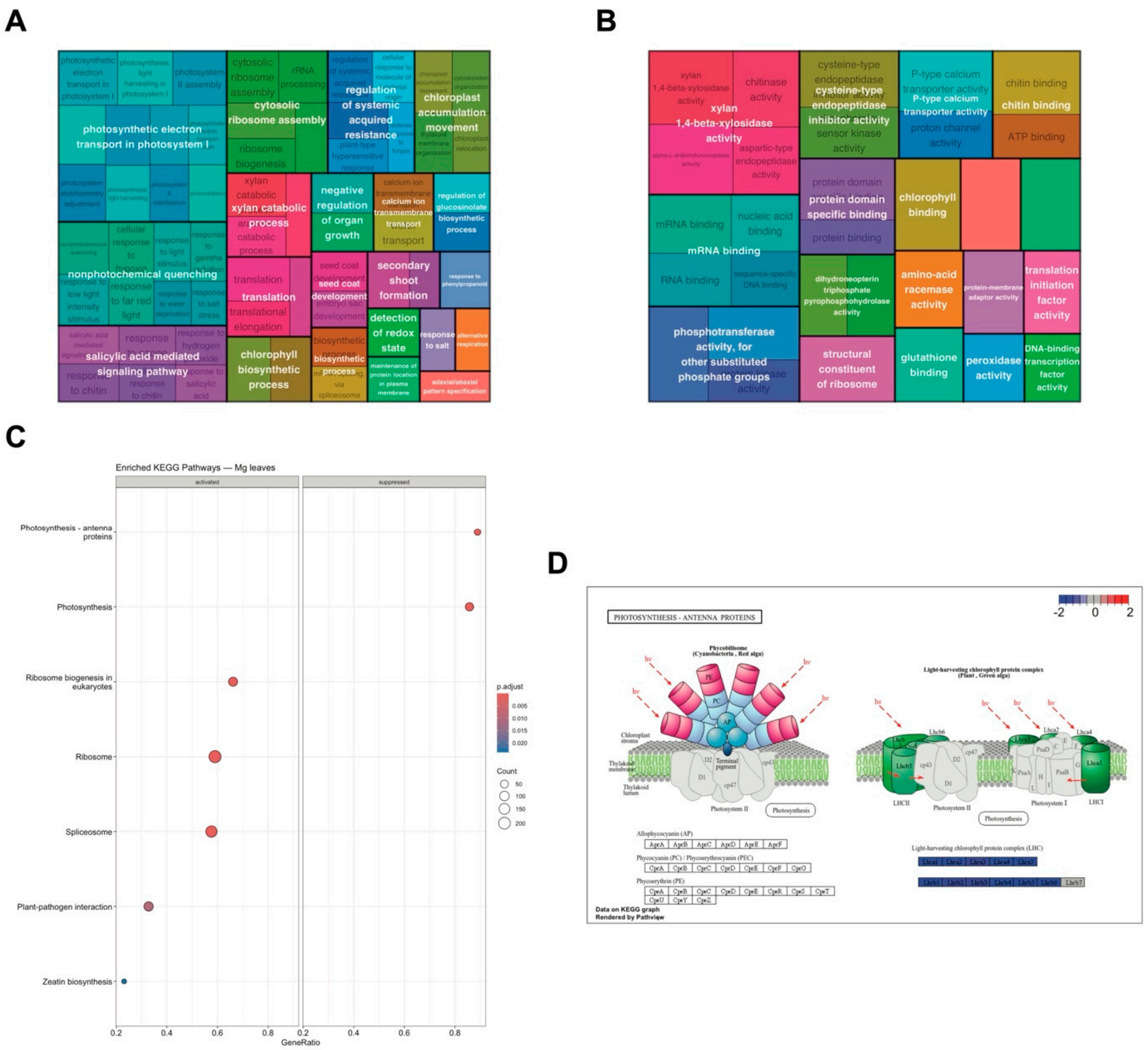

Supplemental Figure S28. Partial least squares discriminant analysis (PLS-DA) of magnesium-deficient and control transcriptomes in leaves and roots. (A) Individuals plot for leaf samples, with each point representing a biological replicate colored by treatment group and 95% confidence ellipses shown for each group. The percentage of variance explained by each component is indicated on the respective axis. (B) Table of the top 20 genes ranked by variable importance in projection (VIP) score on component 1 for leaf samples, with gene name and VIP score shown. (C) Treemap of enriched and semantically clustered biological process (BP) Gene Ontology terms for leaf transcripts with a VIP score ≥ 1.5. (D) Treemap of enriched and semantically clustered molecular function (MF) Gene Ontology terms for leaf transcripts with a VIP score ≥ 1.5. (E) Individuals plot for root samples, with each point representing a biological replicate colored by treatment group and 95% confidence ellipses shown for each group. (F) Table of the top 20 genes ranked by VIP score on component 1 for root samples. (G) Treemap of enriched and semantically clustered BP Gene Ontology terms for root transcripts with a VIP

score ≥ 1.5. (H) Treemap of enriched and semantically clustered MF Gene Ontology terms for root transcripts with a VIP score ≥ 1.5. PLS-DA was performed using the mixOmics package in R and GO term enrichment was performed using TRAPID with semantic clustering by rrvgo.

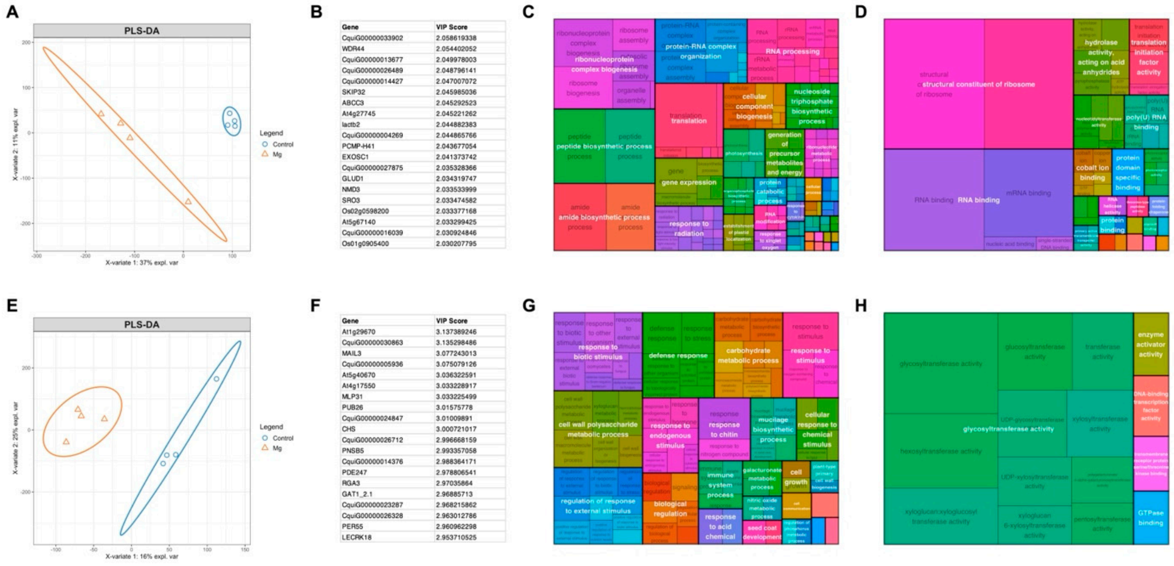

Supplemental Figure S29. Co-expression module characterization for module eigengenes strongly correlated with magnesium deficiency in leaves and roots. (A) Treemap of enriched and semantically clustered biological process (BP) Gene Ontology terms for ME30 in leaves. (B) Treemap of enriched and semantically clustered molecular function (MF) Gene Ontology terms for ME30 in leaves. (C) Network plot of the top 20 hub genes in ME30 identified by intramodular connectivity, with node labels indicating gene names. (D) Treemap of enriched and semantically clustered BP Gene Ontology terms for ME46 in roots. (E) Treemap of enriched and semantically clustered MF Gene Ontology terms for ME46 in roots. (F) Network plot of the top 20 hub genes in ME46 identified by intramodular connectivity. GO term enrichment was performed using TRAPID and semantic clustering was performed using rrvgo. Module eigengenes were identified using the WGCNA package in R.

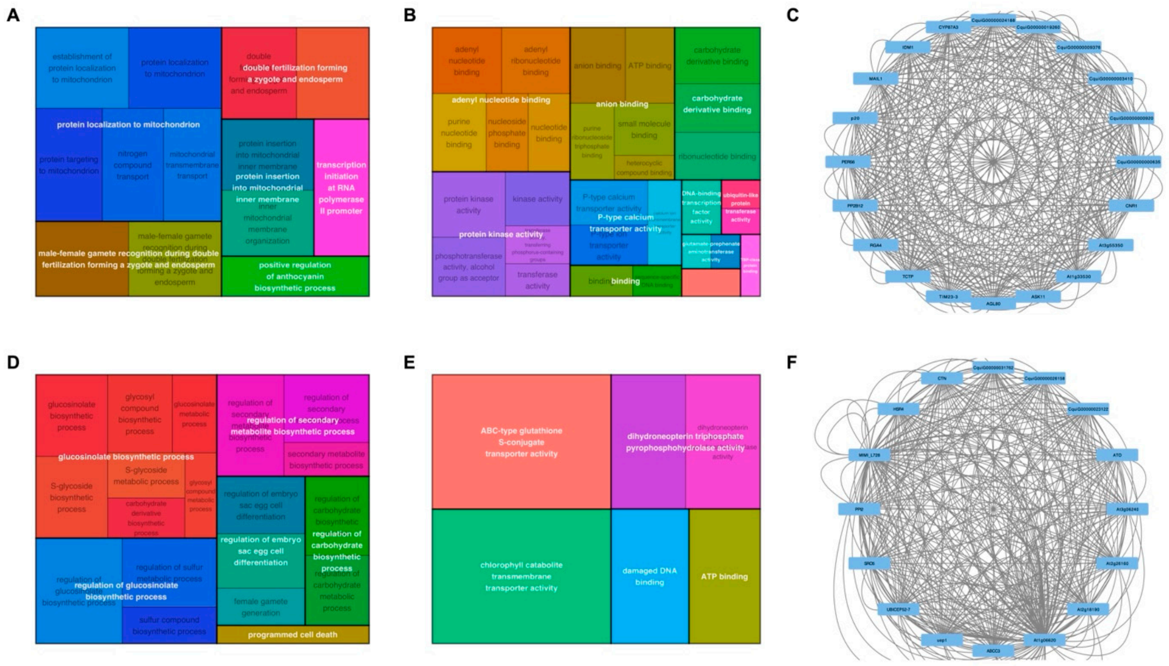

Supplemental Figure S30. Condition-specific differentially expressed genes (DEGs) under zinc deficiency in leaves and roots. (A) Stacked bar chart showing the total number of DEGs in each tissue, partitioned into unique (green) and shared (yellow) fractions. (B) Treemap of enriched and semantically clustered biological process (BP) Gene Ontology terms for zinc-specific leaf DEGs, with cluster size proportional to the number of genes associated with each term. (C) Treemap of enriched and semantically clustered molecular function (MF) Gene Ontology terms for zinc-specific leaf DEGs. (D) Treemap of enriched and semantically clustered BP Gene Ontology terms for zinc-specific root DEGs. (E) Treemap of enriched and semantically clustered MF Gene Ontology terms for zinc-specific root DEGs. GO term enrichment was performed using TRAPID and semantic clustering was performed using rrvgo.

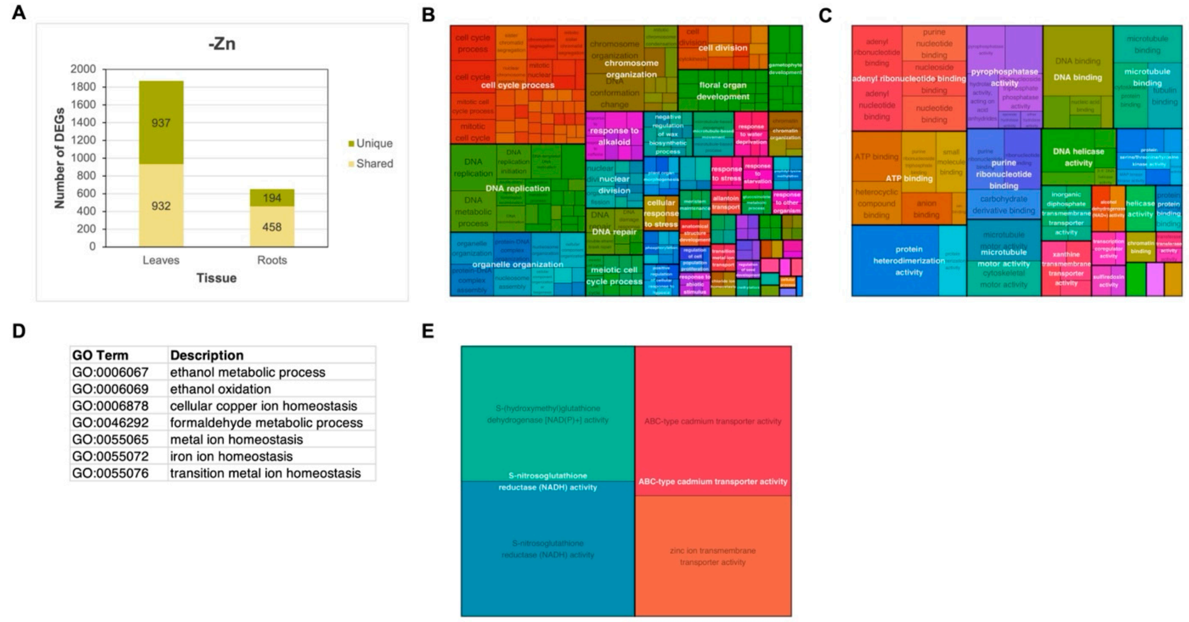

Supplemental Figure S31. Gene set enrichment analysis (GSEA) results for zinc-deficient leaf and root transcriptomes. (A) Treemap of enriched and semantically clustered biological process (BP) Gene Ontology terms in leaves. (B) Treemap of enriched and semantically clustered molecular function (MF) Gene Ontology terms in leaves. (C) Dot plot of enriched KEGG pathways in leaves, faceted by direction of enrichment, with dot size proportional to the number of genes in each pathway and dot color indicating adjusted p-value. (D) Pathview rendering of the mismatch repair metabolism KEGG pathway in leaves, with genes colored by log_2FC on a blue to red scale, where blue indicates downregulation and red indicates upregulation. (E) Treemap of enriched and semantically clustered biological process (BP) Gene Ontology terms in roots. (F) Treemap of enriched and semantically clustered molecular function (MF) Gene Ontology terms in roots. (G) Dot plot of enriched KEGG pathways in roots, faceted by direction of enrichment, with dot size proportional to the number of genes in each pathway and dot color indicating adjusted p-value. (H) Pathview rendering of the DNA replication KEGG pathway in roots. GSEA was performed using the GSEA function in clusterProfiler with Benjamini-Hochberg correction and a p-value cutoff of 0.05. Pathway visualization was performed using Pathview.

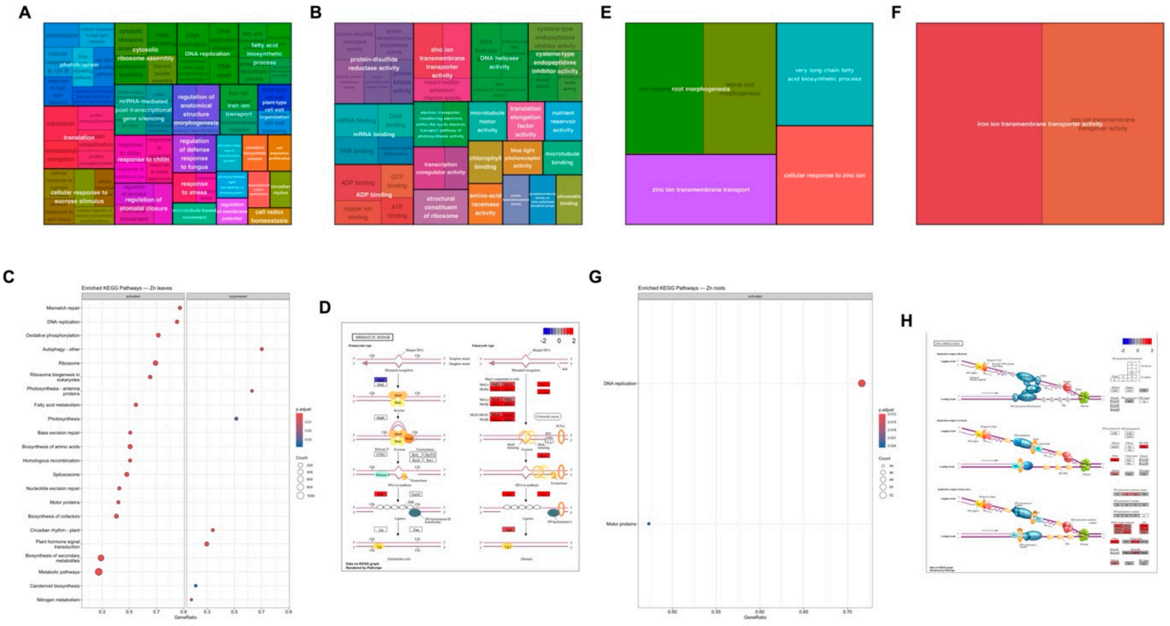

Supplemental Figure S32. Partial least squares discriminant analysis (PLS-DA) of zinc-deficient and control transcriptomes in leaves and roots. (A) Individuals plot for leaf samples, with each point representing a biological replicate colored by treatment group and 95% confidence ellipses shown for each group. The percentage of variance explained by each component is indicated on the respective axis. (B) Table of the top 20 genes ranked by variable importance in projection (VIP) score on component 1 for leaf samples, with gene name and VIP score shown. (C) Treemap of enriched and semantically clustered biological process (BP) Gene Ontology terms for leaf transcripts with a VIP score ≥ 1.5. (D) Treemap of enriched and semantically clustered molecular function (MF) Gene Ontology terms for leaf transcripts with a VIP score ≥ 1.5. (E) Individuals plot for root samples, with each point representing a biological replicate colored by treatment group and 95% confidence ellipses shown for each group. (F) Table of the top 20 genes ranked by VIP score on component 1 for root samples. (G) Treemap of enriched and semantically clustered BP Gene Ontology terms for root transcripts with a VIP score ≥ 1.5. (H) Treemap of enriched and semantically clustered MF Gene Ontology terms for root transcripts with a VIP score ≥ 1.5. PLS-DA was performed using the mixOmics package in R and GO term enrichment was performed using TRAPID with semantic clustering by rrvgo.

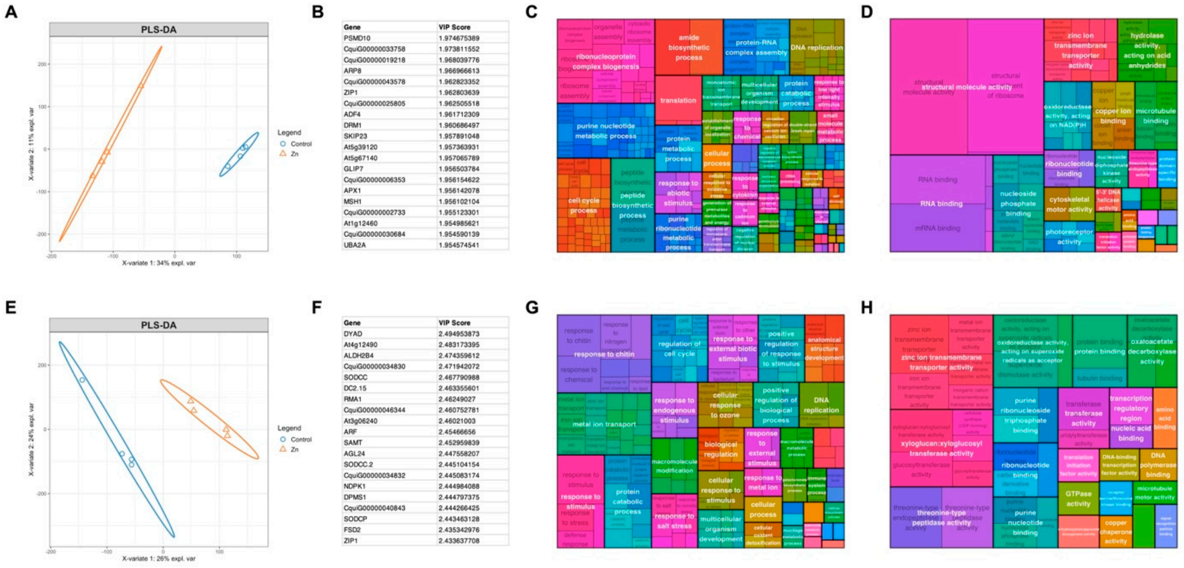

Gene	VIP Score
PSMD10	1.974675389
CquiG00000033758	1.973811552
CquiG00000019218	1.968039776
ARP8	1.966966613
CquiG00000043578	1.962823352
ZIP1	1.962803639
CquiG00000025805	1.962505518
ADF4	1.961712309
DRM1	1.960686497
SKIP23	1.957891048
At5g39120	1.957363931
At5g67140	1.957065789
GLIP7	1.956503784
CquiG00000006353	1.956154622
APX1	1.956142078
MSH1	1.956102104
CquiG00000002733	1.955123301
At1g12460	1.954985621
CquiG00000030684	1.954590139
UBA2A	1.954574541

Gene	VIP Score
DYAD	2.494953873
At4g12490	2.483173395
ALDH2B4	2.474359612
CquiG00000034830	2.471942072
SODCC	2.467790988
DC2.15	2.463355601
RMA1	2.46249027
CquiG00000046344	2.460752781
At3g06240	2.46021003
ARF	2.45466656
SAMT	2.452959839
AGL24	2.447558207
SODCC.2	2.445104154
CquiG00000034832	2.445083174
NDPK1	2.444984088
DPMS1	2.444797375
CquiG00000040843	2.444266425
SODCP	2.443463128
FSD2	2.435342976
ZIP1	2.433637708

Supplemental Figure S33. Co-expression module characterization for module eigengenes strongly correlated with zinc deficiency in leaves and roots. (A) Treemap of enriched and semantically clustered biological process (BP) Gene Ontology terms for ME11 in leaves. (B) Treemap of enriched and semantically clustered molecular function (MF) Gene Ontology terms for ME11 in leaves. (C) Network plot of the top 20 hub genes in ME11 identified by intramodular connectivity, with node labels indicating gene names. (D) Treemap of enriched and semantically clustered BP Gene Ontology terms for ME26 in roots. (E) Treemap of enriched and semantically clustered MF Gene Ontology terms for ME26 in roots. (F) Network plot of the top 20 hub genes in ME26 identified by intramodular connectivity. GO term enrichment was performed using TRAPID and semantic clustering was performed using rrvgo. Module eigengenes were identified using the WGCNA package in R.

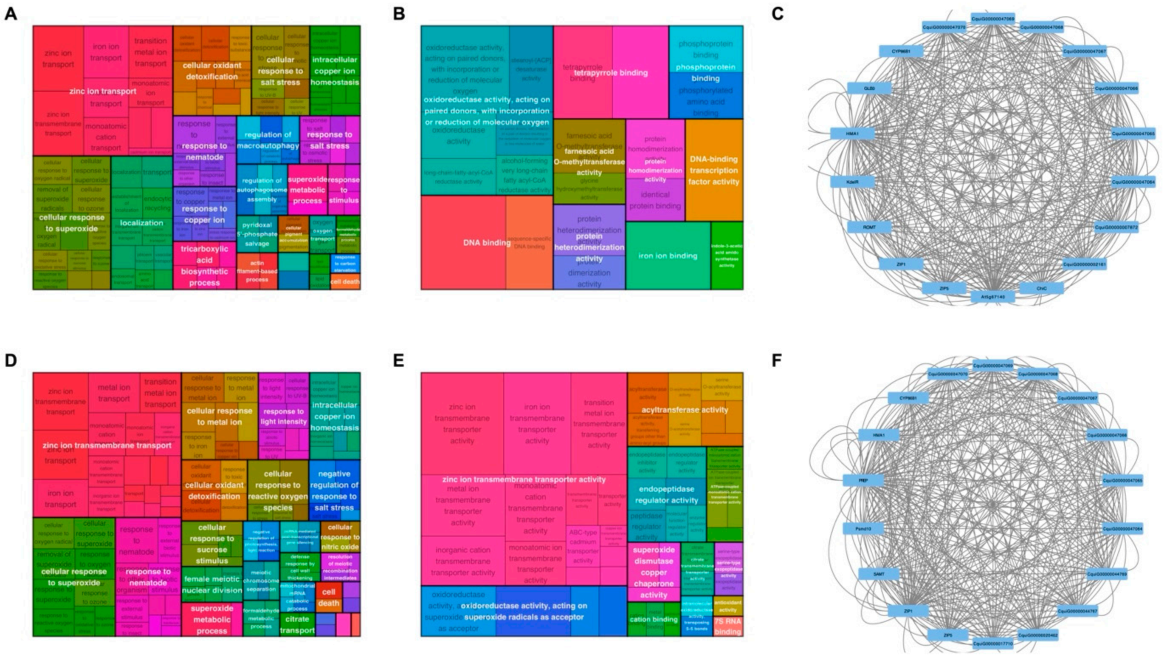

Supplemental Figure S34. Condition-specific differentially expressed genes (DEGs) under iron deficiency in leaves and roots. (A) Stacked bar chart showing the total number of DEGs in each tissue, partitioned into unique (green) and shared (yellow) fractions. (B) Treemap of enriched and semantically clustered biological process (BP) Gene Ontology terms for iron-specific leaf DEGs, with cluster size proportional to the number of genes associated with each term. (C) Treemap of enriched and semantically clustered molecular function (MF) Gene Ontology terms for iron-specific leaf DEGs. (D) Treemap of enriched and semantically clustered BP Gene Ontology terms for iron-specific root DEGs. (E) Treemap of enriched and semantically clustered MF Gene Ontology terms for iron-specific root DEGs. GO term enrichment was performed using TRAPID and semantic clustering was performed using rrvgo.

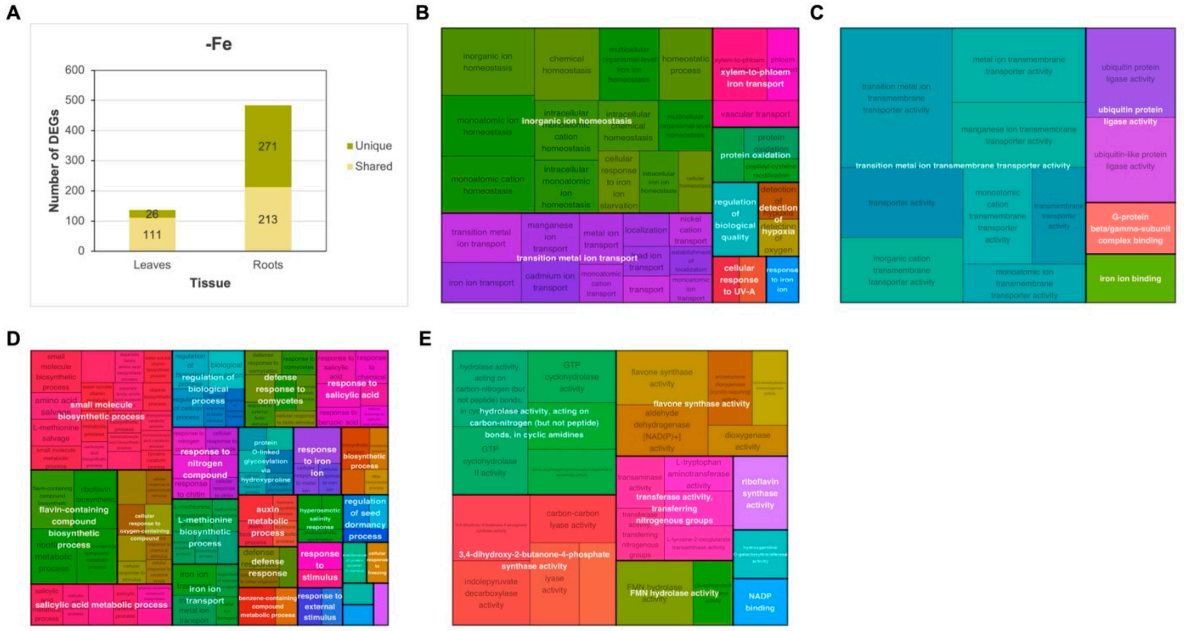

Supplemental Figure S35. Gene set enrichment analysis (GSEA) results for iron-deficient leaf and root transcriptomes. (A) Treemap of enriched and semantically clustered biological process (BP) Gene Ontology terms in leaves. (B) Treemap of enriched and semantically clustered molecular function (MF) Gene Ontology terms in leaves. (C) Dot plot of enriched KEGG pathways in leaves, faceted by direction of enrichment, with dot size proportional to the number of genes in each pathway and dot color indicating adjusted p-value. (D) Pathview rendering of the mismatch repair metabolism KEGG pathway in leaves, with genes colored by log_2FC on a blue to red scale, where blue indicates downregulation and red indicates upregulation. (E) Treemap of enriched and semantically clustered biological process (BP) Gene Ontology terms in roots. (F) Treemap of enriched and semantically clustered molecular function (MF) Gene Ontology terms in roots. (G) Dot plot of enriched KEGG pathways in roots, faceted by direction of enrichment, with dot size proportional to the number of genes in each pathway and dot color indicating adjusted p-value. (H) Pathview rendering of the riboflavin metabolism KEGG pathway in roots. GSEA was performed using the GSEA function in clusterProfiler with Benjamini-Hochberg correction and a p-value cutoff of 0.05. Pathway visualization was performed using Pathview.

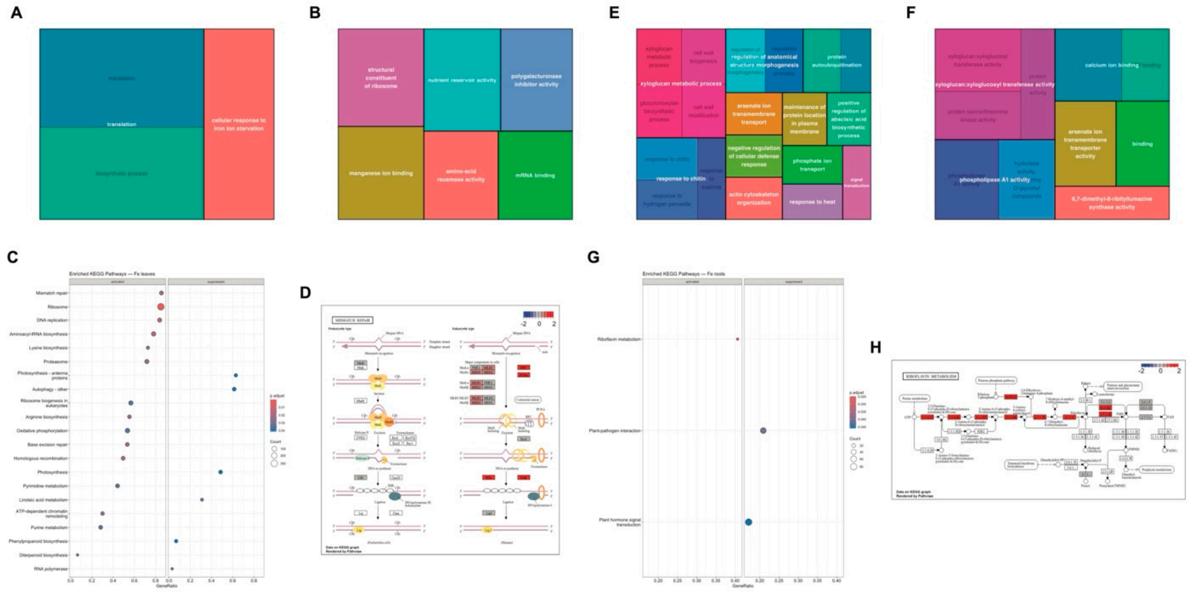

Supplemental Figure S36. Partial least squares discriminant analysis (PLS-DA) of iron-deficient and control transcriptomes in leaves and roots. (A) Individuals plot for leaf samples, with each point representing a biological replicate colored by treatment group and 95% confidence ellipses shown for each group. The percentage of variance explained by each component is indicated on the respective axis. (B) Table of the top 20 genes ranked by variable importance in projection (VIP) score on component 1 for leaf samples, with gene name and VIP score shown. (C) Treemap of enriched and semantically clustered biological process (BP) Gene Ontology terms for leaf transcripts with a VIP score ≥ 1.5. (D) Treemap of enriched and semantically clustered molecular function (MF) Gene Ontology terms for leaf transcripts with a VIP score ≥ 1.5. (E) Individuals plot for root samples, with each point representing a biological replicate colored by treatment group and 95% confidence ellipses shown for each group. (F) Table of the top 20 genes ranked by VIP score on component 1 for root samples. (G) Treemap of enriched and semantically clustered BP Gene Ontology terms for root transcripts with a VIP score ≥ 1.5. (H) Treemap of enriched and semantically clustered MF Gene Ontology terms for root transcripts with a VIP score ≥ 1.5. PLS-DA was performed using the mixOmics package in R and GO term enrichment was performed using TRAPID with semantic clustering by rrvgo.

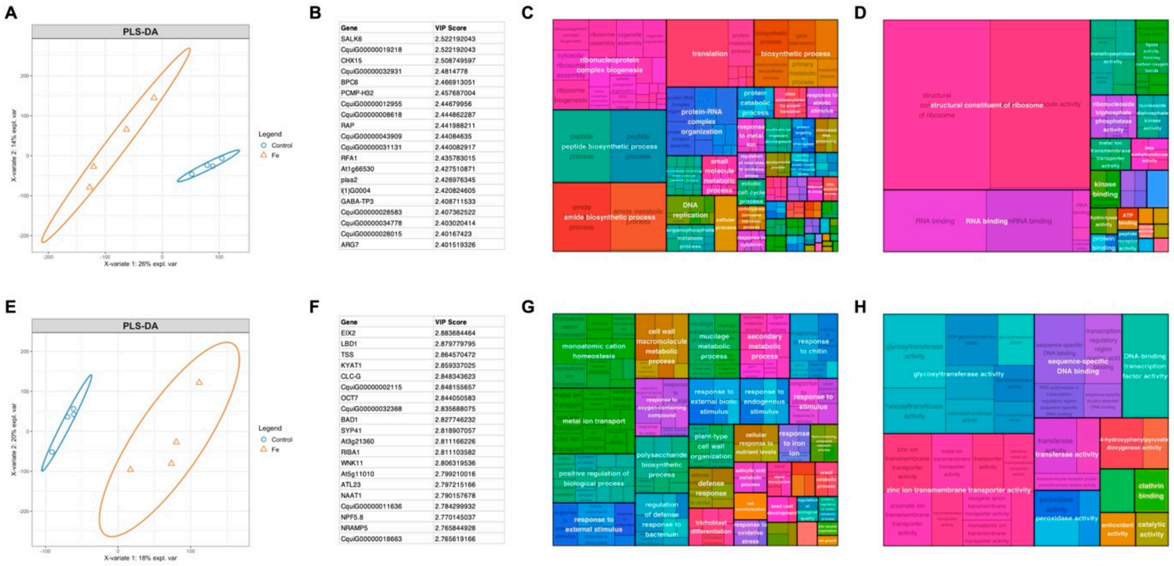

Supplemental Figure S37. Co-expression module characterization for module eigengenes strongly correlated with iron deficiency in leaves and roots. (A) Treemap of enriched and semantically clustered biological process (BP) Gene Ontology terms for ME40 in leaves. (B) Treemap of enriched and semantically clustered molecular function (MF) Gene Ontology terms for ME40 in leaves. (C) Network plot of the top 20 hub genes in ME40 identified by intramodular connectivity, with node labels indicating gene names. (D) Treemap of enriched and semantically clustered BP Gene Ontology terms for ME15 in roots. (E) Treemap of enriched and semantically clustered MF Gene Ontology terms for ME15 in roots. (F) Network plot of the top 20 hub genes in ME15 identified by intramodular connectivity. GO term enrichment was performed using TRAPID and semantic clustering was performed using rrvgo. Module eigengenes were identified using the WGCNA package in R.

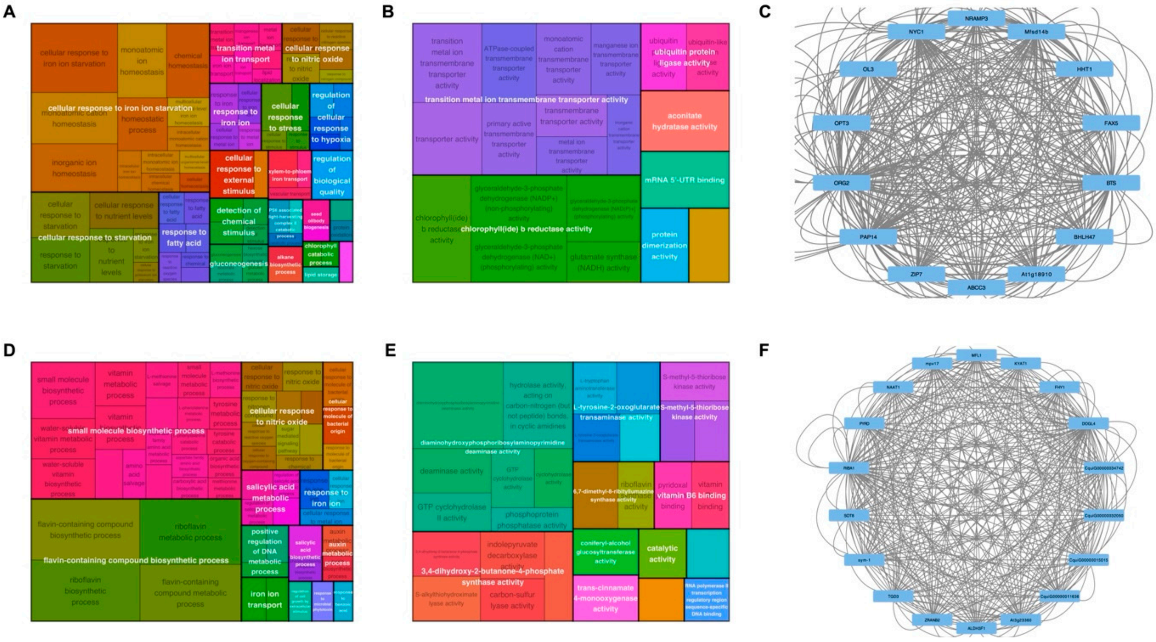

Supplemental Figure S38. Condition-specific differentially expressed genes (DEGs) under manganese deficiency in leaves and roots. (A) Stacked bar chart showing the total number of DEGs in each tissue, partitioned into unique (green) and shared (yellow) fractions. (B) Treemap of enriched and semantically clustered biological process (BP) Gene Ontology terms for manganese-specific leaf DEGs, with cluster size proportional to the number of genes associated with each term. (C) Treemap of enriched and semantically clustered molecular function (MF) Gene Ontology terms for manganese-specific leaf DEGs. (D) Treemap of enriched and semantically clustered BP Gene Ontology terms for manganese-specific root DEGs. GO term enrichment was performed using TRAPID and semantic clustering was performed using rrvgo.

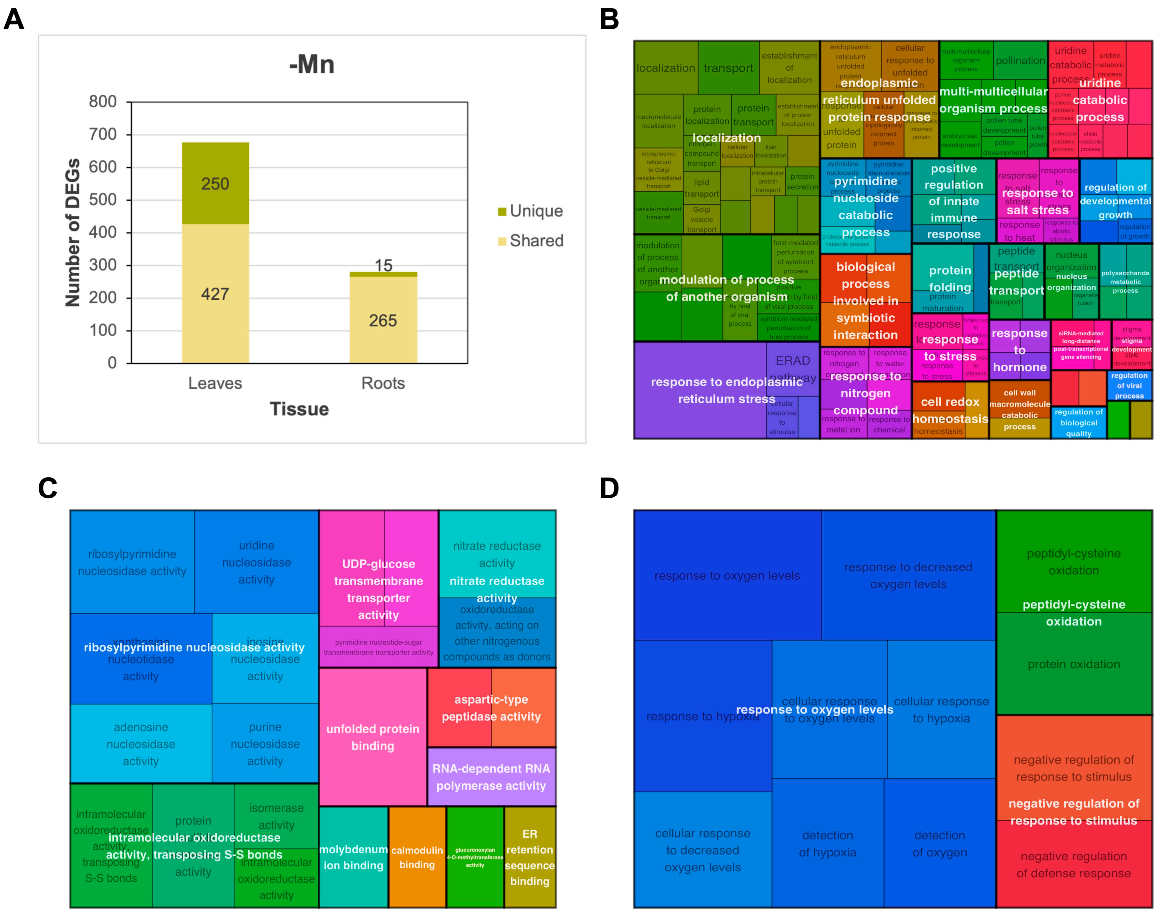

Supplemental Figure S39. Gene set enrichment analysis (GSEA) results for the manganese-deficient leaf transcriptome. (A) Treemap of enriched and semantically clustered biological process (BP) Gene Ontology terms. (B) Treemap of enriched and semantically clustered molecular function (MF) Gene Ontology terms. (C) Dot plot of enriched KEGG pathways, faceted by direction of enrichment, with dot size proportional to the number of genes in each pathway and dot color indicating adjusted p-value. (D) Pathview rendering of the photosynthesis – antenna proteins KEGG pathway, with genes colored by log_2FC on a blue to red scale, where blue indicates downregulation and red indicates upregulation. GSEA was performed using the GSEA function in clusterProfiler with Benjamini-Hochberg correction and a p-value cutoff of 0.05. Pathway visualization was performed using Pathview.

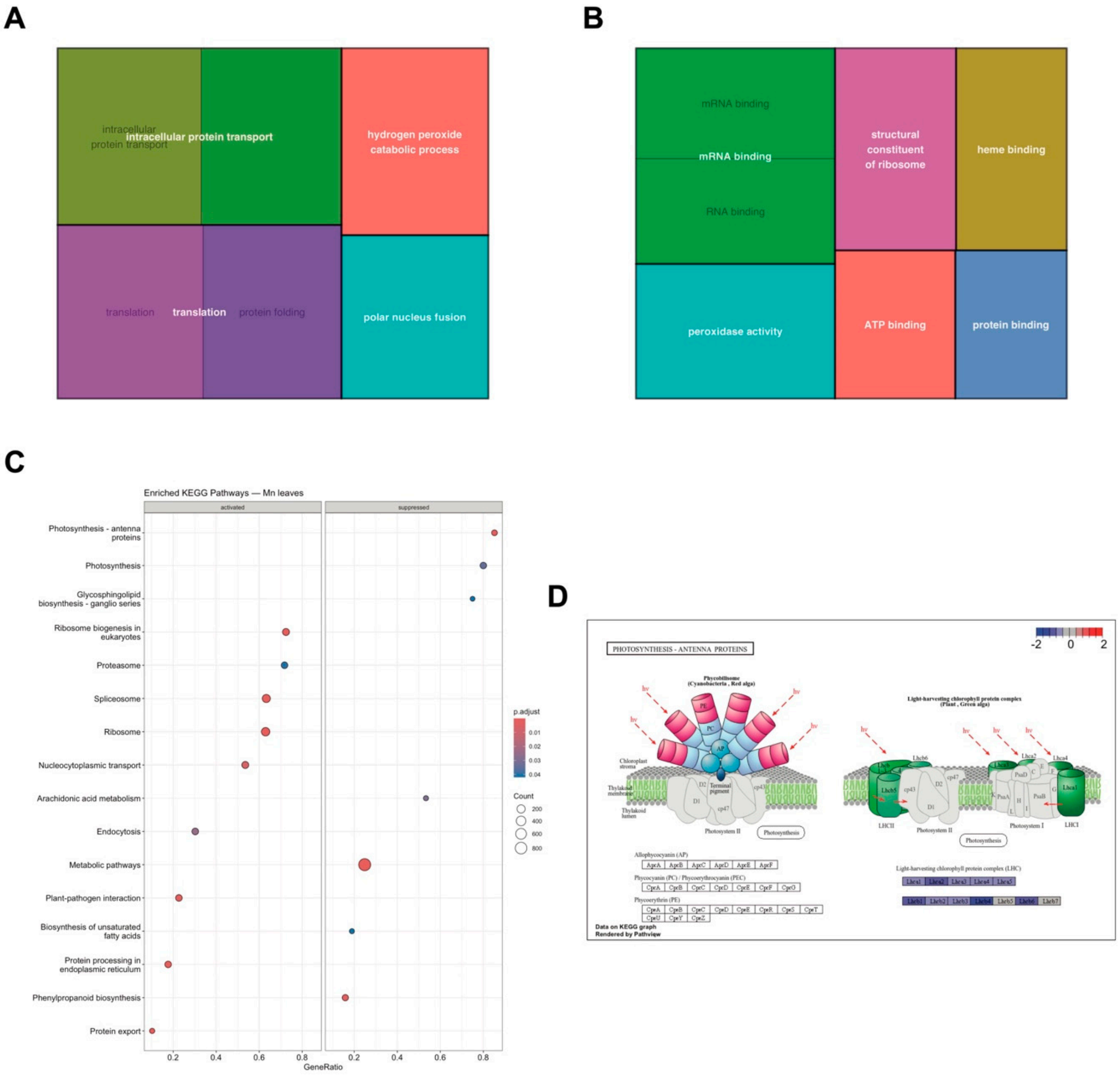

Supplemental Figure S40. Partial least squares discriminant analysis (PLS-DA) of manganese-deficient and control transcriptomes in leaves and roots. (A) Individuals plot for leaf samples, with each point representing a biological replicate colored by treatment group and 95% confidence ellipses shown for each group. The percentage of variance explained by each component is indicated on the respective axis. (B) Table of the top 20 genes ranked by variable importance in projection (VIP) score on component 1 for leaf samples, with gene name and VIP score shown. (C) Treemap of enriched and semantically clustered biological process (BP) Gene Ontology terms for leaf transcripts with a VIP score ≥ 1.5. (D) Treemap of enriched and semantically clustered molecular function (MF) Gene Ontology terms for leaf transcripts with a VIP score ≥ 1.5. (E) Individuals plot for root samples, with each point representing a biological replicate colored by treatment group and 95% confidence ellipses shown for each group. (F) Table of the top 20 genes ranked by VIP score on component 1 for root samples. (G) Treemap of

enriched and semantically clustered BP Gene Ontology terms for root transcripts with a VIP score ≥ 1.5. (H) Treemap of enriched and semantically clustered MF Gene Ontology terms for root transcripts with a VIP score ≥ 1.5. PLS-DA was performed using the mixOmics package in R and GO term enrichment was performed using TRAPID with semantic clustering by rrvgo.

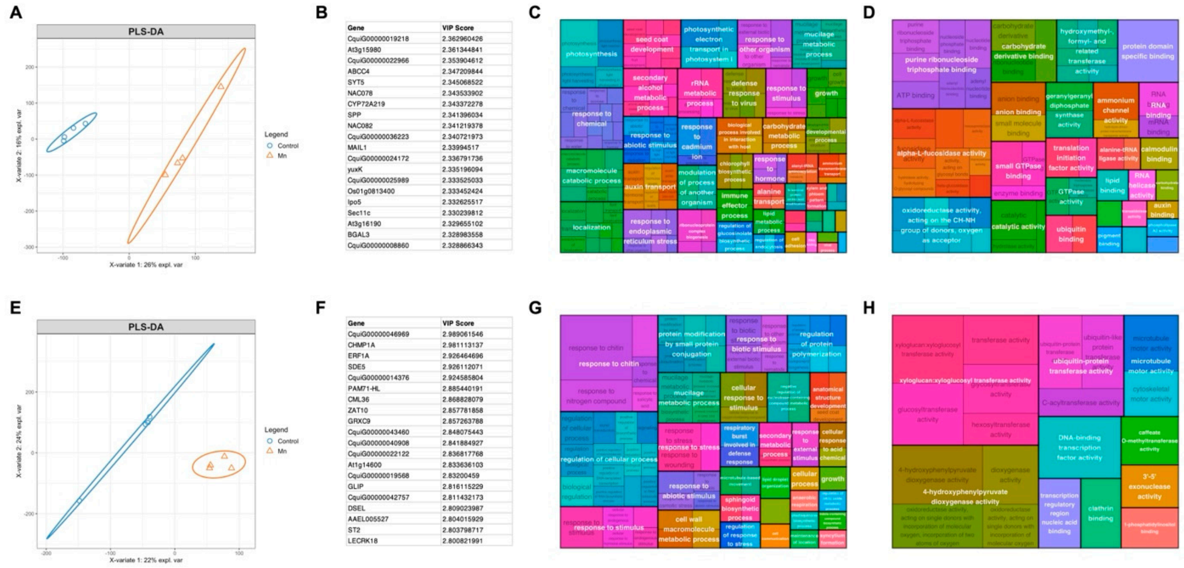

Supplemental Figure S41. Co-expression module characterization for module eigengenes strongly correlated with manganese deficiency in leaves. (A) Treemap of enriched and semantically clustered biological process (BP) Gene Ontology terms for ME13 in leaves. (B) Treemap of enriched and semantically clustered molecular function (MF) Gene Ontology terms for ME13 in leaves. (C) Network plot of the top 20 hub genes in ME13 identified by intramodular connectivity, with node labels indicating gene names. GO term enrichment was performed using TRAPID and semantic clustering was performed using rrvgo. Module eigengenes were identified using the WGCNA package in R.

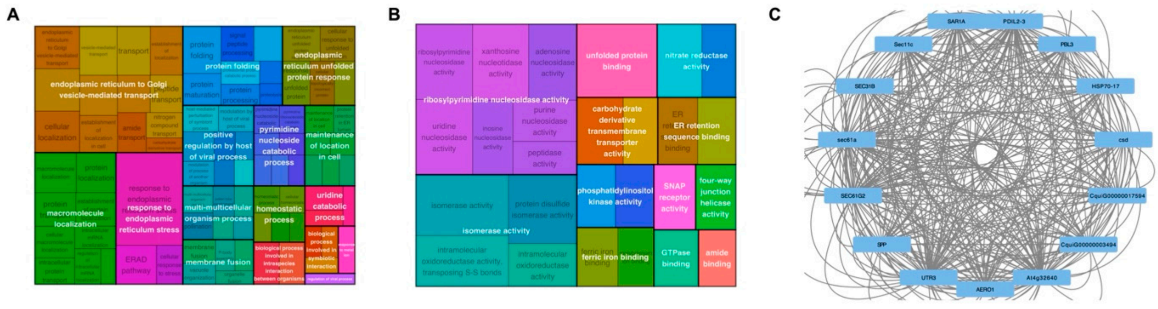

Supplemental Figure S42. Condition-specific differentially expressed genes (DEGs) under boron deficiency in leaves and roots. (A) Stacked bar chart showing the total number of DEGs in each tissue, partitioned into unique (green) and shared (yellow) fractions. (B) Treemap of

enriched and semantically clustered BP Gene Ontology terms for boron-specific root DEGs. (C) Treemap of enriched and semantically clustered MF Gene Ontology terms for boron-specific root DEGs GO term enrichment was performed using TRAPID and semantic clustering was performed using rrvgo.

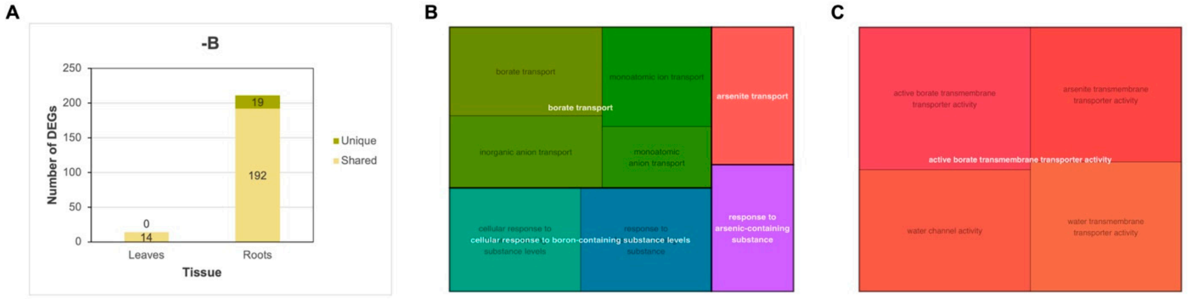

Supplemental Figure S43. Gene set enrichment analysis (GSEA) results for boron-deficient leaf and root transcriptomes. (A) Treemap of enriched and semantically clustered molecular function (MF) Gene Ontology terms in leaves. (B) Treemap of enriched and semantically clustered biological process (BP) Gene Ontology terms in roots. (C) Treemap of enriched and semantically clustered molecular function (MF) Gene Ontology terms in roots. (D) Dot plot of enriched KEGG pathways in roots, faceted by direction of enrichment, with dot size proportional to the number of genes in each pathway and dot color indicating adjusted p-value. (E) Pathview rendering of the protein processing in the endoplasmic reticulum KEGG pathway in roots, with genes colored by log_2FC on a blue to red scale, where blue indicates downregulation and red indicates upregulation. GSEA was performed using the GSEA function in clusterProfiler with Benjamini-Hochberg correction and a p-value cutoff of 0.05. Pathway visualization was performed using Pathview.

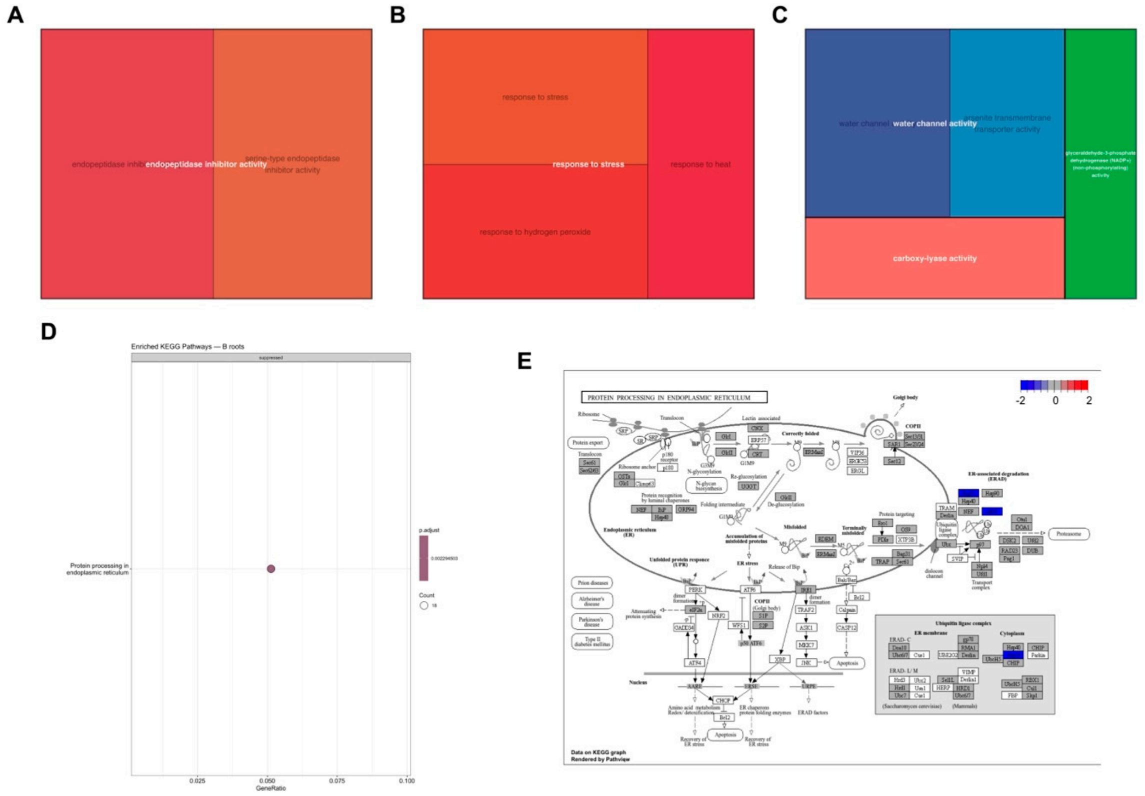

Supplemental Figure S44. Partial least squares discriminant analysis (PLS-DA) of boron-deficient and control transcriptomes in leaves and roots. (A) Individuals plot for leaf samples, with each point representing a biological replicate colored by treatment group and 95% confidence ellipses shown for each group. The percentage of variance explained by each component is indicated on the respective axis. (B) Table of the top 20 genes ranked by variable importance in projection (VIP) score on component 1 for leaf samples, with gene name and VIP score shown. (C) Treemap of enriched and semantically clustered biological process (BP) Gene Ontology terms for leaf transcripts with a VIP score ≥ 1.5. (D) Treemap of enriched and semantically clustered molecular function (MF) Gene Ontology terms for leaf transcripts with a VIP score ≥ 1.5. (E) Individuals plot for root samples, with each point representing a biological replicate colored by treatment group and 95% confidence ellipses shown for each group. (F) Table of the top 20 genes ranked by VIP score on component 1 for root samples. (G) Treemap of enriched and semantically clustered BP Gene Ontology terms for root transcripts with a VIP score ≥ 1.5. (H) Treemap of enriched and semantically clustered MF Gene Ontology terms for root transcripts with a VIP score ≥ 1.5. PLS-DA was performed using the mixOmics package in R and GO term enrichment was performed using TRAPID with semantic clustering by rrvgo.

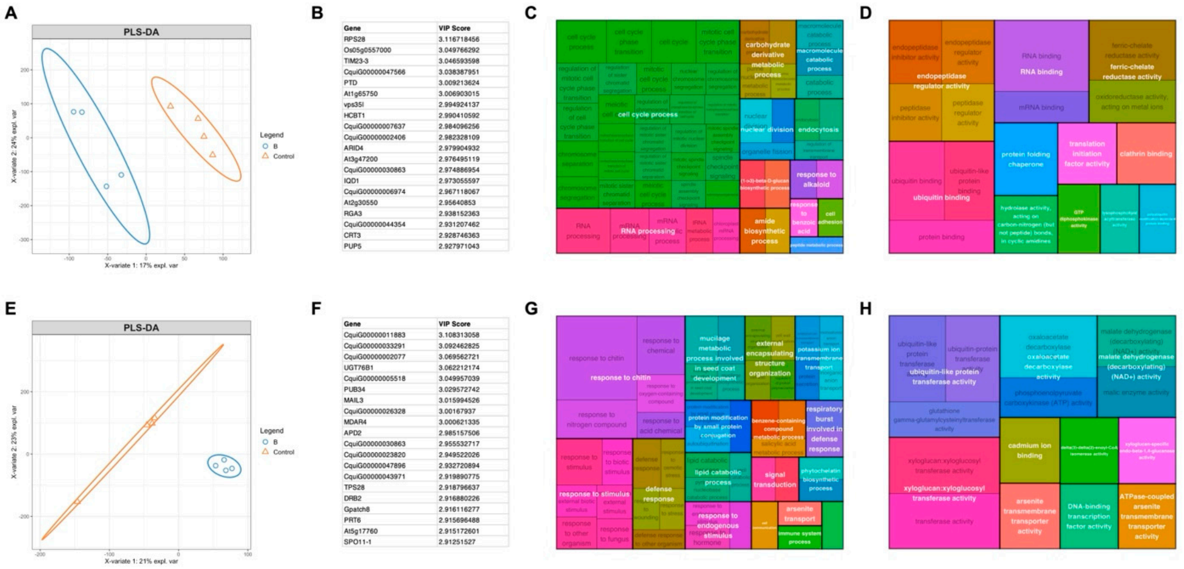

Supplemental Figure S45. Condition-specific differentially expressed genes (DEGs) under copper deficiency in leaves and roots. (A) Stacked bar chart showing the total number of DEGs in each tissue, partitioned into unique (green) and shared (yellow) fractions. (B) Treemap of enriched and semantically clustered biological process (BP) Gene Ontology terms for copper-specific leaf DEGs, with cluster size proportional to the number of genes associated with each term. (C) Treemap of enriched and semantically clustered molecular function (MF) Gene Ontology terms for copper-specific leaf DEGs. (D) Treemap of enriched and semantically clustered BP Gene Ontology terms for copper-specific root DEGs. (E) Treemap of enriched and semantically clustered MF Gene Ontology terms for copper-specific root DEGs. GO term enrichment was performed using TRAPID and semantic clustering was performed using rrvgo.

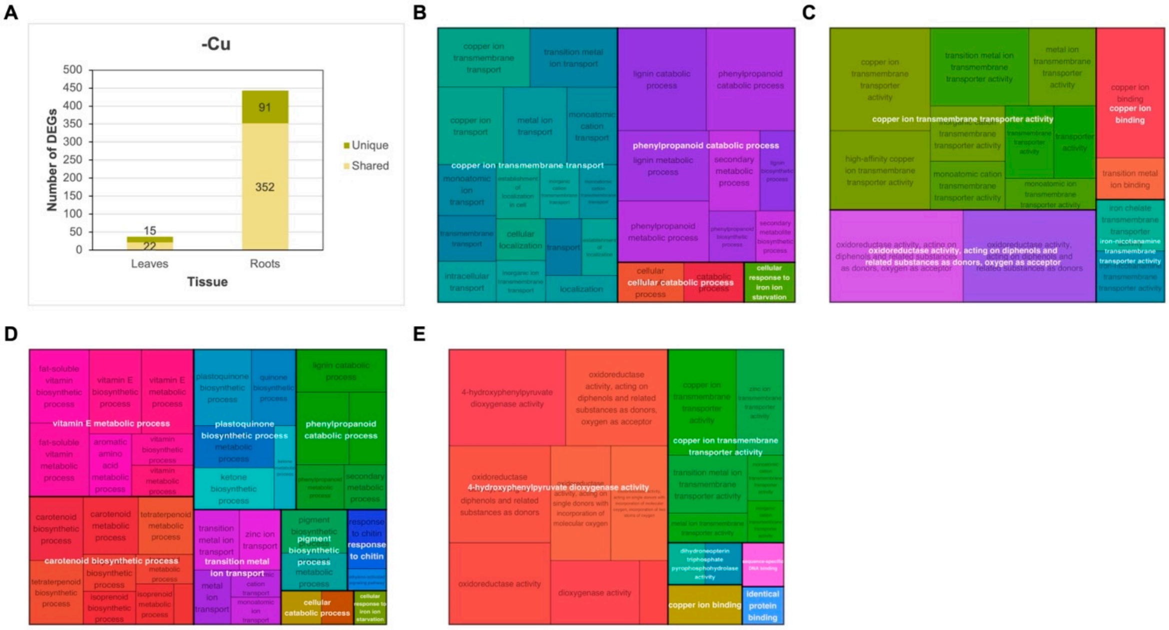

Supplemental Figure S46. Gene set enrichment analysis (GSEA) results for copper-deficient leaf transcriptomes. (A) Dot plot of enriched KEGG pathways, faceted by direction of enrichment, with dot size proportional to the number of genes in each pathway and dot color indicating adjusted p-value. (B) Pathview rendering of the lysine biosynthesis KEGG pathway, with genes colored by $\log_2$FC on a blue to red scale, where blue indicates downregulation and red indicates upregulation. GSEA was performed using the GSEA function in clusterProfiler with Benjamini-Hochberg correction and a p-value cutoff of 0.05. Pathway visualization was performed using Pathview.

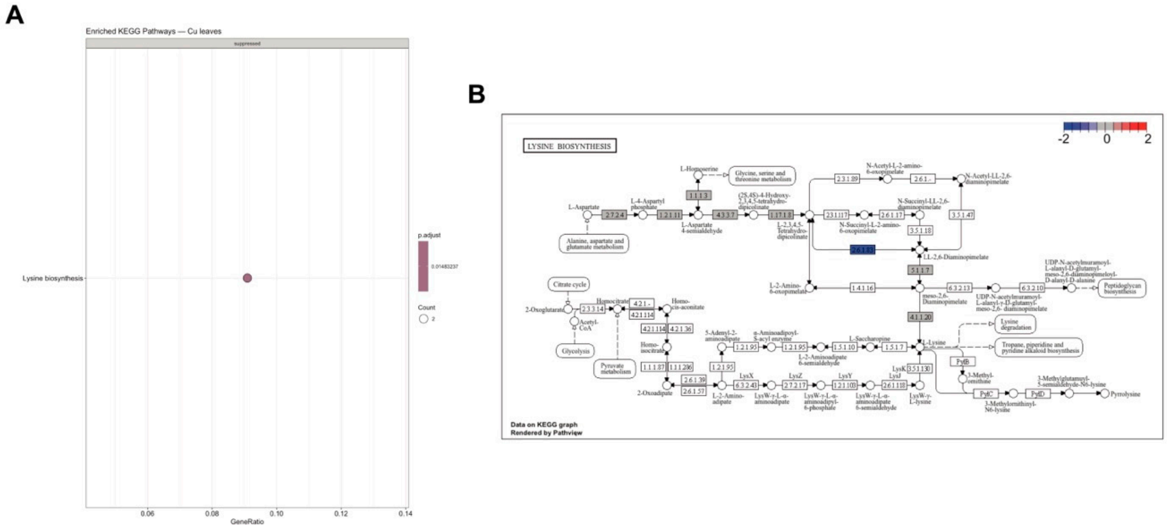

Supplemental Figure S47. Partial least squares discriminant analysis (PLS-DA) of copper-deficient and control transcriptomes in leaves and roots. (A) Individuals plot for leaf samples, with each point representing a biological replicate colored by treatment group and 95% confidence ellipses shown for each group. The percentage of variance explained by each component is indicated on the respective axis. (B) Table of the top 20 genes ranked by variable importance in projection (VIP) score on component 1 for leaf samples, with gene name and VIP score shown. (C) Treemap of enriched and semantically clustered biological process (BP) Gene Ontology terms for leaf transcripts with a VIP score ≥ 1.5. (D) Treemap of enriched and semantically clustered molecular function (MF) Gene Ontology terms for leaf transcripts with a VIP score ≥ 1.5. (E) Individuals plot for root samples, with each point representing a biological replicate colored by treatment group and 95% confidence ellipses shown for each group. (F) Table of the top 20 genes ranked by VIP score on component 1 for root samples. (G) Treemap of enriched and semantically clustered BP Gene Ontology terms for root transcripts with a VIP score ≥ 1.5. (H) Treemap of enriched and semantically clustered MF Gene Ontology terms for root transcripts with a VIP score ≥ 1.5. PLS-DA was performed using the mixOmics package in R and GO term enrichment was performed using TRAPID with semantic clustering by rrvgo.

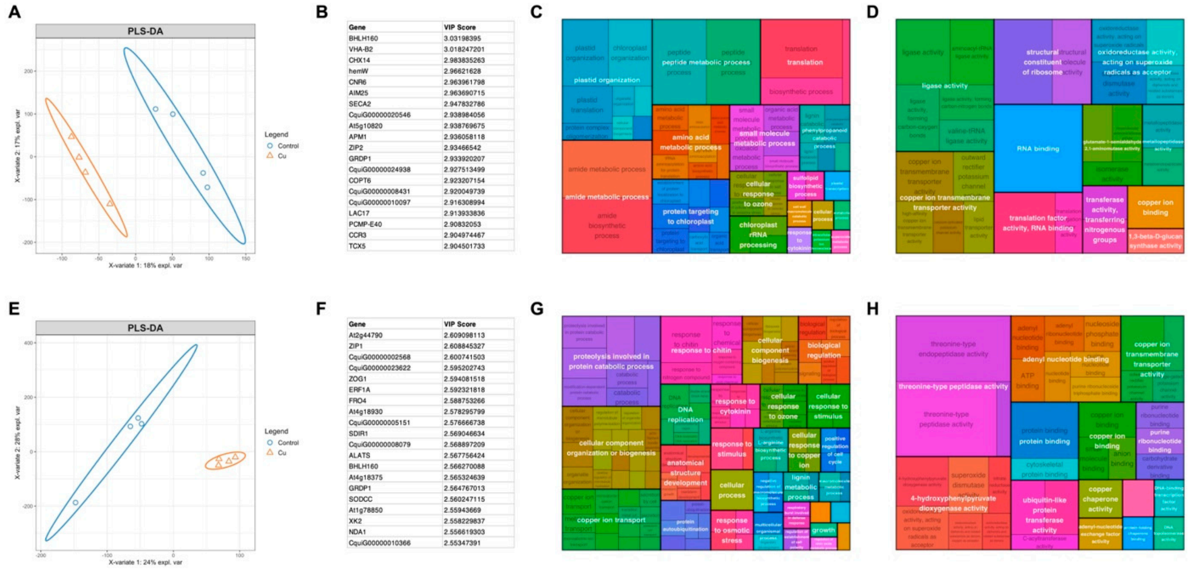

Supplemental Figure S48. Co-expression module characterization for module eigengenes strongly correlated with copper deficiency in roots. (A) Treemap of enriched and semantically clustered biological process (BP) Gene Ontology terms for ME36 in roots. (B) Treemap of enriched and semantically clustered molecular function (MF) Gene Ontology terms for ME36 in roots. (C) Network plot of the top 20 hub genes in ME36 identified by intramodular connectivity, with node labels indicating gene names. GO term enrichment was performed using TRAPID and semantic clustering was performed using rrvgo. Module eigengenes were identified using the WGCNA package in R.

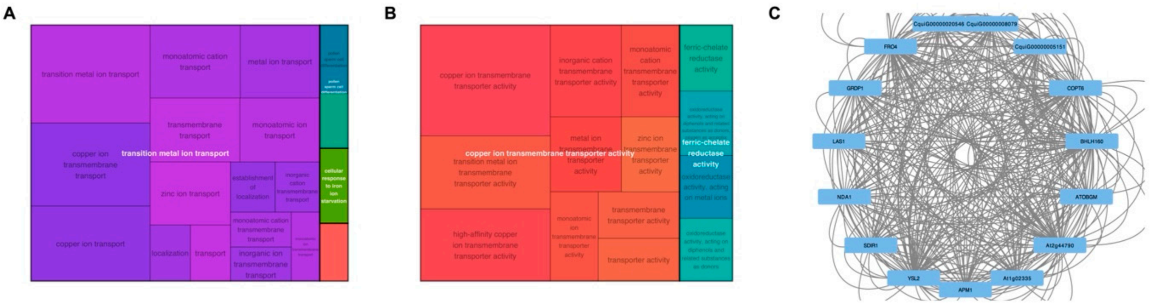

Supplemental Figure S49. Condition-specific differentially expressed genes (DEGs) under molybdenum deficiency in leaves and roots displayed as a stacked bar chart. The total number of DEGs in each tissue are partitioned into unique (green) and shared (yellow) fractions.

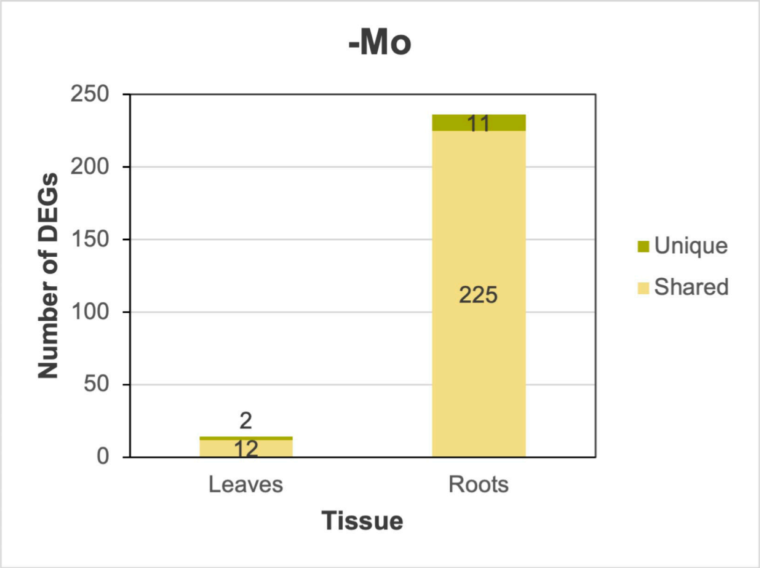

Supplemental Figure S50. Partial least squares discriminant analysis (PLS-DA) of molybdenum-deficient and control transcriptomes in leaves and roots. (A) Individuals plot for leaf samples, with each point representing a biological replicate colored by treatment group and 95% confidence ellipses shown for each group. The percentage of variance explained by each component is indicated on the respective axis. (B) Table of the top 20 genes ranked by variable importance in projection (VIP) score on component 1 for leaf samples, with gene name and VIP score shown. (C) Treemap of enriched and semantically clustered biological process (BP) Gene Ontology terms for leaf transcripts with a VIP score ≥ 1.5. (D) Treemap of enriched and semantically clustered molecular function (MF) Gene Ontology terms for leaf transcripts with a VIP score ≥ 1.5. (E) Individuals plot for root samples, with each point representing a biological replicate colored by treatment group and 95% confidence ellipses shown for each group. (F) Table of the top 20 genes ranked by VIP score on component 1 for root samples. (G) Treemap of enriched and semantically clustered BP Gene Ontology terms for root transcripts with a VIP

score ≥ 1.5. (H) Treemap of enriched and semantically clustered MF Gene Ontology terms for root transcripts with a VIP score ≥ 1.5. PLS-DA was performed using the mixOmics package in R and GO term enrichment was performed using TRAPID with semantic clustering by rrvgo.

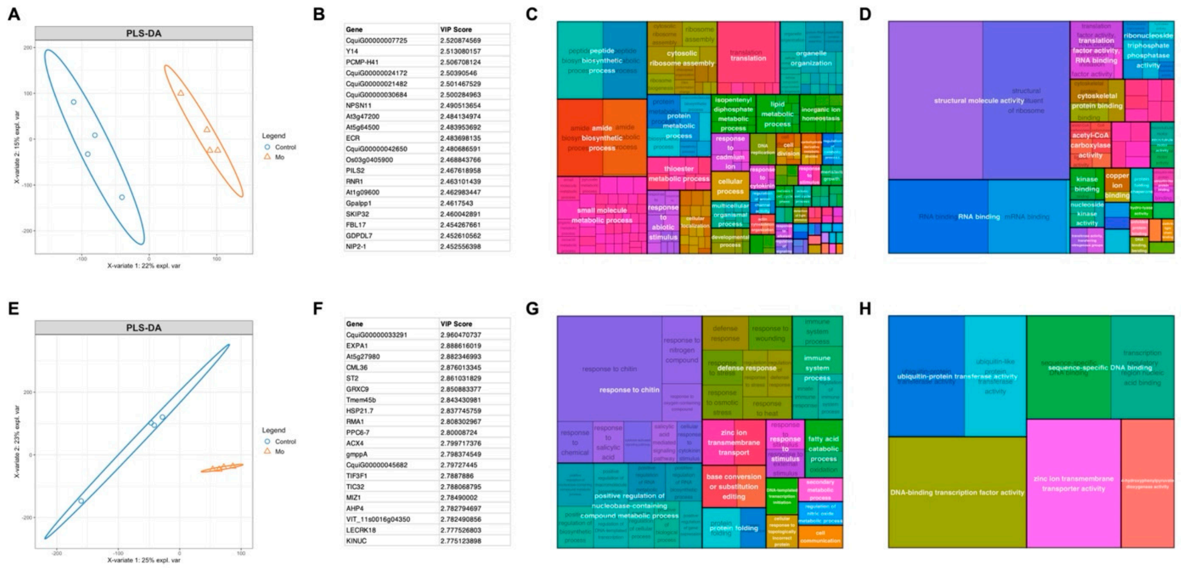

Supplement: Supplementary file 1 [file plants-15-02828-s001.zip › plants-4506197-supplementary.pdf]
